# Supplementary material for: Residue-by-residue analysis of cotranslational membrane protein integration in vivo
Source: eLife. 2021 Feb 8;10:e64302. doi: 10.7554/eLife.64302 (PMC7886326; doi:10.7554/eLife.64302)
Supplement: Supplementary file 1. [file elife-64302-supp1.docx]

EmrE C_out_ SecM(*Ec*)(N=45-170)

*N*

MNPYIYLGGAILAEVIGTTLMKFSEGFRRLWPSVGTIICYCASFWLLAQTLAYIPTGIAYAIWSGVGIVLISLLSWGFFGQRLDRPAIIGMMLICAGVLIINLLSASTPHSGSGGQYFMMGDNRDNSADSRYWGFVPEANWMSSYPYDVPDYAFSTPVWISQAQGIRAGPGSSDKQEGEWPTGLRLSRIGGIH 170

MNPYIYLGGAILAEVIGTTLMKFSEGFRRLWPSVGTIICYCASFWLLAQTLAYIPTGIAYAIWSGVGIVLISLLSWGFFGQRLDRPAIIGMMLICAGVLIINLLSASTPHSGSGGQYFMMGDNRDNSADSRYWGFVPSSYPYDVPDYAFSTPVWISQAQGIRAGPGSSDKQEGEWPTGLRLSRIGGIH 165

MNPYIYLGGAILAEVIGTTLMKFSEGFRRLWPSVGTIICYCASFWLLAQTLAYIPTGIAYAIWSGVGIVLISLLSWGFFGQRLDRPAIIGMMLICAGVLIINLLSASTPHSGSGGQYFMMGDNRDNSADSRYSSYPYDVPDYAFSTPVWISQAQGIRAGPGSSDKQEGEWPTGLRLSRIGGIH 160

MNPYIYLGGAILAEVIGTTLMKFSEGFRRLWPSVGTIICYCASFWLLAQTLAYIPTGIAYAIWSGVGIVLISLLSWGFFGQRLDRPAIIGMMLICAGVLIINLLSASTPHSGSGGQYFMMGDNRDNSSSYPYDVPDYAFSTPVWISQAQGIRAGPGSSDKQEGEWPTGLRLSRIGGIH 155

MNPYIYLGGAILAEVIGTTLMKFSEGFRRLWPSVGTIICYCASFWLLAQTLAYIPTGIAYAIWSGVGIVLISLLSWGFFGQRLDRPAIIGMMLICAGVLIINLLSASTPHSGSGGQYFMMGDSSYPYDVPDYAFSTPVWISQAQGIRAGPGSSDKQEGEWPTGLRLSRIGGIH 150

MNPYIYLGGAILAEVIGTTLMKFSEGFRRLWPSVGTIICYCASFWLLAQTLAYIPTGIAYAIWSGVGIVLISLLSWGFFGQRLDRPAIIGMMLICAGVLIINLLSASTPHSGSGGQYSSYPYDVPDYAFSTPVWISQAQGIRAGPGSSDKQEGEWPTGLRLSRIGGIH 145

MNPYIYLGGAILAEVIGTTLMKFSEGFRRLWPSVGTIICYCASFWLLAQTLAYIPTGIAYAIWSGVGIVLISLLSWGFFGQRLDRPAIIGMMLICAGVLIINLLSASTPHSGSGYPYDVPDYAFSTPVWISQAQGIRAGPGSSDKQEGEWPTGLRLSRIGGIH 140

MNPYIYLGGAILAEVIGTTLMKFSEGFRRLWPSVGTIICYCASFWLLAQTLAYIPTGIAYAIWSGVGIVLISLLSWGFFGQRLDRPAIIGMMLICAGVLIINLLSASSGSGYPYDVPDYAFSTPVWISQAQGIRAGPGSSDKQEGEWPTGLRLSRIGGIH 137

MNPYIYLGGAILAEVIGTTLMKFSEGFRRLWPSVGTIICYCASFWLLAQTLAYIPTGIAYAIWSGVGIVLISLLSWGFFGQRLDRPAIIGMMLICAGVLIINLLSSGSGYPYDVPDYAFSTPVWISQAQGIRAGPGSSDKQEGEWPTGLRLSRIGGIH 135

MNPYIYLGGAILAEVIGTTLMKFSEGFRRLWPSVGTIICYCASFWLLAQTLAYIPTGIAYAIWSGVGIVLISLLSWGFFGQRLDRPAIIGMMLICAGVLIINSGSGYPYDVPDYAFSTPVWISQAQGIRAGPGSSDKQEGEWPTGLRLSRIGGIH 132

MNPYIYLGGAILAEVIGTTLMKFSEGFRRLWPSVGTIICYCASFWLLAQTLAYIPTGIAYAIWSGVGIVLISLLSWGFFGQRLDRPAIIGMMLICAGVLISGSGYPYDVPDYAFSTPVWISQAQGIRAGPGSSDKQEGEWPTGLRLSRIGGIH 130

MNPYIYLGGAILAEVIGTTLMKFSEGFRRLWPSVGTIICYCASFWLLAQTLAYIPTGIAYAIWSGVGIVLISLLSWGFFGQRLDRPAIIGMMLICAGSGSGYPYDVPDYAFSTPVWISQAQGIRAGPGSSDKQEGEWPTGLRLSRIGGIH 127

MNPYIYLGGAILAEVIGTTLMKFSEGFRRLWPSVGTIICYCASFWLLAQTLAYIPTGIAYAIWSGVGIVLISLLSWGFFGQRLDRPAIIGMMLICSGSGYPYDVPDYAFSTPVWISQAQGIRAGPGSSDKQEGEWPTGLRLSRIGGIH 125

MNPYIYLGGAILAEVIGTTLMKFSEGFRRLWPSVGTIICYCASFWLLAQTLAYIPTGIAYAIWSGVGIVLISLLSWGFFGQRLDRPAIIGMMSGSGYPYDVPDYAFSTPVWISQAQGIRAGPGSSDKQEGEWPTGLRLSRIGGIH 122

MNPYIYLGGAILAEVIGTTLMKFSEGFRRLWPSVGTIICYCASFWLLAQTLAYIPTGIAYAIWSGVGIVLISLLSWGFFGQRLDRPAIIGSGSGYPYDVPDYAFSTPVWISQAQGIRAGPGSSDKQEGEWPTGLRLSRIGGIH 120

MNPYIYLGGAILAEVIGTTLMKFSEGFRRLWPSVGTIICYCASFWLLAQTLAYIPTGIAYAIWSGVGIVLISLLSWGFFGQRLDRPASGSGYPYDVPDYAFSTPVWISQAQGIRAGPGSSDKQEGEWPTGLRLSRIGGIH 117

MNPYIYLGGAILAEVIGTTLMKFSEGFRRLWPSVGTIICYCASFWLLAQTLAYIPTGIAYAIWSGVGIVLISLLSWGFFGQRLDRSGSGYPYDVPDYAFSTPVWISQAQGIRAGPGSSDKQEGEWPTGLRLSRIGGIH 115

MNPYIYLGGAILAEVIGTTLMKFSEGFRRLWPSVGTIICYCASFWLLAQTLAYIPTGIAYAIWSGVGIVLISLLSWGFFGQRSGSGYPYDVPDYAFSTPVWISQAQGIRAGPGSSDKQEGEWPTGLRLSRIGGIH 112

MNPYIYLGGAILAEVIGTTLMKFSEGFRRLWPSVGTIICYCASFWLLAQTLAYIPTGIAYAIWSGVGIVLISLLSWGFFGSGSGYPYDVPDYAFSTPVWISQAQGIRAGPGSSDKQEGEWPTGLRLSRIGGIH 110

MNPYIYLGGAILAEVIGTTLMKFSEGFRRLWPSVGTIICYCASFWLLAQTLAYIPTGIAYAIWSGVGIVLISLLSWGSGSGYPYDVPDYAFSTPVWISQAQGIRAGPGSSDKQEGEWPTGLRLSRIGGIH 107

MNPYIYLGGAILAEVIGTTLMKFSEGFRRLWPSVGTIICYCASFWLLAQTLAYIPTGIAYAIWSGVGIVLISLLSSGSGYPYDVPDYAFSTPVWISQAQGIRAGPGSSDKQEGEWPTGLRLSRIGGIH 105

MNPYIYLGGAILAEVIGTTLMKFSEGFRRLWPSVGTIICYCASFWLLAQTLAYIPTGIAYAIWSGVGIVLISSGSGYPYDVPDYAFSTPVWISQAQGIRAGPGSSDKQEGEWPTGLRLSRIGGIH 102

MNPYIYLGGAILAEVIGTTLMKFSEGFRRLWPSVGTIICYCASFWLLAQTLAYIPTGIAYAIWSGVGIVLSGSGYPYDVPDYAFSTPVWISQAQGIRAGPGSSDKQEGEWPTGLRLSRIGGIH 100

MNPYIYLGGAILAEVIGTTLMKFSEGFRRLWPSVGTIICYCASFWLLAQTLAYIPTGIAYAIWSGSGSGYPYDVPDYAFSTPVWISQAQGIRAGPGSSDKQEGEWPTGLRLSRIGGIH 95

MNPYIYLGGAILAEVIGTTLMKFSEGFRRLWPSVGTIICYCASFWLLAQTLAYIPTGIAYSGSGYPYDVPDYAFSTPVWISQAQGIRAGPGSSDKQEGEWPTGLRLSRIGGIH 90

MNPYIYLGGAILAEVIGTTLMKFSEGFRRLWPSVGTIICYCASFWLLAQTLAYIPSGSGYPYDVPDYAFSTPVWISQAQGIRAGPGSSDKQEGEWPTGLRLSRIGGIH 85

MNPYIYLGGAILAEVIGTTLMKFSEGFRRLWPSVGTIICYCASFWLLAQTSGSGYPYDVPDYAFSTPVWISQAQGIRAGPGSSDKQEGEWPTGLRLSRIGGIH 80

MNPYIYLGGAILAEVIGTTLMKFSEGFRRLWPSVGTIICYCASFWSGSGYPYDVPDYAFSTPVWISQAQGIRAGPGSSDKQEGEWPTGLRLSRIGGIH 75

MNPYIYLGGAILAEVIGTTLMKFSEGFRRLWPSVGTIICYSGSGYPYDVPDYAFSTPVWISQAQGIRAGPGSSDKQEGEWPTGLRLSRIGGIH 70

MNPYIYLGGAILAEVIGTTLMKFSEGFRRLWPSVGSGSGYPYDVPDYAFSTPVWISQAQGIRAGPGSSDKQEGEWPTGLRLSRIGGIH 65

MNPYIYLGGAILAEVIGTTLMKFSEGFRRLSGSGYPYDVPDYAFSTPVWISQAQGIRAGPGSSDKQEGEWPTGLRLSRIGGIH 60

MNPYIYLGGAILAEVIGTTLMKFSESGSGYPYDVPDYAFSTPVWISQAQGIRAGPGSSDKQEGEWPTGLRLSRIGGIH 55

MNPYIYLGGAILAEVIGTTLSGSGYPYDVPDYAFSTPVWISQAQGIRAGPGSSDKQEGEWPTGLRLSRIGGIH 50

MNPYIYLGGAILAEVSGSGYPYDVPDYAFSTPVWISQAQGIRAGPGSSDKQEGEWPTGLRLSRIGGIH 45

EmrE C_out_ TMH

Linker sequence (LepB derived)

HA-tag

SecM AP

EmrE C_out_ SecM(*Ec*) E14L (N=45-170)

MNPYIYLGGAILALVIGTTLMKFSEGFRRLWPSVGTIICYCASFWLLAQTLAYIPTGIAYAIWSGVGIVLISLLSWGFFGQRLDRPAIIGMMLICAGVLIINLLSASTPHSGSGGQYFMMGDNRDNSADSRYWGFVPEANWMSSYPYDVPDYAFSTPVWISQAQGIRAGPGSSDKQEGEWPTGLRLSRIGGIH 170

MNPYIYLGGAILALVIGTTLMKFSEGFRRLWPSVGTIICYCASFWLLAQTLAYIPTGIAYAIWSGVGIVLISLLSWGFFGQRLDRPAIIGMMLICAGVLIINLLSASTPHSGSGGQYFMMGDNRDNSADSRYWGFVPSSYPYDVPDYAFSTPVWISQAQGIRAGPGSSDKQEGEWPTGLRLSRIGGIH 165

MNPYYIYLGGAILALVIGTTLMKFSEGFRRLWPSVGTIICYCASFWLLAQTLAYIPTGIAYAIWSGVGIVLISLLSWGFFGQRLDRPAIIGMMLICAGVLIINLLSASTPHSGSGGQYFMMGDNRDNSADSRYSSYPYDVPDYAFSTPVWISQAQGIRAGPGSSDKQEGEWPTGLRLSRIGGIH 160

MNPYYIYLGGAILALVIGTTLMKFSEGFRRLWPSVGTIICYCASFWLLAQTLAYIPTGIAYAIWSGVGIVLISLLSWGFFGQRLDRPAIIGMMLICAGVLIINLLSASTPHSGSGGQYFMMGDNRDNSSSYPYDVPDYAFSTPVWISQAQGIRAGPGSSDKQEGEWPTGLRLSRIGGIH 155

MNPYIYLGGAILALVIGTTLMKFSEGFRRLWPSVGTIICYCASFWLLAQTLAYIPTGIAYAIWSGVGIVLISLLSWGFFGQRLDRPAIIGMMLICAGVLIINLLSASTPHSGSGGQYFMMGDSSYPYDVPDYAFSTPVWISQAQGIRAGPGSSDKQEGEWPTGLRLSRIGGIH 150

MNPYIYLGGAILALVIGTTLMKFSEGFRRLWPSVGTIICYCASFWLLAQTLAYIPTGIAYAIWSGVGIVLISLLSWGFFGQRLDRPAIIGMMLICAGVLIINLLSASTPHSGSGGQYSSYPYDVPDYAFSTPVWISQAQGIRAGPGSSDKQEGEWPTGLRLSRIGGIH 145

MNPYIYLGGAILALVIGTTLMKFSEGFRRLWPSVGTIICYCASFWLLAQTLAYIPTGIAYAIWSGVGIVLISLLSWGFFGQRLDRPAIIGMMLICAGVLIINLLSASTPHSGSGYPYDVPDYAFSTPVWISQAQGIRAGPGSSDKQEGEWPTGLRLSRIGGIH 140

MNPYIYLGGAILALVIGTTLMKFSEGFRRLWPSVGTIICYCASFWLLAQTLAYIPTGIAYAIWSGVGIVLISLLSWGFFGQRLDRPAIIGMMLICAGVLIINLLSASSGSGYPYDVPDYAFSTPVWISQAQGIRAGPGSSDKQEGEWPTGLRLSRIGGIH 137

MNPYIYLGGAILALVIGTTLMKFSEGFRRLWPSVGTIICYCASFWLLAQTLAYIPTGIAYAIWSGVGIVLISLLSWGFFGQRLDRPAIIGMMLICAGVLIINLLSSGSGYPYDVPDYAFSTPVWISQAQGIRAGPGSSDKQEGEWPTGLRLSRIGGIH 135

MNPYIYLGGAILALVIGTTLMKFSEGFRRLWPSVGTIICYCASFWLLAQTLAYIPTGIAYAIWSGVGIVLISLLSWGFFGQRLDRPAIIGMMLICAGVLIINSGSGYPYDVPDYAFSTPVWISQAQGIRAGPGSSDKQEGEWPTGLRLSRIGGIH 132

MNPYIYLGGAILALVIGTTLMKFSEGFRRLWPSVGTIICYCASFWLLAQTLAYIPTGIAYAIWSGVGIVLISLLSWGFFGQRLDRPAIIGMMLICAGVLISGSGYPYDVPDYAFSTPVWISQAQGIRAGPGSSDKQEGEWPTGLRLSRIGGIH 130

MNPYIYLGGAILALVIGTTLMKFSEGFRRLWPSVGTIICYCASFWLLAQTLAYIPTGIAYAIWSGVGIVLISLLSWGFFGQRLDRPAIIGMMLICAGSGSGYPYDVPDYAFSTPVWISQAQGIRAGPGSSDKQEGEWPTGLRLSRIGGIH 127

MNPYIYLGGAILALVIGTTLMKFSEGFRRLWPSVGTIICYCASFWLLAQTLAYIPTGIAYAIWSGVGIVLISLLSWGFFGQRLDRPAIIGMMLICSGSGYPYDVPDYAFSTPVWISQAQGIRAGPGSSDKQEGEWPTGLRLSRIGGIH 125

MNPYIYLGGAILALVIGTTLMKFSEGFRRLWPSVGTIICYCASFWLLAQTLAYIPTGIAYAIWSGVGIVLISLLSWGFFGQRLDRPAIIGMMSGSGYPYDVPDYAFSTPVWISQAQGIRAGPGSSDKQEGEWPTGLRLSRIGGIH 122

MNPYIYLGGAILALVIGTTLMKFSEGFRRLWPSVGTIICYCASFWLLAQTLAYIPTGIAYAIWSGVGIVLISLLSWGFFGQRLDRPAIIGSGSGYPYDVPDYAFSTPVWISQAQGIRAGPGSSDKQEGEWPTGLRLSRIGGIH 120

MNPYIYLGGAILALVIGTTLMKFSEGFRRLWPSVGTIICYCASFWLLAQTLAYIPTGIAYAIWSGVGIVLISLLSWGFFGQRLDRPASGSGYPYDVPDYAFSTPVWISQAQGIRAGPGSSDKQEGEWPTGLRLSRIGGIH 117

MNPYIYLGGAILALVIGTTLMKFSEGFRRLWPSVGTIICYCASFWLLAQTLAYIPTGIAYAIWSGVGIVLISLLSWGFFGQRLDRSGSGYPYDVPDYAFSTPVWISQAQGIRAGPGSSDKQEGEWPTGLRLSRIGGIH 115

MNPYIYLGGAILALVIGTTLMKFSEGFRRLWPSVGTIICYCASFWLLAQTLAYIPTGIAYAIWSGVGIVLISLLSWGFFGQRSGSGYPYDVPDYAFSTPVWISQAQGIRAGPGSSDKQEGEWPTGLRLSRIGGIH 112

MNPYIYLGGAILALVIGTTLMKFSEGFRRLWPSVGTIICYCASFWLLAQTLAYIPTGIAYAIWSGVGIVLISLLSWGFFGSGSGYPYDVPDYAFSTPVWISQAQGIRAGPGSSDKQEGEWPTGLRLSRIGGIH 110

MNPYIYLGGAILALVIGTTLMKFSEGFRRLWPSVGTIICYCASFWLLAQTLAYIPTGIAYAIWSGVGIVLISLLSWGSGSGYPYDVPDYAFSTPVWISQAQGIRAGPGSSDKQEGEWPTGLRLSRIGGIH 107

MNPYIYLGGAILALVIGTTLMKFSEGFRRLWPSVGTIICYCASFWLLAQTLAYIPTGIAYAIWSGVGIVLISLLSSGSGYPYDVPDYAFSTPVWISQAQGIRAGPGSSDKQEGEWPTGLRLSRIGGIH 105

MNPYIYLGGAILALVIGTTLMKFSEGFRRLWPSVGTIICYCASFWLLAQTLAYIPTGIAYAIWSGVGIVLISSGSGYPYDVPDYAFSTPVWISQAQGIRAGPGSSDKQEGEWPTGLRLSRIGGIH 102

MNPYIYLGGAILALVIGTTLMKFSEGFRRLWPSVGTIICYCASFWLLAQTLAYIPTGIAYAIWSGVGIVLSGSGYPYDVPDYAFSTPVWISQAQGIRAGPGSSDKQEGEWPTGLRLSRIGGIH 100

MNPYIYLGGAILALVIGTTLMKFSEGFRRLWPSVGTIICYCASFWLLAQTLAYIPTGIAYAIWSGSGSGYPYDVPDYAFSTPVWISQAQGIRAGPGSSDKQEGEWPTGLRLSRIGGIH 95

MNPYIYLGGAILALVIGTTLMKFSEGFRRLWPSVGTIICYCASFWLLAQTLAYIPTGIAYSGSGYPYDVPDYAFSTPVWISQAQGIRAGPGSSDKQEGEWPTGLRLSRIGGIH 90

MNPYIYLGGAILALVIGTTLMKFSEGFRRLWPSVGTIICYCASFWLLAQTLAYIPSGSGYPYDVPDYAFSTPVWISQAQGIRAGPGSSDKQEGEWPTGLRLSRIGGIH 85

MNPYIYLGGAILALVIGTTLMKFSEGFRRLWPSVGTIICYCASFWLLAQTSGSGYPYDVPDYAFSTPVWISQAQGIRAGPGSSDKQEGEWPTGLRLSRIGGIH 80

MNPYIYLGGAILALVIGTTLMKFSEGFRRLWPSVGTIICYCASFWSGSGYPYDVPDYAFSTPVWISQAQGIRAGPGSSDKQEGEWPTGLRLSRIGGIH 75

MNPYIYLGGAILALVIGTTLMKFSEGFRRLWPSVGTIICYSGSGYPYDVPDYAFSTPVWISQAQGIRAGPGSSDKQEGEWPTGLRLSRIGGIH 70

MNPYIYLGGAILALVIGTTLMKFSEGFRRLWPSVGSGSGYPYDVPDYAFSTPVWISQAQGIRAGPGSSDKQEGEWPTGLRLSRIGGIH 65

MNPYIYLGGAILALVIGTTLMKFSEGFRRLSGSGYPYDVPDYAFSTPVWISQAQGIRAGPGSSDKQEGEWPTGLRLSRIGGIH 60

MNPYIYLGGAILALVIGTTLMKFSESGSGYPYDVPDYAFSTPVWISQAQGIRAGPGSSDKQEGEWPTGLRLSRIGGIH 55

MNPYIYLGGAILALVIGTTLSGSGYPYDVPDYAFSTPVWISQAQGIRAGPGSSDKQEGEWPTGLRLSRIGGIH 50

MNPYIYLGGAILALVSGSGYPYDVPDYAFSTPVWISQAQGIRAGPGSSDKQEGEWPTGLRLSRIGGIH 45

EmrE C_out_ SecM(*Ec*) E14A

MNPYIYLGGAILAAVIGTTLMKFSEGFRRLWPSVGTIICYCASFWLLAQTLAYIPTGIAYAIWSGVGIVLISLLSWGFFGQRLDRPAIIGMMLICAGVLISGSGYPYDVPDYAFSTPVWISQAQGIRAGPGSSDKQEGEWPTGLRLSRIGGIH 130

MNPYIYLGGAILAAVIGTTLMKFSEGFRRLWPSVGTIICYCASFWLLAQTLAYIPTGIAYAIWSGVGIVLISLLSWGFFGQRLDRSGSGYPYDVPDYAFSTPVWISQAQGIRAGPGSSDKQEGEWPTGLRLSRIGGIH 115

MNPYIYLGGAILAAVIGTTLMKFSEGFRRLWPSVGTIICYCASFWLLAQTLAYIPTGIAYAIWSGVGIVLISLLSSGSGYPYDVPDYAFSTPVWISQAQGIRAGPGSSDKQEGEWPTGLRLSRIGGIH 105

MNPYIYLGGAILAAVIGTTLMKFSEGFRRLWPSVGTIICYCASFWLLAQTLAYIPSGSGYPYDVPDYAFSTPVWISQAQGIRAGPGSSDKQEGEWPTGLRLSRIGGIH 85

MNPYIYLGGAILAAVIGTTLMKFSESGSGYPYDVPDYAFSTPVWISQAQGIRAGPGSSDKQEGEWPTGLRLSRIGGIH 55

EmrE C_out_ SecM(*Ec*) E14D

MNPYIYLGGAILADVIGTTLMKFSEGFRRLWPSVGTIICYCASFWLLAQTLAYIPTGIAYAIWSGVGIVLISLLSWGFFGQRLDRPAIIGMMLICAGVLISGSGYPYDVPDYAFSTPVWISQAQGIRAGPGSSDKQEGEWPTGLRLSRIGGIH 130

MNPYIYLGGAILADVIGTTLMKFSEGFRRLWPSVGTIICYCASFWLLAQTLAYIPTGIAYAIWSGVGIVLISLLSWGFFGQRLDRSGSGYPYDVPDYAFSTPVWISQAQGIRAGPGSSDKQEGEWPTGLRLSRIGGIH 115

MNPYIYLGGAILADVIGTTLMKFSEGFRRLWPSVGTIICYCASFWLLAQTLAYIPTGIAYAIWSGVGIVLISLLSSGSGYPYDVPDYAFSTPVWISQAQGIRAGPGSSDKQEGEWPTGLRLSRIGGIH 105

MNPYIYLGGAILADVIGTTLMKFSEGFRRLWPSVGTIICYCASFWLLAQTLAYIPSGSGYPYDVPDYAFSTPVWISQAQGIRAGPGSSDKQEGEWPTGLRLSRIGGIH 85

MNPYIYLGGAILADVIGTTLMKFSESGSGYPYDVPDYAFSTPVWISQAQGIRAGPGSSDKQEGEWPTGLRLSRIGGIH 55

EmrE C_out_ SecM(*Ec*) E14Q

MNPYIYLGGAILAQVIGTTLMKFSEGFRRLWPSVGTIICYCASFWLLAQTLAYIPTGIAYAIWSGVGIVLISLLSWGFFGQRLDRPAIIGMMLICAGVLISGSGYPYDVPDYAFSTPVWISQAQGIRAGPGSSDKQEGEWPTGLRLSRIGGIH 130

MNPYIYLGGAILAQVIGTTLMKFSEGFRRLWPSVGTIICYCASFWLLAQTLAYIPTGIAYAIWSGVGIVLISLLSWGFFGQRLDRSGSGYPYDVPDYAFSTPVWISQAQGIRAGPGSSDKQEGEWPTGLRLSRIGGIH 115

MNPYIYLGGAILAQVIGTTLMKFSEGFRRLWPSVGTIICYCASFWLLAQTLAYIPTGIAYAIWSGVGIVLISLLSSGSGYPYDVPDYAFSTPVWISQAQGIRAGPGSSDKQEGEWPTGLRLSRIGGIH 105

MNPYIYLGGAILAQVIGTTLMKFSEGFRRLWPSVGTIICYCASFWLLAQTLAYIPSGSGYPYDVPDYAFSTPVWISQAQGIRAGPGSSDKQEGEWPTGLRLSRIGGIH 85

MNPYIYLGGAILAQVIGTTLMKFSESGSGYPYDVPDYAFSTPVWISQAQGIRAGPGSSDKQEGEWPTGLRLSRIGGIH 55

EmrE C_out_ SecM(*Ec*-Sup1)(N=100-145)

MNPYIYLGGAILAEVIGTTLMKFSEGFRRLWPSVGTIICYCASFWLLAQTLAYIPTGIAYAIWSGVGIVLISLLSWGFFGQRLDRPAIIGMMLICAGVLIINLLSASTPHSGSGGQYSSYPYDVPDYAFSTPVWISQAPPIRAGPGSSDKQEGEWPTGLRLSRIGGIH 145

MNPYIYLGGAILAEVIGTTLMKFSEGFRRLWPSVGTIICYCASFWLLAQTLAYIPTGIAYAIWSGVGIVLISLLSWGFFGQRLDRPAIIGMMLICAGVLIINLLSASTPHSGSGYPYDVPDYAFSTPVWISQAPPIRAGPGSSDKQEGEWPTGLRLSRIGGIH 140

MNPYIYLGGAILAEVIGTTLMKFSEGFRRLWPSVGTIICYCASFWLLAQTLAYIPTGIAYAIWSGVGIVLISLLSWGFFGQRLDRPAIIGMMLICAGVLIINLLSASSGSGYPYDVPDYAFSTPVWISQAPPIRAGPGSSDKQEGEWPTGLRLSRIGGIH 137

MNPYIYLGGAILAEVIGTTLMKFSEGFRRLWPSVGTIICYCASFWLLAQTLAYIPTGIAYAIWSGVGIVLISLLSWGFFGQRLDRPAIIGMMLICAGVLIINLLSSGSGYPYDVPDYAFSTPVWISQAPPIRAGPGSSDKQEGEWPTGLRLSRIGGIH 135

MNPYIYLGGAILAEVIGTTLMKFSEGFRRLWPSVGTIICYCASFWLLAQTLAYIPTGIAYAIWSGVGIVLISLLSWGFFGQRLDRPAIIGMMLICAGVLIINSGSGYPYDVPDYAFSTPVWISQAPPIRAGPGSSDKQEGEWPTGLRLSRIGGIH 132

MNPYIYLGGAILAEVIGTTLMKFSEGFRRLWPSVGTIICYCASFWLLAQTLAYIPTGIAYAIWSGVGIVLISLLSWGFFGQRLDRPAIIGMMLICAGVLISGSGYPYDVPDYAFSTPVWISQAPPIRAGPGSSDKQEGEWPTGLRLSRIGGIH 130

MNPYIYLGGAILAEVIGTTLMKFSEGFRRLWPSVGTIICYCASFWLLAQTLAYIPTGIAYAIWSGVGIVLISLLSWGFFGQRLDRPAIIGMMLICAGSGSGYPYDVPDYAFSTPVWISQAPPIRAGPGSSDKQEGEWPTGLRLSRIGGIH 127

MNPYIYLGGAILAEVIGTTLMKFSEGFRRLWPSVGTIICYCASFWLLAQTLAYIPTGIAYAIWSGVGIVLISLLSWGFFGQRLDRPAIIGMMLICSGSGYPYDVPDYAFSTPVWISQAPPIRAGPGSSDKQEGEWPTGLRLSRIGGIH 125

MNPYIYLGGAILAEVIGTTLMKFSEGFRRLWPSVGTIICYCASFWLLAQTLAYIPTGIAYAIWSGVGIVLISLLSWGFFGQRLDRPAIIGMMSGSGYPYDVPDYAFSTPVWISQAPPIRAGPGSSDKQEGEWPTGLRLSRIGGIH 122

MNPYIYLGGAILAEVIGTTLMKFSEGFRRLWPSVGTIICYCASFWLLAQTLAYIPTGIAYAIWSGVGIVLISLLSWGFFGQRLDRPAIIGSGSGYPYDVPDYAFSTPVWISQAPPIRAGPGSSDKQEGEWPTGLRLSRIGGIH 120

MNPYIYLGGAILAEVIGTTLMKFSEGFRRLWPSVGTIICYCASFWLLAQTLAYIPTGIAYAIWSGVGIVLISLLSWGFFGQRLDRPASGSGYPYDVPDYAFSTPVWISQAPPIRAGPGSSDKQEGEWPTGLRLSRIGGIH 117

MNPYIYLGGAILAEVIGTTLMKFSEGFRRLWPSVGTIICYCASFWLLAQTLAYIPTGIAYAIWSGVGIVLISLLSWGFFGQRLDRSGSGYPYDVPDYAFSTPVWISQAPPIRAGPGSSDKQEGEWPTGLRLSRIGGIH 115

MNPYIYLGGAILAEVIGTTLMKFSEGFRRLWPSVGTIICYCASFWLLAQTLAYIPTGIAYAIWSGVGIVLISLLSWGFFGQRSGSGYPYDVPDYAFSTPVWISQAPPIRAGPGSSDKQEGEWPTGLRLSRIGGIH 112

MNPYIYLGGAILAEVIGTTLMKFSEGFRRLWPSVGTIICYCASFWLLAQTLAYIPTGIAYAIWSGVGIVLISLLSWGFFGSGSGYPYDVPDYAFSTPVWISQAPPIRAGPGSSDKQEGEWPTGLRLSRIGGIH 110

MNPYIYLGGAILAEVIGTTLMKFSEGFRRLWPSVGTIICYCASFWLLAQTLAYIPTGIAYAIWSGVGIVLISLLSWGSGSGYPYDVPDYAFSTPVWISQAPPIRAGPGSSDKQEGEWPTGLRLSRIGGIH 107

MNPYIYLGGAILAEVIGTTLMKFSEGFRRLWPSVGTIICYCASFWLLAQTLAYIPTGIAYAIWSGVGIVLISLLSSGSGYPYDVPDYAFSTPVWISQAPPIRAGPGSSDKQEGEWPTGLRLSRIGGIH 105

MNPYIYLGGAILAEVIGTTLMKFSEGFRRLWPSVGTIICYCASFWLLAQTLAYIPTGIAYAIWSGVGIVLISSGSGYPYDVPDYAFSTPVWISQAPPIRAGPGSSDKQEGEWPTGLRLSRIGGIH 102

MNPYIYLGGAILAEVIGTTLMKFSEGFRRLWPSVGTIICYCASFWLLAQTLAYIPTGIAYAIWSGVGIVLSGSGYPYDVPDYAFSTPVWISQAPPIRAGPGSSDKQEGEWPTGLRLSRIGGIH 100

EmrE C_out_ SecM(*Ec*)I^37^I^38^→NN

MNPYIYLGGAILAEVIGTTLMKFSEGFRRLWPSVGTNNCYCASFWLLAQTLAYIPSGSGYPYDVPDYAFSTPVWISQAQGIRAGPGSSDKQEGEWPTGLRLSRIGGIH 85

MNPYIYLGGAILAEVIGTTLMKFSEGFRRLWPSVGTNNCYCASFWLLAQTSGSGYPYDVPDYAFSTPVWISQAQGIRAGPGSSDKQEGEWPTGLRLSRIGGIH 80

GlpG SecM(*Ec*) (N=46-336)

NTD

TM1 TM2 TM3 TM4 TM5 TM6 HA-tag SecM (*Ec*) Lep P2 *N* M

MLMITSFANPRVAQAFVDYMATQGVILTIQQHNQSDVWLADESQAERVRADVARFLENPADPRYLAASWQAGHTGSGLHYRRYPFFAALRERAGP**VTWVMMIACVVVFIAMQI**LGDQEVMLWLAWPFDPTLKFEFWRYFTHALMHFS**LMHILFNLLWWWYLGGAVEKRL**G**SGKLIVITLISALLSGYVQQKFTG**PWFGG**LSGVVYALMGYVWLRGERDPQSGI**YL**QRGLIIFALIWIVAGWF**DLFGMSM**ANGAHIAGLAVGLAMAFVDS**LNARKRKSGSGGQYFMMGDNRDNSADSRYWGFVPEANWMSSYPYDVPDYAFSTPVWISQAQGIRAGPGSSDKQEGEWPTGLRLSRIGGIH **336**

MLMITSFANPRVAQAFVDYMATQGVILTIQQHNQSDVWLADESQAERVRADVARFLENPADPRYLAASWQAGHTGSGLHYRRYPFFAALRERAGP**VTWVMMIACVVVFIAMQI**LGDQEVMLWLAWPFDPTLKFEFWRYFTHALMHFS**LMHILFNLLWWWYLGGAVEKRL**G**SGKLIVITLISALLSGYVQQKFTG**PWFGG**LSGVVYALMGYVWLRGERDPQSGI**YL**QRGLIIFALIWIVAGWF**DLFGMSM**ANGAHIAGLAVGLAMAFVDS**LNARKRKSGSGGQYFMMGDNRDNSADSRYWGFVPSSYPYDVPDYAFSTPVWISQAQGIRAGPGSSDKQEGEWPTGLRLSRIGGIH **331**

MLMITSFANPRVAQAFVDYMATQGVILTIQQHNQSDVWLADESQAERVRADVARFLENPADPRYLAASWQAGHTGSGLHYRRYPFFAALRERAGP**VTWVMMIACVVVFIAMQI**LGDQEVMLWLAWPFDPTLKFEFWRYFTHALMHFS**LMHILFNLLWWWYLGGAVEKRL**G**SGKLIVITLISALLSGYVQQKFTG**PWFGG**LSGVVYALMGYVWLRGERDPQSGI**YL**QRGLIIFALIWIVAGWF**DLFGMSM**ANGAHIAGLAVGLAMAFVDS**LNARKRKSGSGGQYFMMGDNRDNSADSRYSSYPYDVPDYAFSTPVWISQAQGIRAGPGSSDKQEGEWPTGLRLSRIGGIH  **326**

MLMITSFANPRVAQAFVDYMATQGVILTIQQHNQSDVWLADESQAERVRADVARFLENPADPRYLAASWQAGHTGSGLHYRRYPFFAALRERAGP**VTWVMMIACVVVFIAMQI**LGDQEVMLWLAWPFDPTLKFEFWRYFTHALMHFS**LMHILFNLLWWWYLGGAVEKRL**G**SGKLIVITLISALLSGYVQQKFTG**PWFGG**LSGVVYALMGYVWLRGERDPQSGI**YL**QRGLIIFALIWIVAGWF**DLFGMSM**ANGAHIAGLAVGLAMAFVDS**LNARKRKSGSGGQYFMMGDNRDNSSSYPYDVPDYAFSTPVWISQAQGIRAGPGSSDKQEGEWPTGLRLSRIGGIH **321**

MLMITSFANPRVAQAFVDYMATQGVILTIQQHNQSDVWLADESQAERVRADVARFLENPADPRYLAASWQAGHTGSGLHYRRYPFFAALRERAGP**VTWVMMIACVVVFIAMQI**LGDQEVMLWLAWPFDPTLKFEFWRYFTHALMHFS**LMHILFNLLWWWYLGGAVEKRL**G**SGKLIVITLISALLSGYVQQKFTG**PWFGG**LSGVVYALMGYVWLRGERDPQSGI**YL**QRGLIIFALIWIVAGWF**DLFGMSM**ANGAHIAGLAVGLAMAFVDS**LNARKRKSGSGGQYFMMGDSSYPYDVPDYAFSTPVWISQAQGIRAGPGSSDKQEGEWPTGLRLSRIGGIH **316**

MLMITSFANPRVAQAFVDYMATQGVILTIQQHNQSDVWLADESQAERVRADVARFLENPADPRYLAASWQAGHTGSGLHYRRYPFFAALRERAGP**VTWVMMIACVVVFIAMQI**LGDQEVMLWLAWPFDPTLKFEFWRYFTHALMHFS**LMHILFNLLWWWYLGGAVEKRL**G**SGKLIVITLISALLSGYVQQKFTG**PWFGG**LSGVVYALMGYVWLRGERDPQSGI**YL**QRGLIIFALIWIVAGWF**DLFGMSM**ANGAHIAGLAVGLAMAFVDS**LNARKRKSGSGGQYSSYPYDVPDYAFSTPVWISQAQGIRAGPGSSDKQEGEWPTGLRLSRIGGIH **311**

MLMITSFANPRVAQAFVDYMATQGVILTIQQHNQSDVWLADESQAERVRADVARFLENPADPRYLAASWQAGHTGSGLHYRRYPFFAALRERAGP**VTWVMMIACVVVFIAMQI**LGDQEVMLWLAWPFDPTLKFEFWRYFTHALMHFS**LMHILFNLLWWWYLGGAVEKRL**G**SGKLIVITLISALLSGYVQQKFTG**PWFGG**LSGVVYALMGYVWLRGERDPQSGI**YL**QRGLIIFALIWIVAGWF**DLFGMSM**ANGAHIAGLAVGLAMAFVDS**LNARKRKSGSGYPYDVPDYAFSTPVWISQAQGIRAGPGSSDKQEGEWPTGLRLSRIGGIH **306**

MLMITSFANPRVAQAFVDYMATQGVILTIQQHNQSDVWLADESQAERVRADVARFLENPADPRYLAASWQAGHTGSGLHYRRYPFFAALRERAGP**VTWVMMIACVVVFIAMQI**LGDQEVMLWLAWPFDPTLKFEFWRYFTHALMHFS**LMHILFNLLWWWYLGGAVEKRL**G**SGKLIVITLISALLSGYVQQKFTG**PWFGG**LSGVVYALMGYVWLRGERDPQSGI**YL**QRGLIIFALIWIVAGWF**DLFGMSM**ANGAHIAGLAVGLAMAFVDS**LNSGSGYPYDVPDYAFSTPVWISQAQGIRAGPGSSDKQEGEWPTGLRLSRIGGIH  **301**

MLMITSFANPRVAQAFVDYMATQGVILTIQQHNQSDVWLADESQAERVRADVARFLENPADPRYLAASWQAGHTGSGLHYRRYPFFAALRERAGP**VTWVMMIACVVVFIAMQI**LGDQEVMLWLAWPFDPTLKFEFWRYFTHALMHFS**LMHILFNLLWWWYLGGAVEKRL**G**SGKLIVITLISALLSGYVQQKFTG**PWFGG**LSGVVYALMGYVWLRGERDPQSGI**YL**QRGLIIFALIWIVAGWF**DLFGMSM**ANGAHIAGLAVGLAMAF**SGSGYPYDVPDYAFSTPVWISQAQGIRAGPGSSDKQEGEWPTGLRLSRIGGIH  **296**

MLMITSFANPRVAQAFVDYMATQGVILTIQQHNQSDVWLADESQAERVRADVARFLENPADPRYLAASWQAGHTGSGLHYRRYPFFAALRERAGP**VTWVMMIACVVVFIAMQI**LGDQEVMLWLAWPFDPTLKFEFWRYFTHALMHFS**LMHILFNLLWWWYLGGAVEKRL**G**SGKLIVITLISALLSGYVQQKFTG**PWFGG**LSGVVYALMGYVWLRGERDPQSGI**YL**QRGLIIFALIWIVAGWF**DLFGMSM**ANGAHIAGLAVG**SGSGYPYDVPDYAFSTPVWISQAQGIRAGPGSSDKQEGEWPTGLRLSRIGGIH **291**

MLMITSFANPRVAQAFVDYMATQGVILTIQQHNQSDVWLADESQAERVRADVARFLENPADPRYLAASWQAGHTGSGLHYRRYPFFAALRERAGP**VTWVMMIACVVVFIAMQI**LGDQEVMLWLAWPFDPTLKFEFWRYFTHALMHFS**LMHILFNLLWWWYLGGAVEKRL**G**SGKLIVITLISALLSGYVQQKFTG**PWFGG**LSGVVYALMGYVWLRGERDPQSGI**YL**QRGLIIFALIWIVAGWF**DLFGMSM**ANGAHIA**SGSGYPYDVPDYAFSTPVWISQAQGIRAGPGSSDKQEGEWPTGLRLSRIGGIH **286**

MLMITSFANPRVAQAFVDYMATQGVILTIQQHNQSDVWLADESQAERVRADVARFLENPADPRYLAASWQAGHTGSGLHYRRYPFFAALRERAGP**VTWVMMIACVVVFIAMQI**LGDQEVMLWLAWPFDPTLKFEFWRYFTHALMHFS**LMHILFNLLWWWYLGGAVEKRL**G**SGKLIVITLISALLSGYVQQKFTG**PWFGG**LSGVVYALMGYVWLRGERDPQSGI**YL**QRGLIIFALIWIVAGWF**DLFGMSM**AN**SGSGYPYDVPDYAFSTPVWISQAQGIRAGPGSSDKQEGEWPTGLRLSRIGGIH **281**

MLMITSFANPRVAQAFVDYMATQGVILTIQQHNQSDVWLADESQAERVRADVARFLENPADPRYLAASWQAGHTGSGLHYRRYPFFAALRERAGP**VTWVMMIACVVVFIAMQI**LGDQEVMLWLAWPFDPTLKFEFWRYFTHALMHFS**LMHILFNLLWWWYLGGAVEKRL**G**SGKLIVITLISALLSGYVQQKFTG**PWFGG**LSGVVYALMGYVWLRGERDPQSGI**YL**QRGLIIFALIWIVAGWF**DLFGSGSGYPYDVPDYAFSTPVWISQAQGIRAGPGSSDKQEGEWPTGLRLSRIGGIH **276**

MLMITSFANPRVAQAFVDYMATQGVILTIQQHNQSDVWLADESQAERVRADVARFLENPADPRYLAASWQAGHTGSGLHYRRYPFFAALRERAGP**VTWVMMIACVVVFIAMQI**LGDQEVMLWLAWPFDPTLKFEFWRYFTHALMHFS**LMHILFNLLWWWYLGGAVEKRL**G**SGKLIVITLISALLSGYVQQKFTG**PWFGG**LSGVVYALMGYVWLRGERDPQSGI**YL**QRGLIIFALIWIVAGW**SGSGYPYDVPDYAFSTPVWISQAQGIRAGPGSSDKQEGEWPTGLRLSRIGGIH **271**

MLMITSFANPRVAQAFVDYMATQGVILTIQQHNQSDVWLADESQAERVRADVARFLENPADPRYLAASWQAGHTGSGLHYRRYPFFAALRERAGP**VTWVMMIACVVVFIAMQI**LGDQEVMLWLAWPFDPTLKFEFWRYFTHALMHFS**LMHILFNLLWWWYLGGAVEKRL**G**SGKLIVITLISALLSGYVQQKFTG**PWFGG**LSGVVYALMGYVWLRGERDPQSGI**YL**QRGLIIFALIW**SGSGYPYDVPDYAFSTPVWISQAQGIRAGPGSSDKQEGEWPTGLRLSRIGGIH **266**

MLMITSFANPRVAQAFVDYMATQGVILTIQQHNQSDVWLADESQAERVRADVARFLENPADPRYLAASWQAGHTGSGLHYRRYPFFAALRERAGP**VTWVMMIACVVVFIAMQI**LGDQEVMLWLAWPFDPTLKFEFWRYFTHALMHFS**LMHILFNLLWWWYLGGAVEKRL**G**SGKLIVITLISALLSGYVQQKFTG**PWFGG**LSGVVYALMGYVWLRGERDPQSGI**YL**QRGLII**SGSGYPYDVPDYAFSTPVWISQAQGIRAGPGSSDKQEGEWPTGLRLSRIGGIH **261**

MLMITSFANPRVAQAFVDYMATQGVILTIQQHNQSDVWLADESQAERVRADVARFLENPADPRYLAASWQAGHTGSGLHYRRYPFFAALRERAGP**VTWVMMIACVVVFIAMQI**LGDQEVMLWLAWPFDPTLKFEFWRYFTHALMHFS**LMHILFNLLWWWYLGGAVEKRL**G**SGKLIVITLISALLSGYVQQKFTG**PWFGG**LSGVVYALMGYVWLRGERDPQSGI**Y**LQ**SGSGYPYDVPDYAFSTPVWISQAQGIRAGPGSSDKQEGEWPTGLRLSRIGGIH  **256**

MLMITSFANPRVAQAFVDYMATQGVILTIQQHNQSDVWLADESQAERVRADVARFLENPADPRYLAASWQAGHTGSGLHYRRYPFFAALRERAGP**VTWVMMIACVVVFIAMQI**LGDQEVMLWLAWPFDPTLKFEFWRYFTHALMHFS**LMHILFNLLWWWYLGGAVEKRL**G**SGKLIVITLISALLSGYVQQKFTG**PWFGG**LSGVVYALMGYVWLRGERDPQS**SGSGYPYDVPDYAFSTPVWISQAQGIRAGPGSSDKQEGEWPTGLRLSRIGGIH **251**

MLMITSFANPRVAQAFVDYMATQGVILTIQQHNQSDVWLADESQAERVRADVARFLENPADPRYLAASWQAGHTGSGLHYRRYPFFAALRERAGP**VTWVMMIACVVVFIAMQI**LGDQEVMLWLAWPFDPTLKFEFWRYFTHALMHFS**LMHILFNLLWWWYLGGAVEKRL**G**SGKLIVITLISALLSGYVQQKFTG**PWFGG**LSGVVYALMGYVWLRGE**SGSGYPYDVPDYAFSTPVWISQAQGIRAGPGSSDKQEGEWPTGLRLSRIGGIH **246**

MLMITSFANPRVAQAFVDYMATQGVILTIQQHNQSDVWLADESQAERVRADVARFLENPADPRYLAASWQAGHTGSGLHYRRYPFFAALRERAGP**VTWVMMIACVVVFIAMQI**LGDQEVMLWLAWPFDPTLKFEFWRYFTHALMHFS**LMHILFNLLWWWYLGGAVEKRL**G**SGKLIVITLISALLSGYVQQKFTG**PWFGG**LSGVVYALMGYV**SGSGYPYDVPDYAFSTPVWISQAQGIRAGPGSSDKQEGEWPTGLRLSRIGGIH **241**

MLMITSFANPRVAQAFVDYMATQGVILTIQQHNQSDVWLADESQAERVRADVARFLENPADPRYLAASWQAGHTGSGLHYRRYPFFAALRERAGP**VTWVMMIACVVVFIAMQI**LGDQEVMLWLAWPFDPTLKFEFWRYFTHALMHFS**LMHILFNLLWWWYLGGAVEKRL**G**SGKLIVITLISALLSGYVQQKFTG**PWFGG**LSGVVYA**SGSGYPYDVPDYAFSTPVWISQAQGIRAGPGSSDKQEGEWPTGLRLSRIGGIH  **236**

MLMITSFANPRVAQAFVDYMATQGVILTIQQHNQSDVWLADESQAERVRADVARFLENPADPRYLAASWQAGHTGSGLHYRRYPFFAALRERAGP**VTWVMMIACVVVFIAMQI**LGDQEVMLWLAWPFDPTLKFEFWRYFTHALMHFS**LMHILFNLLWWWYLGGAVEKRL**G**SGKLIVITLISALLSGYVQQKFTG**PWFGG**LS**SGSGYPYDVPDYAFSTPVWISQAQGIRAGPGSSDKQEGEWPTGLRLSRIGGIH **231**

MLMITSFANPRVAQAFVDYMATQGVILTIQQHNQSDVWLADESQAERVRADVARFLENPADPRYLAASWQAGHTGSGLHYRRYPFFAALRERAGP**VTWVMMIACVVVFIAMQI**LGDQEVMLWLAWPFDPTLKFEFWRYFTHALMHFS**LMHILFNLLWWWYLGGAVEKRL**G**SGKLIVITLISALLSGYVQQKFTG**PWSGSGYPYDVPDYAFSTPVWISQAQGIRAGPGSSDKQEGEWPTGLRLSRIGGIH **226**

MLMITSFANPRVAQAFVDYMATQGVILTIQQHNQSDVWLADESQAERVRADVARFLENPADPRYLAASWQAGHTGSGLHYRRYPFFAALRERAGP**VTWVMMIACVVVFIAMQI**LGDQEVMLWLAWPFDPTLKFEFWRYFTHALMHFS**LMHILFNLLWWWYLGGAVEKRL**G**SGKLIVITLISALLSGYVQQK**SGSGYPYDVPDYAFSTPVWISQAQGIRAGPGSSDKQEGEWPTGLRLSRIGGIH **221**

MLMITSFANPRVAQAFVDYMATQGVILTIQQHNQSDVWLADESQAERVRADVARFLENPADPRYLAASWQAGHTGSGLHYRRYPFFAALRERAGP**VTWVMMIACVVVFIAMQI**LGDQEVMLWLAWPFDPTLKFEFWRYFTHALMHFS**LMHILFNLLWWWYLGGAVEKRL**G**SGKLIVITLISALLSG**SGSGYPYDVPDYAFSTPVWISQAQGIRAGPGSSDKQEGEWPTGLRLSRIGGIH **216**

MLMITSFANPRVAQAFVDYMATQGVILTIQQHNQSDVWLADESQAERVRADVARFLENPADPRYLAASWQAGHTGSGLHYRRYPFFAALRERAGP**VTWVMMIACVVVFIAMQI**LGDQEVMLWLAWPFDPTLKFEFWRYFTHALMHFS**LMHILFNLLWWWYLGGAVEKRL**G**SGKLIVITLIS**SGSGYPYDVPDYAFSTPVWISQAQGIRAGPGSSDKQEGEWPTGLRLSRIGGIH **211**

MLMITSFANPRVAQAFVDYMATQGVILTIQQHNQSDVWLADESQAERVRADVARFLENPADPRYLAASWQAGHTGSGLHYRRYPFFAALRERAGP**VTWVMMIACVVVFIAMQI**LGDQEVMLWLAWPFDPTLKFEFWRYFTHALMHFS**LMHILFNLLWWWYLGGAVEKRL**G**SGKLIV**SGSGYPYDVPDYAFSTPVWISQAQGIRAGPGSSDKQEGEWPTGLRLSRIGGIH **206**

MLMITSFANPRVAQAFVDYMATQGVILTIQQHNQSDVWLADESQAERVRADVARFLENPADPRYLAASWQAGHTGSGLHYRRYPFFAALRERAGP**VTWVMMIACVVVFIAMQI**LGDQEVMLWLAWPFDPTLKFEFWRYFTHALMHFS**LMHILFNLLWWWYLGGAVEKRL**G**S**SGSGYPYDVPDYAFSTPVWISQAQGIRAGPGSSDKQEGEWPTGLRLSRIGGIH **201**

MLMITSFANPRVAQAFVDYMATQGVILTIQQHNQSDVWLADESQAERVRADVARFLENPADPRYLAASWQAGHTGSGLHYRRYPFFAALRERAGP**VTWVMMIACVVVFIAMQI**LGDQEVMLWLAWPFDPTLKFEFWRYFTHALMHFS**LMHILFNLLWWWYLGGAVE**SGSGYPYDVPDYAFSTPVWISQAQGIRAGPGSSDKQEGEWPTGLRLSRIGGIH **196**

MLMITSFANPRVAQAFVDYMATQGVILTIQQHNQSDVWLADESQAERVRADVARFLENPADPRYLAASWQAGHTGSGLHYRRYPFFAALRERAGP**VTWVMMIACVVVFIAMQI**LGDQEVMLWLAWPFDPTLKFEFWRYFTHALMHFS**LMHILFNLLWWWYL**SGSGYPYDVPDYAFSTPVWISQAQGIRAGPGSSDKQEGEWPTGLRLSRIGGIH **191**

MLMITSFANPRVAQAFVDYMATQGVILTIQQHNQSDVWLADESQAERVRADVARFLENPADPRYLAASWQAGHTGSGLHYRRYPFFAALRERAGP**VTWVMMIACVVVFIAMQI**LGDQEVMLWLAWPFDPTLKFEFWRYFTHALMHFS**LMHILFNLL**SGSGYPYDVPDYAFSTPVWISQAQGIRAGPGSSDKQEGEWPTGLRLSRIGGIH **186**

MLMITSFANPRVAQAFVDYMATQGVILTIQQHNQSDVWLADESQAERVRADVARFLENPADPRYLAASWQAGHTGSGLHYRRYPFFAALRERAGP**VTWVMMIACVVVFIAMQI**LGDQEVMLWLAWPFDPTLKFEFWRYFTHALMHFS**LMHI**SGSGYPYDVPDYAFSTPVWISQAQGIRAGPGSSDKQEGEWPTGLRLSRIGGIH **181**

MLMITSFANPRVAQAFVDYMATQGVILTIQQHNQSDVWLADESQAERVRADVARFLENPADPRYLAASWQAGHTGSGLHYRRYPFFAALRERAGP**VTWVMMIACVVVFIAMQI**LGDQEVMLWLAWPFDPTLKFEFWRYFTHALMHFSGSGYPYDVPDYAFSTPVWISQAQGIRAGPGSSDKQEGEWPTGLRLSRIGGIH **176**

MLMITSFANPRVAQAFVDYMATQGVILTIQQHNQSDVWLADESQAERVRADVARFLENPADPRYLAASWQAGHTGSGLHYRRYPFFAALRERAGP**VTWVMMIACVVVFIAMQI**LGDQEVMLWLAWPFDPTLKFEFWRYFTHSGSGYPYDVPDYAFSTPVWISQAQGIRAGPGSSDKQEGEWPTGLRLSRIGGIH **171**

MLMITSFANPRVAQAFVDYMATQGVILTIQQHNQSDVWLADESQAERVRADVARFLENPADPRYLAASWQAGHTGSGLHYRRYPFFAALRERAGP**VTWVMMIACVVVFIAMQI**LGDQEVMLWLAWPFDPTLKFEFWSGSGYPYDVPDYAFSTPVWISQAQGIRAGPGSSDKQEGEWPTGLRLSRIGGIH **166**

MLMITSFANPRVAQAFVDYMATQGVILTIQQHNQSDVWLADESQAERVRADVARFLENPADPRYLAASWQAGHTGSGLHYRRYPFFAALRERAGP**VTWVMMIACVVVFIAMQI**LGDQEVMLWLAWPFDPTLSGSGYPYDVPDYAFSTPVWISQAQGIRAGPGSSDKQEGEWPTGLRLSRIGGIH  **161**

MLMITSFANPRVAQAFVDYMATQGVILTIQQHNQSDVWLADESQAERVRADVARFLENPADPRYLAASWQAGHTGSGLHYRRYPFFAALRERAGP**VTWVMMIACVVVFIAMQI**LGDQEVMLWLAWPSGSGYPYDVPDYAFSTPVWISQAQGIRAGPGSSDKQEGEWPTGLRLSRIGGIH **156**

MLMITSFANPRVAQAFVDYMATQGVILTIQQHNQSDVWLADESQAERVRADVARFLENPADPRYLAASWQAGHTGSGLHYRRYPFFAALRERAGP**VTWVMMIACVVVFIAMQI**LGDQEVMLSGSGYPYDVPDYAFSTPVWISQAQGIRAGPGSSDKQEGEWPTGLRLSRIGGIH **151**

MLMITSFANPRVAQAFVDYMATQGVILTIQQHNQSDVWLADESQAERVRADVARFLENPADPRYLAASWQAGHTGSGLHYRRYPFFAALRERAGP**VTWVMMIACVVVFIAMQI**LGDSGSGYPYDVPDYAFSTPVWISQAQGIRAGPGSSDKQEGEWPTGLRLSRIGGIH **146**

MLMITSFANPRVAQAFVDYMATQGVILTIQQHNQSDVWLADESQAERVRADVARFLENPADPRYLAASWQAGHTGSGLHYRRYPFFAALRERAGP**VTWVMMIACVVVFIAM**SGSGYPYDVPDYAFSTPVWISQAQGIRAGPGSSDKQEGEWPTGLRLSRIGGIH **141**

MLMITSFANPRVAQAFVDYMATQGVILTIQQHNQSDVWLADESQAERVRADVARFLENPADPRYLAASWQAGHTGSGLHYRRYPFFAALRERAGP**VTWVMMIACVV**SGSGYPYDVPDYAFSTPVWISQAQGIRAGPGSSDKQEGEWPTGLRLSRIGGIH **136**

MLMITSFANPRVAQAFVDYMATQGVILTIQQHNQSDVWLADESQAERVRADVARFLENPADPRYLAASWQAGHTGSGLHYRRYPFFAALRERAGP**VTWVMM**SGSGYPYDVPDYAFSTPVWISQAQGIRAGPGSSDKQEGEWPTGLRLSRIGGIH **131**

MLMITSFANPRVAQAFVDYMATQGVILTIQQHNQSDVWLADESQAERVRADVARFLENPADPRYLAASWQAGHTGSGLHYRRYPFFAALRERAGP**V**SGSGYPYDVPDYAFSTPVWISQAQGIRAGPGSSDKQEGEWPTGLRLSRIGGIH  **126**

MLMITSFANPRVAQAFVDYMATQGVILTIQQHNQSDVWLADESQAERVRADVARFLENPADPRYLAASWQAGHTGSGLHYRRYPFFAALRESGSGYPYDVPDYAFSTPVWISQAQGIRAGPGSSDKQEGEWPTGLRLSRIGGIH  **121**

MLMITSFANPRVAQAFVDYMATQGVILTIQQHNQSDVWLADESQAERVRADVARFLENPADPRYLAASWQAGHTGSGLHYRRYPFFSGSGYPYDVPDYAFSTPVWISQAQGIRAGPGSSDKQEGEWPTGLRLSRIGGIH **116**

MLMITSFANPRVAQAFVDYMATQGVILTIQQHNQSDVWLADESQAERVRADVARFLENPADPRYLAASWQAGHTGSGLHYRSGSGYPYDVPDYAFSTPVWISQAQGIRAGPGSSDKQEGEWPTGLRLSRIGGIH  **111**

MLMITSFANPRVAQAFVDYMATQGVILTIQQHNQSDVWLADESQAERVRADVARFLENPADPRYLAASWQAGHTGSSGSGYPYDVPDYAFSTPVWISQAQGIRAGPGSSDKQEGEWPTGLRLSRIGGIH **106**

MLMITSFANPRVAQAFVDYMATQGVILTIQQHNQSDVWLADESQAERVRADVARFLENPADPRYLAASWQASGSGYPYDVPDYAFSTPVWISQAQGIRAGPGSSDKQEGEWPTGLRLSRIGGIH **101**

MLMITSFANPRVAQAFVDYMATQGVILTIQQHNQSDVWLADESQAERVRADVARFLENPADPRYLASGSGYPYDVPDYAFSTPVWISQAQGIRAGPGSSDKQEGEWPTGLRLSRIGGIH **96**

MLMITSFANPRVAQAFVDYMATQGVILTIQQHNQSDVWLADESQAERVRADVARFLENPADSGSGYPYDVPDYAFSTPVWISQAQGIRAGPGSSDKQEGEWPTGLRLSRIGGIH **91**

MLMITSFANPRVAQAFVDYMATQGVILTIQQHNQSDVWLADESQAERVRADVARFLSGSGYPYDVPDYAFSTPVWISQAQGIRAGPGSSDKQEGEWPTGLRLSRIGGIH **86**

MLMITSFANPRVAQAFVDYMATQGVILTIQQHNQSDVWLADESQAERVRADSGSGYPYDVPDYAFSTPVWISQAQGIRAGPGSSDKQEGEWPTGLRLSRIGGIH **81**

MLMITSFANPRVAQAFVDYMATQGVILTIQQHNQSDVWLADESQAESGSGYPYDVPDYAFSTPVWISQAQGIRAGPGSSDKQEGEWPTGLRLSRIGGIH **76**

MLMITSFANPRVAQAFVDYMATQGVILTIQQHNQSDVWLADSGSGYPYDVPDYAFSTPVWISQAQGIRAGPGSSDKQEGEWPTGLRLSRIGGIH **71**

MLMITSFANPRVAQAFVDYMATQGVILTIQQHNQSDSGSGYPYDVPDYAFSTPVWISQAQGIRAGPGSSDKQEGEWPTGLRLSRIGGIH **66**

MLMITSFANPRVAQAFVDYMATQGVILTIQQSGSGYPYDVPDYAFSTPVWISQAQGIRAGPGSSDKQEGEWPTGLRLSRIGGIH **61**

MLMITSFANPRVAQAFVDYMATQGVISGSGYPYDVPDYAFSTPVWISQAQGIRAGPGSSDKQEGEWPTGLRLSRIGGIH **56**

MLMITSFANPRVAQAFVDYMASGSGYPYDVPDYAFSTPVWISQAQGIRAGPGSSDKQEGEWPTGLRLSRIGGIH **51**

MLMITSFANPRVAQAFSGSGYPYDVPDYAFSTPVWISQAQGIRAGPGSSDKQEGEWPTGLRLSRIGGIH **46**

LepB TM1 GlpG SecM(*Ec*) (N=131-224)

MANMFALILVIATLVTGILWCVDKFFFAPKRRERQAAAQAAAGDSLDKATLKKVAPKPDPRYLAASWQAGHTGSGLHYRRYPFFAALRERAGP**VTWVMMIACVVVFIAMQI**LGDQEVMLWLAWPFDPTLKFEFWRYFTHALMHFS**LMHILFNLLWWWYLGGAVEKRL**G**SGKLIVITLISALLSGYVQQKFTG**SGSGYPYDVPDYAFSTPVWISQAQGIRAGPGSSDKQEGEWPTGLRLSRIGGIH 224

MANMFALILVIATLVTGILWCVDKFFFAPKRRERQAAAQAAAGDSLDKATLKKVAPKPDPRYLAASWQAGHTGSGLHYRRYPFFAALRERAGP**VTWVMMIACVVVFIAMQI**LGDQEVMLWLAWPFDPTLKFEFWRYFTHALMHFS**LMHILFNLLWWWYLGGAVEKRL**G**SGKLIVITLISALLSGYVQQKFT**SGSGYPYDVPDYAFSTPVWISQAQGIRAGPGSSDKQEGEWPTGLRLSRIGGIH 223

MANMFALILVIATLVTGILWCVDKFFFAPKRRERQAAAQAAAGDSLDKATLKKVAPKPDPRYLAASWQAGHTGSGLHYRRYPFFAALRERAGP**VTWVMMIACVVVFIAMQI**LGDQEVMLWLAWPFDPTLKFEFWRYFTHALMHFS**LMHILFNLLWWWYLGGAVEKRL**G**SGKLIVITLISALLSGYVQQKF**SGSGYPYDVPDYAFSTPVWISQAQGIRAGPGSSDKQEGEWPTGLRLSRIGGIH 222

MANMFALILVIATLVTGILWCVDKFFFAPKRRERQAAAQAAAGDSLDKATLKKVAPKPDPRYLAASWQAGHTGSGLHYRRYPFFAALRERAGP**VTWVMMIACVVVFIAMQI**LGDQEVMLWLAWPFDPTLKFEFWRYFTHALMHFS**LMHILFNLLWWWYLGGAVEKRL**G**SGKLIVITLISALLSGYVQQK**SGSGYPYDVPDYAFSTPVWISQAQGIRAGPGSSDKQEGEWPTGLRLSRIGGIH 221

MANMFALILVIATLVTGILWCVDKFFFAPKRRERQAAAQAAAGDSLDKATLKKVAPKPDPRYLAASWQAGHTGSGLHYRRYPFFAALRERAGP**VTWVMMIACVVVFIAMQI**LGDQEVMLWLAWPFDPTLKFEFWRYFTHALMHFS**LMHILFNLLWWWYLGGAVEKRL**G**SGKLIVITLISALLSGYVQQ**SGSGYPYDVPDYAFSTPVWISQAQGIRAGPGSSDKQEGEWPTGLRLSRIGGIH 220

MANMFALILVIATLVTGILWCVDKFFFAPKRRERQAAAQAAAGDSLDKATLKKVAPKPDPRYLAASWQAGHTGSGLHYRRYPFFAALRERAGP**VTWVMMIACVVVFIAMQI**LGDQEVMLWLAWPFDPTLKFEFWRYFTHALMHFS**LMHILFNLLWWWYLGGAVEKRL**G**SGKLIVITLISALLSGYVQ**SGSGYPYDVPDYAFSTPVWISQAQGIRAGPGSSDKQEGEWPTGLRLSRIGGIH 219

MANMFALILVIATLVTGILWCVDKFFFAPKRRERQAAAQAAAGDSLDKATLKKVAPKPDPRYLAASWQAGHTGSGLHYRRYPFFAALRERAGP**VTWVMMIACVVVFIAMQI**LGDQEVMLWLAWPFDPTLKFEFWRYFTHALMHFS**LMHILFNLLWWWYLGGAVEKRL**G**SGKLIVITLISALLSGYV**SGSGYPYDVPDYAFSTPVWISQAQGIRAGPGSSDKQEGEWPTGLRLSRIGGIH 218

MANMFALILVIATLVTGILWCVDKFFFAPKRRERQAAAQAAAGDSLDKATLKKVAPKPDPRYLAASWQAGHTGSGLHYRRYPFFAALRERAGP**VTWVMMIACVVVFIAMQI**LGDQEVMLWLAWPFDPTLKFEFWRYFTHALMHFS**LMHILFNLLWWWYLGGAVEKRL**G**SGKLIVITLISALLSGY**SGSGYPYDVPDYAFSTPVWISQAQGIRAGPGSSDKQEGEWPTGLRLSRIGGIH 217

MANMFALILVIATLVTGILWCVDKFFFAPKRRERQAAAQAAAGDSLDKATLKKVAPKPDPRYLAASWQAGHTGSGLHYRRYPFFAALRERAGP**VTWVMMIACVVVFIAMQI**LGDQEVMLWLAWPFDPTLKFEFWRYFTHALMHFS**LMHILFNLLWWWYLGGAVEKRL**G**SGKLIVITLISALLSG**SGSGYPYDVPDYAFSTPVWISQAQGIRAGPGSSDKQEGEWPTGLRLSRIGGIH 216

MANMFALILVIATLVTGILWCVDKFFFAPKRRERQAAAQAAAGDSLDKATLKKVAPKPDPRYLAASWQAGHTGSGLHYRRYPFFAALRERAGP**VTWVMMIACVVVFIAMQI**LGDQEVMLWLAWPFDPTLKFEFWRYFTHALMHFS**LMHILFNLLWWWYLGGAVEKRL**G**SGKLIVITLISALLS**SGSGYPYDVPDYAFSTPVWISQAQGIRAGPGSSDKQEGEWPTGLRLSRIGGIH 215

MANMFALILVIATLVTGILWCVDKFFFAPKRRERQAAAQAAAGDSLDKATLKKVAPKPDPRYLAASWQAGHTGSGLHYRRYPFFAALRERAGP**VTWVMMIACVVVFIAMQI**LGDQEVMLWLAWPFDPTLKFEFWRYFTHALMHFS**LMHILFNLLWWWYLGGAVEKRL**G**SGKLIVITLISALL**SGSGYPYDVPDYAFSTPVWISQAQGIRAGPGSSDKQEGEWPTGLRLSRIGGIH 214

MANMFALILVIATLVTGILWCVDKFFFAPKRRERQAAAQAAAGDSLDKATLKKVAPKPDPRYLAASWQAGHTGSGLHYRRYPFFAALRERAGP**VTWVMMIACVVVFIAMQI**LGDQEVMLWLAWPFDPTLKFEFWRYFTHALMHFS**LMHILFNLLWWWYLGGAVEKRL**G**SGKLIVITLISAL**SGSGYPYDVPDYAFSTPVWISQAQGIRAGPGSSDKQEGEWPTGLRLSRIGGIH 213

MANMFALILVIATLVTGILWCVDKFFFAPKRRERQAAAQAAAGDSLDKATLKKVAPKPDPRYLAASWQAGHTGSGLHYRRYPFFAALRERAGP**VTWVMMIACVVVFIAMQI**LGDQEVMLWLAWPFDPTLKFEFWRYFTHALMHFS**LMHILFNLLWWWYLGGAVEKRL**G**SGKLIVITLISA**SGSGYPYDVPDYAFSTPVWISQAQGIRAGPGSSDKQEGEWPTGLRLSRIGGIH 212

MANMFALILVIATLVTGILWCVDKFFFAPKRRERQAAAQAAAGDSLDKATLKKVAPKPDPRYLAASWQAGHTGSGLHYRRYPFFAALRERAGP**VTWVMMIACVVVFIAMQI**LGDQEVMLWLAWPFDPTLKFEFWRYFTHALMHFS**LMHILFNLLWWWYLGGAVEKRL**G**SGKLIVITLIS**SGSGYPYDVPDYAFSTPVWISQAQGIRAGPGSSDKQEGEWPTGLRLSRIGGIH 211

MANMFALILVIATLVTGILWCVDKFFFAPKRRERQAAAQAAAGDSLDKATLKKVAPKPDPRYLAASWQAGHTGSGLHYRRYPFFAALRERAGP**VTWVMMIACVVVFIAMQI**LGDQEVMLWLAWPFDPTLKFEFWRYFTHALMHFS**LMHILFNLLWWWYLGGAVEKRL**G**SGKLIVITLI**SGSGYPYDVPDYAFSTPVWISQAQGIRAGPGSSDKQEGEWPTGLRLSRIGGIH 210

MANMFALILVIATLVTGILWCVDKFFFAPKRRERQAAAQAAAGDSLDKATLKKVAPKPDPRYLAASWQAGHTGSGLHYRRYPFFAALRERAGP**VTWVMMIACVVVFIAMQI**LGDQEVMLWLAWPFDPTLKFEFWRYFTHALMHFS**LMHILFNLLWWWYLGGAVEKRL**G**SGKLIVITL**SGSGYPYDVPDYAFSTPVWISQAQGIRAGPGSSDKQEGEWPTGLRLSRIGGIH 209

MANMFALILVIATLVTGILWCVDKFFFAPKRRERQAAAQAAAGDSLDKATLKKVAPKPDPRYLAASWQAGHTGSGLHYRRYPFFAALRERAGP**VTWVMMIACVVVFIAMQI**LGDQEVMLWLAWPFDPTLKFEFWRYFTHALMHFS**LMHILFNLLWWWYLGGAVEKRL**G**SGKLIVIT**SGSGYPYDVPDYAFSTPVWISQAQGIRAGPGSSDKQEGEWPTGLRLSRIGGIH 208

MANMFALILVIATLVTGILWCVDKFFFAPKRRERQAAAQAAAGDSLDKATLKKVAPKPDPRYLAASWQAGHTGSGLHYRRYPFFAALRERAGP**VTWVMMIACVVVFIAMQI**LGDQEVMLWLAWPFDPTLKFEFWRYFTHALMHFS**LMHILFNLLWWWYLGGAVEKRL**G**SGKLIVI**SGSGYPYDVPDYAFSTPVWISQAQGIRAGPGSSDKQEGEWPTGLRLSRIGGIH 207

MANMFALILVIATLVTGILWCVDKFFFAPKRRERQAAAQAAAGDSLDKATLKKVAPKPDPRYLAASWQAGHTGSGLHYRRYPFFAALRERAGP**VTWVMMIACVVVFIAMQI**LGDQEVMLWLAWPFDPTLKFEFWRYFTHALMHFS**LMHILFNLLWWWYLGGAVEKRL**G**SGKLIV**SGSGYPYDVPDYAFSTPVWISQAQGIRAGPGSSDKQEGEWPTGLRLSRIGGIH 206

MANMFALILVIATLVTGILWCVDKFFFAPKRRERQAAAQAAAGDSLDKATLKKVAPKPDPRYLAASWQAGHTGSGLHYRRYPFFAALRERAGP**VTWVMMIACVVVFIAMQI**LGDQEVMLWLAWPFDPTLKFEFWRYFTHALMHFS**LMHILFNLLWWWYLGGAVEKRL**G**SGKLI**SGSGYPYDVPDYAFSTPVWISQAQGIRAGPGSSDKQEGEWPTGLRLSRIGGIH 205

MANMFALILVIATLVTGILWCVDKFFFAPKRRERQAAAQAAAGDSLDKATLKKVAPKPDPRYLAASWQAGHTGSGLHYRRYPFFAALRERAGP**VTWVMMIACVVVFIAMQI**LGDQEVMLWLAWPFDPTLKFEFWRYFTHALMHFS**LMHILFNLLWWWYLGGAVEKRL**G**SGKL**SGSGYPYDVPDYAFSTPVWISQAQGIRAGPGSSDKQEGEWPTGLRLSRIGGIH 204

MANMFALILVIATLVTGILWCVDKFFFAPKRRERQAAAQAAAGDSLDKATLKKVAPKPDPRYLAASWQAGHTGSGLHYRRYPFFAALRERAGP**VTWVMMIACVVVFIAMQI**LGDQEVMLWLAWPFDPTLKFEFWRYFTHALMHFS**LMHILFNLLWWWYLGGAVEKRL**G**SGK**SGSGYPYDVPDYAFSTPVWISQAQGIRAGPGSSDKQEGEWPTGLRLSRIGGIH 203

MANMFALILVIATLVTGILWCVDKFFFAPKRRERQAAAQAAAGDSLDKATLKKVAPKPDPRYLAASWQAGHTGSGLHYRRYPFFAALRERAGP**VTWVMMIACVVVFIAMQI**LGDQEVMLWLAWPFDPTLKFEFWRYFTHALMHFS**LMHILFNLLWWWYLGGAVEKRL**G**SG**SGSGYPYDVPDYAFSTPVWISQAQGIRAGPGSSDKQEGEWPTGLRLSRIGGIH 202

MANMFALILVIATLVTGILWCVDKFFFAPKRRERQAAAQAAAGDSLDKATLKKVAPKPDPRYLAASWQAGHTGSGLHYRRYPFFAALRERAGP**VTWVMMIACVVVFIAMQI**LGDQEVMLWLAWPFDPTLKFEFWRYFTHALMHFS**LMHILFNLLWWWYLGGAVEKRL**G**S**SGSGYPYDVPDYAFSTPVWISQAQGIRAGPGSSDKQEGEWPTGLRLSRIGGIH 201

MANMFALILVIATLVTGILWCVDKFFFAPKRRERQAAAQAAAGDSLDKATLKKVAPKPDPRYLAASWQAGHTGSGLHYRRYPFFAALRERAGP**VTWVMMIACVVVFIAMQI**LGDQEVMLWLAWPFDPTLKFEFWRYFTHALMHFS**LMHILFNLLWWWYLGGAVEKRL**GSGSGYPYDVPDYAFSTPVWISQAQGIRAGPGSSDKQEGEWPTGLRLSRIGGIH 200

MANMFALILVIATLVTGILWCVDKFFFAPKRRERQAAAQAAAGDSLDKATLKKVAPKPDPRYLAASWQAGHTGSGLHYRRYPFFAALRERAGP**VTWVMMIACVVVFIAMQI**LGDQEVMLWLAWPFDPTLKFEFWRYFTHALMHFS**LMHILFNLLWWWYLGGAVEKRL**SGSGYPYDVPDYAFSTPVWISQAQGIRAGPGSSDKQEGEWPTGLRLSRIGGIH 199

MANMFALILVIATLVTGILWCVDKFFFAPKRRERQAAAQAAAGDSLDKATLKKVAPKPDPRYLAASWQAGHTGSGLHYRRYPFFAALRERAGP**VTWVMMIACVVVFIAMQI**LGDQEVMLWLAWPFDPTLKFEFWRYFTHALMHFS**LMHILFNLLWWWYLGGAVEKR**SGSGYPYDVPDYAFSTPVWISQAQGIRAGPGSSDKQEGEWPTGLRLSRIGGIH 198

MANMFALILVIATLVTGILWCVDKFFFAPKRRERQAAAQAAAGDSLDKATLKKVAPKPDPRYLAASWQAGHTGSGLHYRRYPFFAALRERAGP**VTWVMMIACVVVFIAMQI**LGDQEVMLWLAWPFDPTLKFEFWRYFTHALMHFS**LMHILFNLLWWWYLGGAVEK**SGSGYPYDVPDYAFSTPVWISQAQGIRAGPGSSDKQEGEWPTGLRLSRIGGIH 197

MANMFALILVIATLVTGILWCVDKFFFAPKRRERQAAAQAAAGDSLDKATLKKVAPKPDPRYLAASWQAGHTGSGLHYRRYPFFAALRERAGP**VTWVMMIACVVVFIAMQI**LGDQEVMLWLAWPFDPTLKFEFWRYFTHALMHFS**LMHILFNLLWWWYLGGAVE**SGSGYPYDVPDYAFSTPVWISQAQGIRAGPGSSDKQEGEWPTGLRLSRIGGIH 196

MANMFALILVIATLVTGILWCVDKFFFAPKRRERQAAAQAAAGDSLDKATLKKVAPKPDPRYLAASWQAGHTGSGLHYRRYPFFAALRERAGP**VTWVMMIACVVVFIAMQI**LGDQEVMLWLAWPFDPTLKFEFWRYFTHALMHFS**LMHILFNLLWWWYLGGAV**SGSGYPYDVPDYAFSTPVWISQAQGIRAGPGSSDKQEGEWPTGLRLSRIGGIH 195

MANMFALILVIATLVTGILWCVDKFFFAPKRRERQAAAQAAAGDSLDKATLKKVAPKPDPRYLAASWQAGHTGSGLHYRRYPFFAALRERAGP**VTWVMMIACVVVFIAMQI**LGDQEVMLWLAWPFDPTLKFEFWRYFTHALMHFS**LMHILFNLLWWWYLGGA**SGSGYPYDVPDYAFSTPVWISQAQGIRAGPGSSDKQEGEWPTGLRLSRIGGIH 194

MANMFALILVIATLVTGILWCVDKFFFAPKRRERQAAAQAAAGDSLDKATLKKVAPKPDPRYLAASWQAGHTGSGLHYRRYPFFAALRERAGP**VTWVMMIACVVVFIAMQI**LGDQEVMLWLAWPFDPTLKFEFWRYFTHALMHFS**LMHILFNLLWWWYLGG**SGSGYPYDVPDYAFSTPVWISQAQGIRAGPGSSDKQEGEWPTGLRLSRIGGIH 193

MANMFALILVIATLVTGILWCVDKFFFAPKRRERQAAAQAAAGDSLDKATLKKVAPKPDPRYLAASWQAGHTGSGLHYRRYPFFAALRERAGP**VTWVMMIACVVVFIAMQI**LGDQEVMLWLAWPFDPTLKFEFWRYFTHALMHFS**LMHILFNLLWWWYLG**SGSGYPYDVPDYAFSTPVWISQAQGIRAGPGSSDKQEGEWPTGLRLSRIGGIH 192

MANMFALILVIATLVTGILWCVDKFFFAPKRRERQAAAQAAAGDSLDKATLKKVAPKPDPRYLAASWQAGHTGSGLHYRRYPFFAALRERAGP**VTWVMMIACVVVFIAMQI**LGDQEVMLWLAWPFDPTLKFEFWRYFTHALMHFS**LMHILFNLLWW**SGSGYPYDVPDYAFSTPVWISQAQGIRAGPGSSDKQEGEWPTGLRLSRIGGIH 188

MANMFALILVIATLVTGILWCVDKFFFAPKRRERQAAAQAAAGDSLDKATLKKVAPKPDPRYLAASWQAGHTGSGLHYRRYPFFAALRERAGP**VTWVMMIACVVVFIAMQI**LGDQEVMLWLAWPFDPTLKFEFWRYFTHALMHFS**LMHILFNLLW**SGSGYPYDVPDYAFSTPVWISQAQGIRAGPGSSDKQEGEWPTGLRLSRIGGIH 187

MANMFALILVIATLVTGILWCVDKFFFAPKRRERQAAAQAAAGDSLDKATLKKVAPKPDPRYLAASWQAGHTGSGLHYRRYPFFAALRERAGP**VTWVMMIACVVVFIAMQI**LGDQEVMLWLAWPFDPTLKFEFWRYFTHALMHFS**LMHILFNLL**SGSGYPYDVPDYAFSTPVWISQAQGIRAGPGSSDKQEGEWPTGLRLSRIGGIH 186

MANMFALILVIATLVTGILWCVDKFFFAPKRRERQAAAQAAAGDSLDKATLKKVAPKPDPRYLAASWQAGHTGSGLHYRRYPFFAALRERAGP**VTWVMMIACVVVFIAMQI**LGDQEVMLWLAWPFDPTLKFEFWRYFTHALMHFS**LMHILFNL**SGSGYPYDVPDYAFSTPVWISQAQGIRAGPGSSDKQEGEWPTGLRLSRIGGIH 185

MANMFALILVIATLVTGILWCVDKFFFAPKRRERQAAAQAAAGDSLDKATLKKVAPKPDPRYLAASWQAGHTGSGLHYRRYPFFAALRERAGP**VTWVMMIACVVVFIAMQI**LGDQEVMLWLAWPFDPTLKFEFWRYFTHALMHFS**LMHILFN**SGSGYPYDVPDYAFSTPVWISQAQGIRAGPGSSDKQEGEWPTGLRLSRIGGIH 184

MANMFALILVIATLVTGILWCVDKFFFAPKRRERQAAAQAAAGDSLDKATLKKVAPKPDPRYLAASWQAGHTGSGLHYRRYPFFAALRERAGP**VTWVMMIACVVVFIAMQI**LGDQEVMLWLAWPFDPTLKFEFWRYFTHALMHFS**LMHILF**SGSGYPYDVPDYAFSTPVWISQAQGIRAGPGSSDKQEGEWPTGLRLSRIGGIH 183

MANMFALILVIATLVTGILWCVDKFFFAPKRRERQAAAQAAAGDSLDKATLKKVAPKPDPRYLAASWQAGHTGSGLHYRRYPFFAALRERAGP**VTWVMMIACVVVFIAMQI**LGDQEVMLWLAWPFDPTLKFEFWRYFTHALMHFS**LMHIL**SGSGYPYDVPDYAFSTPVWISQAQGIRAGPGSSDKQEGEWPTGLRLSRIGGIH 182

MANMFALILVIATLVTGILWCVDKFFFAPKRRERQAAAQAAAGDSLDKATLKKVAPKPDPRYLAASWQAGHTGSGLHYRRYPFFAALRERAGP**VTWVMMIACVVVFIAMQI**LGDQEVMLWLAWPFDPTLKFEFWRYFTHALMHFS**LMHI**SGSGYPYDVPDYAFSTPVWISQAQGIRAGPGSSDKQEGEWPTGLRLSRIGGIH 181

MANMFALILVIATLVTGILWCVDKFFFAPKRRERQAAAQAAAGDSLDKATLKKVAPKPDPRYLAASWQAGHTGSGLHYRRYPFFAALRERAGP**VTWVMMIACVVVFIAMQI**LGDQEVMLWLAWPFDPTLKFEFWRYFTHALMHFS**LMH**SGSGYPYDVPDYAFSTPVWISQAQGIRAGPGSSDKQEGEWPTGLRLSRIGGIH 180

MANMFALILVIATLVTGILWCVDKFFFAPKRRERQAAAQAAAGDSLDKATLKKVAPKPDPRYLAASWQAGHTGSGLHYRRYPFFAALRERAGP**VTWVMMIACVVVFIAMQI**LGDQEVMLWLAWPFDPTLKFEFWRYFTHALMHFS**LM**SGSGYPYDVPDYAFSTPVWISQAQGIRAGPGSSDKQEGEWPTGLRLSRIGGIH 179

MANMFALILVIATLVTGILWCVDKFFFAPKRRERQAAAQAAAGDSLDKATLKKVAPKPDPRYLAASWQAGHTGSGLHYRRYPFFAALRERAGP**VTWVMMIACVVVFIAMQI**LGDQEVMLWLAWPFDPTLKFEFWRYFTHALMHFS**L**SGSGYPYDVPDYAFSTPVWISQAQGIRAGPGSSDKQEGEWPTGLRLSRIGGIH 178

MANMFALILVIATLVTGILWCVDKFFFAPKRRERQAAAQAAAGDSLDKATLKKVAPKPDPRYLAASWQAGHTGSGLHYRRYPFFAALRERAGP**VTWVMMIACVVVFIAMQI**LGDQEVMLWLAWPFDPTLKFEFWRYFTHALMHFSGSGYPYDVPDYAFSTPVWISQAQGIRAGPGSSDKQEGEWPTGLRLSRIGGIH 176

MANMFALILVIATLVTGILWCVDKFFFAPKRRERQAAAQAAAGDSLDKATLKKVAPKPDPRYLAASWQAGHTGSGLHYRRYPFFAALRERAGP**VTWVMMIACVVVFIAMQI**LGDQEVMLWLAWPFDPTLKFEFWRYFTHALMHSGSGYPYDVPDYAFSTPVWISQAQGIRAGPGSSDKQEGEWPTGLRLSRIGGIH 175

MANMFALILVIATLVTGILWCVDKFFFAPKRRERQAAAQAAAGDSLDKATLKKVAPKPDPRYLAASWQAGHTGSGLHYRRYPFFAALRERAGP**VTWVMMIACVVVFIAMQI**LGDQEVMLWLAWPFDPTLKFEFWRYFTHALMSGSGYPYDVPDYAFSTPVWISQAQGIRAGPGSSDKQEGEWPTGLRLSRIGGIH 174

MANMFALILVIATLVTGILWCVDKFFFAPKRRERQAAAQAAAGDSLDKATLKKVAPKPDPRYLAASWQAGHTGSGLHYRRYPFFAALRERAGP**VTWVMMIACVVVFIAMQI**LGDQEVMLWLAWPFDPTLKFEFWRYFTHALSGSGYPYDVPDYAFSTPVWISQAQGIRAGPGSSDKQEGEWPTGLRLSRIGGIH 173

MANMFALILVIATLVTGILWCVDKFFFAPKRRERQAAAQAAAGDSLDKATLKKVAPKPDPRYLAASWQAGHTGSGLHYRRYPFFAALRERAGP**VTWVMMIACVVVFIAMQI**LGDQEVMLWLAWPFDPTLKFEFWRYFTHASGSGYPYDVPDYAFSTPVWISQAQGIRAGPGSSDKQEGEWPTGLRLSRIGGIH 172

MANMFALILVIATLVTGILWCVDKFFFAPKRRERQAAAQAAAGDSLDKATLKKVAPKPDPRYLAASWQAGHTGSGLHYRRYPFFAALRERAGP**VTWVMMIACVVVFIAMQI**LGDQEVMLWLAWPFDPTLKFEFWRYFTHSGSGYPYDVPDYAFSTPVWISQAQGIRAGPGSSDKQEGEWPTGLRLSRIGGIH 171

MANMFALILVIATLVTGILWCVDKFFFAPKRRERQAAAQAAAGDSLDKATLKKVAPKPDPRYLAASWQAGHTGSGLHYRRYPFFAALRERAGP**VTWVMMIACVVVFIAMQI**LGDQEVMLWLAWPFDPTLKFEFWRYFTSGSGYPYDVPDYAFSTPVWISQAQGIRAGPGSSDKQEGEWPTGLRLSRIGGIH 170

MANMFALILVIATLVTGILWCVDKFFFAPKRRERQAAAQAAAGDSLDKATLKKVAPKPDPRYLAASWQAGHTGSGLHYRRYPFFAALRERAGP**VTWVMMIACVVVFIAMQI**LGDQEVMLWLAWPFDPTLKFEFWRYFSGSGYPYDVPDYAFSTPVWISQAQGIRAGPGSSDKQEGEWPTGLRLSRIGGIH 169

MANMFALILVIATLVTGILWCVDKFFFAPKRRERQAAAQAAAGDSLDKATLKKVAPKPDPRYLAASWQAGHTGSGLHYRRYPFFAALRERAGP**VTWVMMIACVVVFIAMQI**LGDQEVMLWLAWPFDPTLKFEFWRYSGSGYPYDVPDYAFSTPVWISQAQGIRAGPGSSDKQEGEWPTGLRLSRIGGIH 168

MANMFALILVIATLVTGILWCVDKFFFAPKRRERQAAAQAAAGDSLDKATLKKVAPKPDPRYLAASWQAGHTGSGLHYRRYPFFAALRERAGP**VTWVMMIACVVVFIAMQI**LGDQEVMLWLAWPFDPTLKFEFWSGSGYPYDVPDYAFSTPVWISQAQGIRAGPGSSDKQEGEWPTGLRLSRIGGIH 166

MANMFALILVIATLVTGILWCVDKFFFAPKRRERQAAAQAAAGDSLDKATLKKVAPKPDPRYLAASWQAGHTGSGLHYRRYPFFAALRERAGP**VTWVMMIACVVVFIAMQI**LGDQEVMLWLAWPFDPTLSGSGYPYDVPDYAFSTPVWISQAQGIRAGPGSSDKQEGEWPTGLRLSRIGGIH 161

MANMFALILVIATLVTGILWCVDKFFFAPKRRERQAAAQAAAGDSLDKATLKKVAPKPDPRYLAASWQAGHTGSGLHYRRYPFFAALRERAGP**VTWVMMIACVVVFIAMQI**LGDQEVMLWLAWPSGSGYPYDVPDYAFSTPVWISQAQGIRAGPGSSDKQEGEWPTGLRLSRIGGIH 156

MANMFALILVIATLVTGILWCVDKFFFAPKRRERQAAAQAAAGDSLDKATLKKVAPKPDPRYLAASWQAGHTGSGLHYRRYPFFAALRERAGP**VTWVMMIACVVVFIAMQI**LGDQEVMLSGSGYPYDVPDYAFSTPVWISQAQGIRAGPGSSDKQEGEWPTGLRLSRIGGIH 151

MANMFALILVIATLVTGILWCVDKFFFAPKRRERQAAAQAAAGDSLDKATLKKVAPKPDPRYLAASWQAGHTGSGLHYRRYPFFAALRERAGP**VTWVMMIACVVVFIAMQI**LGDSGSGYPYDVPDYAFSTPVWISQAQGIRAGPGSSDKQEGEWPTGLRLSRIGGIH 146

MANMFALILVIATLVTGILWCVDKFFFAPKRRERQAAAQAAAGDSLDKATLKKVAPKPDPRYLAASWQAGHTGSGLHYRRYPFFAALRERAGP**VTWVMMIACVVVFIAM**SGSGYPYDVPDYAFSTPVWISQAQGIRAGPGSSDKQEGEWPTGLRLSRIGGIH 141

MANMFALILVIATLVTGILWCVDKFFFAPKRRERQAAAQAAAGDSLDKATLKKVAPKPDPRYLAASWQAGHTGSGLHYRRYPFFAALRERAGP**VTWVMMIACVV**SGSGYPYDVPDYAFSTPVWISQAQGIRAGPGSSDKQEGEWPTGLRLSRIGGIH 136

MANMFALILVIATLVTGILWCVDKFFFAPKRRERQAAAQAAAGDSLDKATLKKVAPKPDPRYLAASWQAGHTGSGLHYRRYPFFAALRERAGP**VTWVMM**SGSGYPYDVPDYAFSTPVWISQAQGIRAGPGSSDKQEGEWPTGLRLSRIGGIH 131

LepB TM1 GlpG SecM(*Ec-Ms*) Single residue (*N* =171-211)

MANMFALILVIATLVTGILWCVDKFFFAPKRRERQAAAQAAAGDSLDKATLKKVAPKPDPRYLAASWQAGHTGSGLHYRRYPFFAALRERAGP**VTWVMMIACVVVFIAMQI**LGDQEVMLWLAWPFDPTLKFEFWRYFTHALMHFS**LMHILFNLLWWWYLGGAVEKRL**G**SGKLIVITLIS**SGSGYPYDVPDYAFSTPVWISQHAPIRGSPGSSDKQEGEWPTGLRLSRIGGIH 211

MANMFALILVIATLVTGILWCVDKFFFAPKRRERQAAAQAAAGDSLDKATLKKVAPKPDPRYLAASWQAGHTGSGLHYRRYPFFAALRERAGP**VTWVMMIACVVVFIAMQI**LGDQEVMLWLAWPFDPTLKFEFWRYFTHALMHFS**LMHILFNLLWWWYLGGAVEKRL**G**SGKLIVITLI**SGSGYPYDVPDYAFSTPVWISQHAPIRGSPGSSDKQEGEWPTGLRLSRIGGIH 210

MANMFALILVIATLVTGILWCVDKFFFAPKRRERQAAAQAAAGDSLDKATLKKVAPKPDPRYLAASWQAGHTGSGLHYRRYPFFAALRERAGP**VTWVMMIACVVVFIAMQI**LGDQEVMLWLAWPFDPTLKFEFWRYFTHALMHFS**LMHILFNLLWWWYLGGAVEKRL**G**SGKLIVITL**SGSGYPYDVPDYAFSTPVWISQHAPIRGSPGSSDKQEGEWPTGLRLSRIGGIH 209

MANMFALILVIATLVTGILWCVDKFFFAPKRRERQAAAQAAAGDSLDKATLKKVAPKPDPRYLAASWQAGHTGSGLHYRRYPFFAALRERAGP**VTWVMMIACVVVFIAMQI**LGDQEVMLWLAWPFDPTLKFEFWRYFTHALMHFS**LMHILFNLLWWWYLGGAVEKRL**G**SGKLIVI**SGSGYPYDVPDYAFSTPVWISQHAPIRGSPGSSDKQEGEWPTGLRLSRIGGIH 207

MANMFALILVIATLVTGILWCVDKFFFAPKRRERQAAAQAAAGDSLDKATLKKVAPKPDPRYLAASWQAGHTGSGLHYRRYPFFAALRERAGP**VTWVMMIACVVVFIAMQI**LGDQEVMLWLAWPFDPTLKFEFWRYFTHALMHFS**LMHILFNLLWWWYLGGAVEKRL**G**SGKLIV**SGSGYPYDVPDYAFSTPVWISQHAPIRGSPGSSDKQEGEWPTGLRLSRIGGIH 206

MANMFALILVIATLVTGILWCVDKFFFAPKRRERQAAAQAAAGDSLDKATLKKVAPKPDPRYLAASWQAGHTGSGLHYRRYPFFAALRERAGP**VTWVMMIACVVVFIAMQI**LGDQEVMLWLAWPFDPTLKFEFWRYFTHALMHFS**LMHILFNLLWWWYLGGAVEKRL**G**SGKLI**SGSGYPYDVPDYAFSTPVWISQHAPIRGSPGSSDKQEGEWPTGLRLSRIGGIH 205

MANMFALILVIATLVTGILWCVDKFFFAPKRRERQAAAQAAAGDSLDKATLKKVAPKPDPRYLAASWQAGHTGSGLHYRRYPFFAALRERAGP**VTWVMMIACVVVFIAMQI**LGDQEVMLWLAWPFDPTLKFEFWRYFTHALMHFS**LMHILFNLLWWWYLGGAVEKRL**G**SGKL**SGSGYPYDVPDYAFSTPVWISQHAPIRGSPGSSDKQEGEWPTGLRLSRIGGIH 204

MANMFALILVIATLVTGILWCVDKFFFAPKRRERQAAAQAAAGDSLDKATLKKVAPKPDPRYLAASWQAGHTGSGLHYRRYPFFAALRERAGP**VTWVMMIACVVVFIAMQI**LGDQEVMLWLAWPFDPTLKFEFWRYFTHALMHFS**LMHILFNLLWWWYLGGAVEKRL**G**SGK**SGSGYPYDVPDYAFSTPVWISQHAPIRGSPGSSDKQEGEWPTGLRLSRIGGIH 203

MANMFALILVIATLVTGILWCVDKFFFAPKRRERQAAAQAAAGDSLDKATLKKVAPKPDPRYLAASWQAGHTGSGLHYRRYPFFAALRERAGP**VTWVMMIACVVVFIAMQI**LGDQEVMLWLAWPFDPTLKFEFWRYFTHALMHFS**LMHILFNLLWWWYLGGAVEKRL**G**SG**SGSGYPYDVPDYAFSTPVWISQHAPIRGSPGSSDKQEGEWPTGLRLSRIGGIH 202

MANMFALILVIATLVTGILWCVDKFFFAPKRRERQAAAQAAAGDSLDKATLKKVAPKPDPRYLAASWQAGHTGSGLHYRRYPFFAALRERAGP**VTWVMMIACVVVFIAMQI**LGDQEVMLWLAWPFDPTLKFEFWRYFTHALMHFS**LMHILFNLLWWWYLGGAVEKRL**G**S**SGSGYPYDVPDYAFSTPVWISQHAPIRGSPGSSDKQEGEWPTGLRLSRIGGIH 201

MANMFALILVIATLVTGILWCVDKFFFAPKRRERQAAAQAAAGDSLDKATLKKVAPKPDPRYLAASWQAGHTGSGLHYRRYPFFAALRERAGP**VTWVMMIACVVVFIAMQI**LGDQEVMLWLAWPFDPTLKFEFWRYFTHALMHFS**LMHILFNLLWWWYLGGAVEKRL**GSGSGYPYDVPDYAFSTPVWISQHAPIRGSPGSSDKQEGEWPTGLRLSRIGGIH 200

MANMFALILVIATLVTGILWCVDKFFFAPKRRERQAAAQAAAGDSLDKATLKKVAPKPDPRYLAASWQAGHTGSGLHYRRYPFFAALRERAGP**VTWVMMIACVVVFIAMQI**LGDQEVMLWLAWPFDPTLKFEFWRYFTHALMHFS**LMHILFNLLWWWYLGGAVEKRL**SGSGYPYDVPDYAFSTPVWISQHAPIRGSPGSSDKQEGEWPTGLRLSRIGGIH 199

MANMFALILVIATLVTGILWCVDKFFFAPKRRERQAAAQAAAGDSLDKATLKKVAPKPDPRYLAASWQAGHTGSGLHYRRYPFFAALRERAGP**VTWVMMIACVVVFIAMQI**LGDQEVMLWLAWPFDPTLKFEFWRYFTHALMHFS**LMHILFNLLWWWYLGGAVEKR**SGSGYPYDVPDYAFSTPVWISQHAPIRGSPGSSDKQEGEWPTGLRLSRIGGIH 198

MANMFALILVIATLVTGILWCVDKFFFAPKRRERQAAAQAAAGDSLDKATLKKVAPKPDPRYLAASWQAGHTGSGLHYRRYPFFAALRERAGP**VTWVMMIACVVVFIAMQI**LGDQEVMLWLAWPFDPTLKFEFWRYFTHALMHFS**LMHILFNLLWWWYLGGAVEK**SGSGYPYDVPDYAFSTPVWISQHAPIRGSPGSSDKQEGEWPTGLRLSRIGGIH 197

MANMFALILVIATLVTGILWCVDKFFFAPKRRERQAAAQAAAGDSLDKATLKKVAPKPDPRYLAASWQAGHTGSGLHYRRYPFFAALRERAGP**VTWVMMIACVVVFIAMQI**LGDQEVMLWLAWPFDPTLKFEFWRYFTHALMHFS**LMHILFNLLWWWYLGGAVE**SGSGYPYDVPDYAFSTPVWISQHAPIRGSPGSSDKQEGEWPTGLRLSRIGGIH 196

MANMFALILVIATLVTGILWCVDKFFFAPKRRERQAAAQAAAGDSLDKATLKKVAPKPDPRYLAASWQAGHTGSGLHYRRYPFFAALRERAGP**VTWVMMIACVVVFIAMQI**LGDQEVMLWLAWPFDPTLKFEFWRYFTHALMHFS**LMHILFNLLWWWYLGGA**SGSGYPYDVPDYAFSTPVWISQHAPIRGSPGSSDKQEGEWPTGLRLSRIGGIH 194

MANMFALILVIATLVTGILWCVDKFFFAPKRRERQAAAQAAAGDSLDKATLKKVAPKPDPRYLAASWQAGHTGSGLHYRRYPFFAALRERAGP**VTWVMMIACVVVFIAMQI**LGDQEVMLWLAWPFDPTLKFEFWRYFTHALMHFS**LMHILFNLLWWWYLGG**SGSGYPYDVPDYAFSTPVWISQHAPIRGSPGSSDKQEGEWPTGLRLSRIGGIH 193

MANMFALILVIATLVTGILWCVDKFFFAPKRRERQAAAQAAAGDSLDKATLKKVAPKPDPRYLAASWQAGHTGSGLHYRRYPFFAALRERAGP**VTWVMMIACVVVFIAMQI**LGDQEVMLWLAWPFDPTLKFEFWRYFTHALMHFS**LMHILFNLLWWWYLG**SGSGYPYDVPDYAFSTPVWISQHAPIRGSPGSSDKQEGEWPTGLRLSRIGGIH 192

MANMFALILVIATLVTGILWCVDKFFFAPKRRERQAAAQAAAGDSLDKATLKKVAPKPDPRYLAASWQAGHTGSGLHYRRYPFFAALRERAGP**VTWVMMIACVVVFIAMQI**LGDQEVMLWLAWPFDPTLKFEFWRYFTHALMHFS**LMHILFNLLWWWYL**SGSGYPYDVPDYAFSTPVWISQHAPIRGSPGSSDKQEGEWPTGLRLSRIGGIH 191

MANMFALILVIATLVTGILWCVDKFFFAPKRRERQAAAQAAAGDSLDKATLKKVAPKPDPRYLAASWQAGHTGSGLHYRRYPFFAALRERAGP**VTWVMMIACVVVFIAMQI**LGDQEVMLWLAWPFDPTLKFEFWRYFTHALMHFS**LMHILFNLLWWWY**SGSGYPYDVPDYAFSTPVWISQHAPIRGSPGSSDKQEGEWPTGLRLSRIGGIH 190

MANMFALILVIATLVTGILWCVDKFFFAPKRRERQAAAQAAAGDSLDKATLKKVAPKPDPRYLAASWQAGHTGSGLHYRRYPFFAALRERAGP**VTWVMMIACVVVFIAMQI**LGDQEVMLWLAWPFDPTLKFEFWRYFTHALMHFS**LMHILFNLLWW**SGSGYPYDVPDYAFSTPVWISQHAPIRGSPGSSDKQEGEWPTGLRLSRIGGIH 188

MANMFALILVIATLVTGILWCVDKFFFAPKRRERQAAAQAAAGDSLDKATLKKVAPKPDPRYLAASWQAGHTGSGLHYRRYPFFAALRERAGP**VTWVMMIACVVVFIAMQI**LGDQEVMLWLAWPFDPTLKFEFWRYFTHALMHFS**LMHILFNLLW**SGSGYPYDVPDYAFSTPVWISQHAPIRGSPGSSDKQEGEWPTGLRLSRIGGIH 187

MANMFALILVIATLVTGILWCVDKFFFAPKRRERQAAAQAAAGDSLDKATLKKVAPKPDPRYLAASWQAGHTGSGLHYRRYPFFAALRERAGP**VTWVMMIACVVVFIAMQI**LGDQEVMLWLAWPFDPTLKFEFWRYFTHALMHFS**LMHILFNLL**SGSGYPYDVPDYAFSTPVWISQHAPIRGSPGSSDKQEGEWPTGLRLSRIGGIH 186

MANMFALILVIATLVTGILWCVDKFFFAPKRRERQAAAQAAAGDSLDKATLKKVAPKPDPRYLAASWQAGHTGSGLHYRRYPFFAALRERAGP**VTWVMMIACVVVFIAMQI**LGDQEVMLWLAWPFDPTLKFEFWRYFTHALMHFS**LMHILFNL**SGSGYPYDVPDYAFSTPVWISQHAPIRGSPGSSDKQEGEWPTGLRLSRIGGIH 185

MANMFALILVIATLVTGILWCVDKFFFAPKRRERQAAAQAAAGDSLDKATLKKVAPKPDPRYLAASWQAGHTGSGLHYRRYPFFAALRERAGP**VTWVMMIACVVVFIAMQI**LGDQEVMLWLAWPFDPTLKFEFWRYFTHALMHFS**LMHILFN**SGSGYPYDVPDYAFSTPVWISQHAPIRGSPGSSDKQEGEWPTGLRLSRIGGIH 184

MANMFALILVIATLVTGILWCVDKFFFAPKRRERQAAAQAAAGDSLDKATLKKVAPKPDPRYLAASWQAGHTGSGLHYRRYPFFAALRERAGP**VTWVMMIACVVVFIAMQI**LGDQEVMLWLAWPFDPTLKFEFWRYFTHALMHFS**LMHILF**SGSGYPYDVPDYAFSTPVWISQHAPIRGSPGSSDKQEGEWPTGLRLSRIGGIH 183

MANMFALILVIATLVTGILWCVDKFFFAPKRRERQAAAQAAAGDSLDKATLKKVAPKPDPRYLAASWQAGHTGSGLHYRRYPFFAALRERAGP**VTWVMMIACVVVFIAMQI**LGDQEVMLWLAWPFDPTLKFEFWRYFTHALMHFS**LMHIL**SGSGYPYDVPDYAFSTPVWISQHAPIRGSPGSSDKQEGEWPTGLRLSRIGGIH 182

MANMFALILVIATLVTGILWCVDKFFFAPKRRERQAAAQAAAGDSLDKATLKKVAPKPDPRYLAASWQAGHTGSGLHYRRYPFFAALRERAGP**VTWVMMIACVVVFIAMQI**LGDQEVMLWLAWPFDPTLKFEFWRYFTHALMHFS**LMHI**SGSGYPYDVPDYAFSTPVWISQHAPIRGSPGSSDKQEGEWPTGLRLSRIGGIH 181

MANMFALILVIATLVTGILWCVDKFFFAPKRRERQAAAQAAAGDSLDKATLKKVAPKPDPRYLAASWQAGHTGSGLHYRRYPFFAALRERAGP**VTWVMMIACVVVFIAMQI**LGDQEVMLWLAWPFDPTLKFEFWRYFTHALMHFS**LMH**SGSGYPYDVPDYAFSTPVWISQHAPIRGSPGSSDKQEGEWPTGLRLSRIGGIH 180

MANMFALILVIATLVTGILWCVDKFFFAPKRRERQAAAQAAAGDSLDKATLKKVAPKPDPRYLAASWQAGHTGSGLHYRRYPFFAALRERAGP**VTWVMMIACVVVFIAMQI**LGDQEVMLWLAWPFDPTLKFEFWRYFTHALMHFS**LM**SGSGYPYDVPDYAFSTPVWISQHAPIRGSPGSSDKQEGEWPTGLRLSRIGGIH 179

MANMFALILVIATLVTGILWCVDKFFFAPKRRERQAAAQAAAGDSLDKATLKKVAPKPDPRYLAASWQAGHTGSGLHYRRYPFFAALRERAGP**VTWVMMIACVVVFIAMQI**LGDQEVMLWLAWPFDPTLKFEFWRYFTHALMHFS**L**SGSGYPYDVPDYAFSTPVWISQHAPIRGSPGSSDKQEGEWPTGLRLSRIGGIH 178

MANMFALILVIATLVTGILWCVDKFFFAPKRRERQAAAQAAAGDSLDKATLKKVAPKPDPRYLAASWQAGHTGSGLHYRRYPFFAALRERAGP**VTWVMMIACVVVFIAMQI**LGDQEVMLWLAWPFDPTLKFEFWRYFTHALMHFSSGSGYPYDVPDYAFSTPVWISQHAPIRGSPGSSDKQEGEWPTGLRLSRIGGIH 177

MANMFALILVIATLVTGILWCVDKFFFAPKRRERQAAAQAAAGDSLDKATLKKVAPKPDPRYLAASWQAGHTGSGLHYRRYPFFAALRERAGP**VTWVMMIACVVVFIAMQI**LGDQEVMLWLAWPFDPTLKFEFWRYFTHALMHFSGSGYPYDVPDYAFSTPVWISQHAPIRGSPGSSDKQEGEWPTGLRLSRIGGIH 176

MANMFALILVIATLVTGILWCVDKFFFAPKRRERQAAAQAAAGDSLDKATLKKVAPKPDPRYLAASWQAGHTGSGLHYRRYPFFAALRERAGP**VTWVMMIACVVVFIAMQI**LGDQEVMLWLAWPFDPTLKFEFWRYFTHALMHSGSGYPYDVPDYAFSTPVWISQHAPIRGSPGSSDKQEGEWPTGLRLSRIGGIH 175

MANMFALILVIATLVTGILWCVDKFFFAPKRRERQAAAQAAAGDSLDKATLKKVAPKPDPRYLAASWQAGHTGSGLHYRRYPFFAALRERAGP**VTWVMMIACVVVFIAMQI**LGDQEVMLWLAWPFDPTLKFEFWRYFTHALMSGSGYPYDVPDYAFSTPVWISQHAPIRGSPGSSDKQEGEWPTGLRLSRIGGIH 174

MANMFALILVIATLVTGILWCVDKFFFAPKRRERQAAAQAAAGDSLDKATLKKVAPKPDPRYLAASWQAGHTGSGLHYRRYPFFAALRERAGP**VTWVMMIACVVVFIAMQI**LGDQEVMLWLAWPFDPTLKFEFWRYFTHALSGSGYPYDVPDYAFSTPVWISQHAPIRGSPGSSDKQEGEWPTGLRLSRIGGIH 173

MANMFALILVIATLVTGILWCVDKFFFAPKRRERQAAAQAAAGDSLDKATLKKVAPKPDPRYLAASWQAGHTGSGLHYRRYPFFAALRERAGP**VTWVMMIACVVVFIAMQI**LGDQEVMLWLAWPFDPTLKFEFWRYFTHASGSGYPYDVPDYAFSTPVWISQHAPIRGSPGSSDKQEGEWPTGLRLSRIGGIH 172

MANMFALILVIATLVTGILWCVDKFFFAPKRRERQAAAQAAAGDSLDKATLKKVAPKPDPRYLAASWQAGHTGSGLHYRRYPFFAALRERAGP**VTWVMMIACVVVFIAMQI**LGDQEVMLWLAWPFDPTLKFEFWRYFTHSGSGYPYDVPDYAFSTPVWISQHAPIRGSPGSSDKQEGEWPTGLRLSRIGGIH 171

LepB TM1 GlpG SecM(*Ec*-Sup1) Single residue (*N*=170-204)

HA-tag SecM (*Sup1)*

MANMFALILVIATLVTGILWCVDKFFFAPKRRERQAAAQAAAGDSLDKATLKKVAPKPDPRYLAASWQAGHTGSGLHYRRYPFFAALRERAGP**VTWVMMIACVVVFIAMQI**LGDQEVMLWLAWPFDPTLKFEFWRYFTHALMHFS**LMHILFNLLWWWYLGGAVEKRL**GSGKLSGSGYPYDVPDYAFSTPVWISQAPPIRAGPGSSDKQEGEWPTGLRLSRIGGIH 204

MANMFALILVIATLVTGILWCVDKFFFAPKRRERQAAAQAAAGDSLDKATLKKVAPKPDPRYLAASWQAGHTGSGLHYRRYPFFAALRERAGP**VTWVMMIACVVVFIAMQI**LGDQEVMLWLAWPFDPTLKFEFWRYFTHALMHFS**LMHILFNLLWWWYLGGAVEKRL**GSGKSGSGYPYDVPDYAFSTPVWISQAPPIRAGPGSSDKQEGEWPTGLRLSRIGGIH 203

MANMFALILVIATLVTGILWCVDKFFFAPKRRERQAAAQAAAGDSLDKATLKKVAPKPDPRYLAASWQAGHTGSGLHYRRYPFFAALRERAGP**VTWVMMIACVVVFIAMQI**LGDQEVMLWLAWPFDPTLKFEFWRYFTHALMHFS**LMHILFNLLWWWYLGGAVEKRL**GSGSGSGYPYDVPDYAFSTPVWISQAPPIRAGPGSSDKQEGEWPTGLRLSRIGGIH 202

MANMFALILVIATLVTGILWCVDKFFFAPKRRERQAAAQAAAGDSLDKATLKKVAPKPDPRYLAASWQAGHTGSGLHYRRYPFFAALRERAGP**VTWVMMIACVVVFIAMQI**LGDQEVMLWLAWPFDPTLKFEFWRYFTHALMHFS**LMHILFNLLWWWYLGGAVEKRL**GSSGSGYPYDVPDYAFSTPVWISQAPPIRAGPGSSDKQEGEWPTGLRLSRIGGIH 201

MANMFALILVIATLVTGILWCVDKFFFAPKRRERQAAAQAAAGDSLDKATLKKVAPKPDPRYLAASWQAGHTGSGLHYRRYPFFAALRERAGP**VTWVMMIACVVVFIAMQI**LGDQEVMLWLAWPFDPTLKFEFWRYFTHALMHFS**LMHILFNLLWWWYLGGAVEKRL**GSGSGYPYDVPDYAFSTPVWISQAPPIRAGPGSSDKQEGEWPTGLRLSRIGGIH 200

MANMFALILVIATLVTGILWCVDKFFFAPKRRERQAAAQAAAGDSLDKATLKKVAPKPDPRYLAASWQAGHTGSGLHYRRYPFFAALRERAGP**VTWVMMIACVVVFIAMQI**LGDQEVMLWLAWPFDPTLKFEFWRYFTHALMHFS**LMHILFNLLWWWYLGGAVEKRL**SGSGYPYDVPDYAFSTPVWISQAPPIRAGPGSSDKQEGEWPTGLRLSRIGGIH 199

MANMFALILVIATLVTGILWCVDKFFFAPKRRERQAAAQAAAGDSLDKATLKKVAPKPDPRYLAASWQAGHTGSGLHYRRYPFFAALRERAGP**VTWVMMIACVVVFIAMQI**LGDQEVMLWLAWPFDPTLKFEFWRYFTHALMHFS**LMHILFNLLWWWYLGGAVEKR**SGSGYPYDVPDYAFSTPVWISQAPPIRAGPGSSDKQEGEWPTGLRLSRIGGIH 198

MANMFALILVIATLVTGILWCVDKFFFAPKRRERQAAAQAAAGDSLDKATLKKVAPKPDPRYLAASWQAGHTGSGLHYRRYPFFAALRERAGP**VTWVMMIACVVVFIAMQI**LGDQEVMLWLAWPFDPTLKFEFWRYFTHALMHFS**LMHILFNLLWWWYLGGAVEK**SGSGYPYDVPDYAFSTPVWISQAPPIRAGPGSSDKQEGEWPTGLRLSRIGGIH 197

MANMFALILVIATLVTGILWCVDKFFFAPKRRERQAAAQAAAGDSLDKATLKKVAPKPDPRYLAASWQAGHTGSGLHYRRYPFFAALRERAGP**VTWVMMIACVVVFIAMQI**LGDQEVMLWLAWPFDPTLKFEFWRYFTHALMHFS**LMHILFNLLWWWYLGGAVE**SGSGYPYDVPDYAFSTPVWISQAPPIRAGPGSSDKQEGEWPTGLRLSRIGGIH 196

MANMFALILVIATLVTGILWCVDKFFFAPKRRERQAAAQAAAGDSLDKATLKKVAPKPDPRYLAASWQAGHTGSGLHYRRYPFFAALRERAGP**VTWVMMIACVVVFIAMQI**LGDQEVMLWLAWPFDPTLKFEFWRYFTHALMHFS**LMHILFNLLWWWYLGGAV**SGSGYPYDVPDYAFSTPVWISQAPPIRAGPGSSDKQEGEWPTGLRLSRIGGIH 195

MANMFALILVIATLVTGILWCVDKFFFAPKRRERQAAAQAAAGDSLDKATLKKVAPKPDPRYLAASWQAGHTGSGLHYRRYPFFAALRERAGP**VTWVMMIACVVVFIAMQI**LGDQEVMLWLAWPFDPTLKFEFWRYFTHALMHFS**LMHILFNLLWWWYLGGA**SGSGYPYDVPDYAFSTPVWISQAPPIRAGPGSSDKQEGEWPTGLRLSRIGGIH 194

MANMFALILVIATLVTGILWCVDKFFFAPKRRERQAAAQAAAGDSLDKATLKKVAPKPDPRYLAASWQAGHTGSGLHYRRYPFFAALRERAGP**VTWVMMIACVVVFIAMQI**LGDQEVMLWLAWPFDPTLKFEFWRYFTHALMHFS**LMHILFNLLWWWYLGG**SGSGYPYDVPDYAFSTPVWISQAPPIRAGPGSSDKQEGEWPTGLRLSRIGGIH 193

MANMFALILVIATLVTGILWCVDKFFFAPKRRERQAAAQAAAGDSLDKATLKKVAPKPDPRYLAASWQAGHTGSGLHYRRYPFFAALRERAGP**VTWVMMIACVVVFIAMQI**LGDQEVMLWLAWPFDPTLKFEFWRYFTHALMHFS**LMHILFNLLWWWYLG**SGSGYPYDVPDYAFSTPVWISQAPPIRAGPGSSDKQEGEWPTGLRLSRIGGIH 192

MANMFALILVIATLVTGILWCVDKFFFAPKRRERQAAAQAAAGDSLDKATLKKVAPKPDPRYLAASWQAGHTGSGLHYRRYPFFAALRERAGP**VTWVMMIACVVVFIAMQI**LGDQEVMLWLAWPFDPTLKFEFWRYFTHALMHFS**LMHILFNLLWWWYL**SGSGYPYDVPDYAFSTPVWISQAPPIRAGPGSSDKQEGEWPTGLRLSRIGGIH 191

MANMFALILVIATLVTGILWCVDKFFFAPKRRERQAAAQAAAGDSLDKATLKKVAPKPDPRYLAASWQAGHTGSGLHYRRYPFFAALRERAGP**VTWVMMIACVVVFIAMQI**LGDQEVMLWLAWPFDPTLKFEFWRYFTHALMHFS**LMHILFNLLWWWY**SGSGYPYDVPDYAFSTPVWISQAPPIRAGPGSSDKQEGEWPTGLRLSRIGGIH 190

MANMFALILVIATLVTGILWCVDKFFFAPKRRERQAAAQAAAGDSLDKATLKKVAPKPDPRYLAASWQAGHTGSGLHYRRYPFFAALRERAGP**VTWVMMIACVVVFIAMQI**LGDQEVMLWLAWPFDPTLKFEFWRYFTHALMHFS**LMHILFNLLWWW**SGSGYPYDVPDYAFSTPVWISQAPPIRAGPGSSDKQEGEWPTGLRLSRIGGIH 189

MANMFALILVIATLVTGILWCVDKFFFAPKRRERQAAAQAAAGDSLDKATLKKVAPKPDPRYLAASWQAGHTGSGLHYRRYPFFAALRERAGP**VTWVMMIACVVVFIAMQI**LGDQEVMLWLAWPFDPTLKFEFWRYFTHALMHFS**LMHILFNLLWW**SGSGYPYDVPDYAFSTPVWISQAPPIRAGPGSSDKQEGEWPTGLRLSRIGGIH 188

MANMFALILVIATLVTGILWCVDKFFFAPKRRERQAAAQAAAGDSLDKATLKKVAPKPDPRYLAASWQAGHTGSGLHYRRYPFFAALRERAGP**VTWVMMIACVVVFIAMQI**LGDQEVMLWLAWPFDPTLKFEFWRYFTHALMHFS**LMHILFNLLW**SGSGYPYDVPDYAFSTPVWISQAPPIRAGPGSSDKQEGEWPTGLRLSRIGGIH 187

MANMFALILVIATLVTGILWCVDKFFFAPKRRERQAAAQAAAGDSLDKATLKKVAPKPDPRYLAASWQAGHTGSGLHYRRYPFFAALRERAGP**VTWVMMIACVVVFIAMQI**LGDQEVMLWLAWPFDPTLKFEFWRYFTHALMHFS**LMHILFNLL**SGSGYPYDVPDYAFSTPVWISQAPPIRAGPGSSDKQEGEWPTGLRLSRIGGIH 186

MANMFALILVIATLVTGILWCVDKFFFAPKRRERQAAAQAAAGDSLDKATLKKVAPKPDPRYLAASWQAGHTGSGLHYRRYPFFAALRERAGP**VTWVMMIACVVVFIAMQI**LGDQEVMLWLAWPFDPTLKFEFWRYFTHALMHFS**LMHILFNL**SGSGYPYDVPDYAFSTPVWISQAPPIRAGPGSSDKQEGEWPTGLRLSRIGGIH 185

MANMFALILVIATLVTGILWCVDKFFFAPKRRERQAAAQAAAGDSLDKATLKKVAPKPDPRYLAASWQAGHTGSGLHYRRYPFFAALRERAGP**VTWVMMIACVVVFIAMQI**LGDQEVMLWLAWPFDPTLKFEFWRYFTHALMHFS**LMHILFN**SGSGYPYDVPDYAFSTPVWISQAPPIRAGPGSSDKQEGEWPTGLRLSRIGGIH 184

MANMFALILVIATLVTGILWCVDKFFFAPKRRERQAAAQAAAGDSLDKATLKKVAPKPDPRYLAASWQAGHTGSGLHYRRYPFFAALRERAGP**VTWVMMIACVVVFIAMQI**LGDQEVMLWLAWPFDPTLKFEFWRYFTHALMHFS**LMHILF**SGSGYPYDVPDYAFSTPVWISQAPPIRAGPGSSDKQEGEWPTGLRLSRIGGIH 183

MANMFALILVIATLVTGILWCVDKFFFAPKRRERQAAAQAAAGDSLDKATLKKVAPKPDPRYLAASWQAGHTGSGLHYRRYPFFAALRERAGP**VTWVMMIACVVVFIAMQI**LGDQEVMLWLAWPFDPTLKFEFWRYFTHALMHFS**LMHIL**SGSGYPYDVPDYAFSTPVWISQAPPIRAGPGSSDKQEGEWPTGLRLSRIGGIH 182

MANMFALILVIATLVTGILWCVDKFFFAPKRRERQAAAQAAAGDSLDKATLKKVAPKPDPRYLAASWQAGHTGSGLHYRRYPFFAALRERAGP**VTWVMMIACVVVFIAMQI**LGDQEVMLWLAWPFDPTLKFEFWRYFTHALMHFS**LMHI**SGSGYPYDVPDYAFSTPVWISQAPPIRAGPGSSDKQEGEWPTGLRLSRIGGIH 181

MANMFALILVIATLVTGILWCVDKFFFAPKRRERQAAAQAAAGDSLDKATLKKVAPKPDPRYLAASWQAGHTGSGLHYRRYPFFAALRERAGP**VTWVMMIACVVVFIAMQI**LGDQEVMLWLAWPFDPTLKFEFWRYFTHALMHFS**LMH**SGSGYPYDVPDYAFSTPVWISQAPPIRAGPGSSDKQEGEWPTGLRLSRIGGIH 180

MANMFALILVIATLVTGILWCVDKFFFAPKRRERQAAAQAAAGDSLDKATLKKVAPKPDPRYLAASWQAGHTGSGLHYRRYPFFAALRERAGP**VTWVMMIACVVVFIAMQI**LGDQEVMLWLAWPFDPTLKFEFWRYFTHALMHFS**LM**SGSGYPYDVPDYAFSTPVWISQAPPIRAGPGSSDKQEGEWPTGLRLSRIGGIH 179

MANMFALILVIATLVTGILWCVDKFFFAPKRRERQAAAQAAAGDSLDKATLKKVAPKPDPRYLAASWQAGHTGSGLHYRRYPFFAALRERAGP**VTWVMMIACVVVFIAMQI**LGDQEVMLWLAWPFDPTLKFEFWRYFTHALMHFS**L**SGSGYPYDVPDYAFSTPVWISQAPPIRAGPGSSDKQEGEWPTGLRLSRIGGIH 178

MANMFALILVIATLVTGILWCVDKFFFAPKRRERQAAAQAAAGDSLDKATLKKVAPKPDPRYLAASWQAGHTGSGLHYRRYPFFAALRERAGP**VTWVMMIACVVVFIAMQI**LGDQEVMLWLAWPFDPTLKFEFWRYFTHALMHFSSGSGYPYDVPDYAFSTPVWISQAPPIRAGPGSSDKQEGEWPTGLRLSRIGGIH 177

MANMFALILVIATLVTGILWCVDKFFFAPKRRERQAAAQAAAGDSLDKATLKKVAPKPDPRYLAASWQAGHTGSGLHYRRYPFFAALRERAGP**VTWVMMIACVVVFIAMQI**LGDQEVMLWLAWPFDPTLKFEFWRYFTHALMHFSGSGYPYDVPDYAFSTPVWISQAPPIRAGPGSSDKQEGEWPTGLRLSRIGGIH 176

MANMFALILVIATLVTGILWCVDKFFFAPKRRERQAAAQAAAGDSLDKATLKKVAPKPDPRYLAASWQAGHTGSGLHYRRYPFFAALRERAGP**VTWVMMIACVVVFIAMQI**LGDQEVMLWLAWPFDPTLKFEFWRYFTHALMHSGSGYPYDVPDYAFSTPVWISQAPPIRAGPGSSDKQEGEWPTGLRLSRIGGIH 175

MANMFALILVIATLVTGILWCVDKFFFAPKRRERQAAAQAAAGDSLDKATLKKVAPKPDPRYLAASWQAGHTGSGLHYRRYPFFAALRERAGP**VTWVMMIACVVVFIAMQI**LGDQEVMLWLAWPFDPTLKFEFWRYFTHALMSGSGYPYDVPDYAFSTPVWISQAPPIRAGPGSSDKQEGEWPTGLRLSRIGGIH 174

MANMFALILVIATLVTGILWCVDKFFFAPKRRERQAAAQAAAGDSLDKATLKKVAPKPDPRYLAASWQAGHTGSGLHYRRYPFFAALRERAGP**VTWVMMIACVVVFIAMQI**LGDQEVMLWLAWPFDPTLKFEFWRYFTHALSGSGYPYDVPDYAFSTPVWISQAPPIRAGPGSSDKQEGEWPTGLRLSRIGGIH 173

MANMFALILVIATLVTGILWCVDKFFFAPKRRERQAAAQAAAGDSLDKATLKKVAPKPDPRYLAASWQAGHTGSGLHYRRYPFFAALRERAGP**VTWVMMIACVVVFIAMQI**LGDQEVMLWLAWPFDPTLKFEFWRYFTHASGSGYPYDVPDYAFSTPVWISQAPPIRAGPGSSDKQEGEWPTGLRLSRIGGIH 172

MANMFALILVIATLVTGILWCVDKFFFAPKRRERQAAAQAAAGDSLDKATLKKVAPKPDPRYLAASWQAGHTGSGLHYRRYPFFAALRERAGP**VTWVMMIACVVVFIAMQI**LGDQEVMLWLAWPFDPTLKFEFWRYFTHSGSGYPYDVPDYAFSTPVWISQAPPIRAGPGSSDKQEGEWPTGLRLSRIGGIH 171

MANMFALILVIATLVTGILWCVDKFFFAPKRRERQAAAQAAAGDSLDKATLKKVAPKPDPRYLAASWQAGHTGSGLHYRRYPFFAALRERAGP**VTWVMMIACVVVFIAMQI**LGDQEVMLWLAWPFDPTLKFEFWRYFTSGSGYPYDVPDYAFSTPVWISQAPPIRAGPGSSDKQEGEWPTGLRLSRIGGIH 170

LepB TM1 GlpG SecM(*Ec-Ms*) variant Y^138^F^139^L^143^→NNN Single residue (*N*=182-213)

MANMFALILVIATLVTGILWCVDKFFFAPKRRERQAAAQAAAGDSLDKATLKKVAPKPDPRYLAASWQAGHTGSGLHYRRYPFFAALRERAGP**VTWVMMIACVVVFIAMQI**LGDQEVMLWLAWPFDPTLKFEFWR**NN**THA**N**MHFS**LMHILFNLLWWWYLGGAVEKRL**G**SGKLIVITLISAL**SGSGYPYDVPDYAFSTPVWISQHAPIRGSPGSSDKQEGEWPTGLRLSRIGGIH 213

MANMFALILVIATLVTGILWCVDKFFFAPKRRERQAAAQAAAGDSLDKATLKKVAPKPDPRYLAASWQAGHTGSGLHYRRYPFFAALRERAGP**VTWVMMIACVVVFIAMQI**LGDQEVMLWLAWPFDPTLKFEFWR**NN**THA**N**MHFS**LMHILFNLLWWWYLGGAVEKRL**G**SGKLIVITLISA**SGSGYPYDVPDYAFSTPVWISQHAPIRGSPGSSDKQEGEWPTGLRLSRIGGIH 212

MANMFALILVIATLVTGILWCVDKFFFAPKRRERQAAAQAAAGDSLDKATLKKVAPKPDPRYLAASWQAGHTGSGLHYRRYPFFAALRERAGP**VTWVMMIACVVVFIAMQI**LGDQEVMLWLAWPFDPTLKFEFWR**NN**THA**N**MHFS**LMHILFNLLWWWYLGGAVEKRL**G**SGKLIVITLIS**SGSGYPYDVPDYAFSTPVWISQHAPIRGSPGSSDKQEGEWPTGLRLSRIGGIH 211

MANMFALILVIATLVTGILWCVDKFFFAPKRRERQAAAQAAAGDSLDKATLKKVAPKPDPRYLAASWQAGHTGSGLHYRRYPFFAALRERAGP**VTWVMMIACVVVFIAMQI**LGDQEVMLWLAWPFDPTLKFEFWR**NN**THA**N**MHFS**LMHILFNLLWWWYLGGAVEKRL**G**SGKLIVITLI**SGSGYPYDVPDYAFSTPVWISQHAPIRGSPGSSDKQEGEWPTGLRLSRIGGIH 210

MANMFALILVIATLVTGILWCVDKFFFAPKRRERQAAAQAAAGDSLDKATLKKVAPKPDPRYLAASWQAGHTGSGLHYRRYPFFAALRERAGP**VTWVMMIACVVVFIAMQI**LGDQEVMLWLAWPFDPTLKFEFWR**NN**THA**N**MHFS**LMHILFNLLWWWYLGGAVEKRL**G**SGKLIVI**SGSGYPYDVPDYAFSTPVWISQHAPIRGSPGSSDKQEGEWPTGLRLSRIGGIH 207

MANMFALILVIATLVTGILWCVDKFFFAPKRRERQAAAQAAAGDSLDKATLKKVAPKPDPRYLAASWQAGHTGSGLHYRRYPFFAALRERAGP**VTWVMMIACVVVFIAMQI**LGDQEVMLWLAWPFDPTLKFEFWR**NN**THA**N**MHFS**LMHILFNLLWWWYLGGAVEKRL**G**SGKLIV**SGSGYPYDVPDYAFSTPVWISQHAPIRGSPGSSDKQEGEWPTGLRLSRIGGIH 206

MANMFALILVIATLVTGILWCVDKFFFAPKRRERQAAAQAAAGDSLDKATLKKVAPKPDPRYLAASWQAGHTGSGLHYRRYPFFAALRERAGP**VTWVMMIACVVVFIAMQI**LGDQEVMLWLAWPFDPTLKFEFWR**NN**THA**N**MHFS**LMHILFNLLWWWYLGGAVEKRL**G**SGKLI**SGSGYPYDVPDYAFSTPVWISQHAPIRGSPGSSDKQEGEWPTGLRLSRIGGIH 205

MANMFALILVIATLVTGILWCVDKFFFAPKRRERQAAAQAAAGDSLDKATLKKVAPKPDPRYLAASWQAGHTGSGLHYRRYPFFAALRERAGP**VTWVMMIACVVVFIAMQI**LGDQEVMLWLAWPFDPTLKFEFWR**NN**THA**N**MHFS**LMHILFNLLWWWYLGGAVEKRL**G**SGK**SGSGYPYDVPDYAFSTPVWISQHAPIRGSPGSSDKQEGEWPTGLRLSRIGGIH 203

MANMFALILVIATLVTGILWCVDKFFFAPKRRERQAAAQAAAGDSLDKATLKKVAPKPDPRYLAASWQAGHTGSGLHYRRYPFFAALRERAGPVTWVMMIACVVVFIAMQILGDQEVMLWLAWPFDPTLKFEFWR**NN**THA**N**MHFS**LMHILFNLLWWWYLGGAVEKRL**G**SG**SGSGYPYDVPDYAFSTPVWISQHAPIRGSPGSSDKQEGEWPTGLRLSRIGGIH 202

MANMFALILVIATLVTGILWCVDKFFFAPKRRERQAAAQAAAGDSLDKATLKKVAPKPDPRYLAASWQAGHTGSGLHYRRYPFFAALRERAGP**VTWVMMIACVVVFIAMQI**LGDQEVMLWLAWPFDPTLKFEFWR**NN**THA**N**MHFS**LMHILFNLLWWWYLGGAVEKRL**GSGSGYPYDVPDYAFSTPVWISQHAPIRGSPGSSDKQEGEWPTGLRLSRIGGIH 200

MANMFALILVIATLVTGILWCVDKFFFAPKRRERQAAAQAAAGDSLDKATLKKVAPKPDPRYLAASWQAGHTGSGLHYRRYPFFAALRERAGP**VTWVMMIACVVVFIAMQI**LGDQEVMLWLAWPFDPTLKFEFWR**NN**THA**N**MHFS**LMHILFNLLWWWYLGGAVEKRL**SGSGYPYDVPDYAFSTPVWISQHAPIRGSPGSSDKQEGEWPTGLRLSRIGGIH 199

MANMFALILVIATLVTGILWCVDKFFFAPKRRERQAAAQAAAGDSLDKATLKKVAPKPDPRYLAASWQAGHTGSGLHYRRYPFFAALRERAGP**VTWVMMIACVVVFIAMQI**LGDQEVMLWLAWPFDPTLKFEFWR**NN**THA**N**MHFS**LMHILFNLLWWWYLGGAVEKR**SGSGYPYDVPDYAFSTPVWISQHAPIRGSPGSSDKQEGEWPTGLRLSRIGGIH 198

MANMFALILVIATLVTGILWCVDKFFFAPKRRERQAAAQAAAGDSLDKATLKKVAPKPDPRYLAASWQAGHTGSGLHYRRYPFFAALRERAGP**VTWVMMIACVVVFIAMQI**LGDQEVMLWLAWPFDPTLKFEFWR**NN**THA**N**MHFS**LMHILFNLLWWWYLGGAVEK**SGSGYPYDVPDYAFSTPVWISQHAPIRGSPGSSDKQEGEWPTGLRLSRIGGIH 197

MANMFALILVIATLVTGILWCVDKFFFAPKRRERQAAAQAAAGDSLDKATLKKVAPKPDPRYLAASWQAGHTGSGLHYRRYPFFAALRERAGP**VTWVMMIACVVVFIAMQI**LGDQEVMLWLAWPFDPTLKFEFWR**NN**THA**N**MHFS**LMHILFNLLWWWYLGGAVE**SGSGYPYDVPDYAFSTPVWISQHAPIRGSPGSSDKQEGEWPTGLRLSRIGGIH 196

MANMFALILVIATLVTGILWCVDKFFFAPKRRERQAAAQAAAGDSLDKATLKKVAPKPDPRYLAASWQAGHTGSGLHYRRYPFFAALRERAGP**VTWVMMIACVVVFIAMQI**LGDQEVMLWLAWPFDPTLKFEFWR**NN**THA**N**MHFS**LMHILFNLLWWWYLGGA**SGSGYPYDVPDYAFSTPVWISQHAPIRGSPGSSDKQEGEWPTGLRLSRIGGIH 194

MANMFALILVIATLVTGILWCVDKFFFAPKRRERQAAAQAAAGDSLDKATLKKVAPKPDPRYLAASWQAGHTGSGLHYRRYPFFAALRERAGP**VTWVMMIACVVVFIAMQI**LGDQEVMLWLAWPFDPTLKFEFWR**NN**THA**N**MHFS**LMHILFNLLWWWYLGG**SGSGYPYDVPDYAFSTPVWISQHAPIRGSPGSSDKQEGEWPTGLRLSRIGGIH 193

MANMFALILVIATLVTGILWCVDKFFFAPKRRERQAAAQAAAGDSLDKATLKKVAPKPDPRYLAASWQAGHTGSGLHYRRYPFFAALRERAGP**VTWVMMIACVVVFIAMQI**LGDQEVMLWLAWPFDPTLKFEFWR**NN**THA**N**MHFS**LMHILFNLLWWWYLG**SGSGYPYDVPDYAFSTPVWISQHAPIRGSPGSSDKQEGEWPTGLRLSRIGGIH 192

MANMFALILVIATLVTGILWCVDKFFFAPKRRERQAAAQAAAGDSLDKATLKKVAPKPDPRYLAASWQAGHTGSGLHYRRYPFFAALRERAGP**VTWVMMIACVVVFIAMQI**LGDQEVMLWLAWPFDPTLKFEFWR**NN**THA**N**MHFS**LMHILFNLLWWWYL**SGSGYPYDVPDYAFSTPVWISQHAPIRGSPGSSDKQEGEWPTGLRLSRIGGIH 191

MANMFALILVIATLVTGILWCVDKFFFAPKRRERQAAAQAAAGDSLDKATLKKVAPKPDPRYLAASWQAGHTGSGLHYRRYPFFAALRERAGP**VTWVMMIACVVVFIAMQI**LGDQEVMLWLAWPFDPTLKFEFWR**NN**THA**N**MHFS**LMHILFNLLWWWY**SGSGYPYDVPDYAFSTPVWISQHAPIRGSPGSSDKQEGEWPTGLRLSRIGGIH 190

MANMFALILVIATLVTGILWCVDKFFFAPKRRERQAAAQAAAGDSLDKATLKKVAPKPDPRYLAASWQAGHTGSGLHYRRYPFFAALRERAGP**VTWVMMIACVVVFIAMQI**LGDQEVMLWLAWPFDPTLKFEFWR**NN**THA**N**MHFS**LMHILFNLLWWW**SGSGYPYDVPDYAFSTPVWISQHAPIRGSPGSSDKQEGEWPTGLRLSRIGGIH 189

MANMFALILVIATLVTGILWCVDKFFFAPKRRERQAAAQAAAGDSLDKATLKKVAPKPDPRYLAASWQAGHTGSGLHYRRYPFFAALRERAGP**VTWVMMIACVVVFIAMQI**LGDQEVMLWLAWPFDPTLKFEFWR**NN**THA**N**MHFS**LMHILFNLLWW**SGSGYPYDVPDYAFSTPVWISQHAPIRGSPGSSDKQEGEWPTGLRLSRIGGIH 188

MANMFALILVIATLVTGILWCVDKFFFAPKRRERQAAAQAAAGDSLDKATLKKVAPKPDPRYLAASWQAGHTGSGLHYRRYPFFAALRERAGP**VTWVMMIACVVVFIAMQI**LGDQEVMLWLAWPFDPTLKFEFWR**NN**THA**N**MHFS**LMHILFNLLW**SGSGYPYDVPDYAFSTPVWISQHAPIRGSPGSSDKQEGEWPTGLRLSRIGGIH 187

MANMFALILVIATLVTGILWCVDKFFFAPKRRERQAAAQAAAGDSLDKATLKKVAPKPDPRYLAASWQAGHTGSGLHYRRYPFFAALRERAGP**VTWVMMIACVVVFIAMQI**LGDQEVMLWLAWPFDPTLKFEFWR**NN**THA**N**MHFS**LMHILFNLL**SGSGYPYDVPDYAFSTPVWISQHAPIRGSPGSSDKQEGEWPTGLRLSRIGGIH 186

MANMFALILVIATLVTGILWCVDKFFFAPKRRERQAAAQAAAGDSLDKATLKKVAPKPDPRYLAASWQAGHTGSGLHYRRYPFFAALRERAGP**VTWVMMIACVVVFIAMQI**LGDQEVMLWLAWPFDPTLKFEFWR**NN**THA**N**MHFS**LMHILFNL**SGSGYPYDVPDYAFSTPVWISQHAPIRGSPGSSDKQEGEWPTGLRLSRIGGIH 185

MANMFALILVIATLVTGILWCVDKFFFAPKRRERQAAAQAAAGDSLDKATLKKVAPKPDPRYLAASWQAGHTGSGLHYRRYPFFAALRERAGP**VTWVMMIACVVVFIAMQI**LGDQEVMLWLAWPFDPTLKFEFWR**NN**THA**N**MHFS**LMHILFN**SGSGYPYDVPDYAFSTPVWISQHAPIRGSPGSSDKQEGEWPTGLRLSRIGGIH 184

MANMFALILVIATLVTGILWCVDKFFFAPKRRERQAAAQAAAGDSLDKATLKKVAPKPDPRYLAASWQAGHTGSGLHYRRYPFFAALRERAGP**VTWVMMIACVVVFIAMQI**LGDQEVMLWLAWPFDPTLKFEFWR**NN**THA**N**MHFS**LMHILF**SGSGYPYDVPDYAFSTPVWISQHAPIRGSPGSSDKQEGEWPTGLRLSRIGGIH 183

MANMFALILVIATLVTGILWCVDKFFFAPKRRERQAAAQAAAGDSLDKATLKKVAPKPDPRYLAASWQAGHTGSGLHYRRYPFFAALRERAGP**VTWVMMIACVVVFIAMQI**LGDQEVMLWLAWPFDPTLKFEFWR**NN**THA**N**MHFS**LMHIL**SGSGYPYDVPDYAFSTPVWISQHAPIRGSPGSSDKQEGEWPTGLRLSRIGGIH 182

GlpG SecM(*Ec*) variant F^16^E (*N*=51-126)

MLMITSFANPRVAQA**E**VDYMATQGVILTIQQHNQSDVWLADESQAERVRADVARFLENPADPRYLAASWQAGHTGSGLHYRRYPFFAALRERAGP**V**SGSGYPYDVPDYAFSTPVWISQAQGIRAGPGSSDKQEGEWPTGLRLSRIGGIH  **126**

MLMITSFANPRVAQA**E**VDYMATQGVILTIQQHNQSDVWLADESQAERVRADVARFLENPADPRYLAASWQAGHTGSGLHYRRYPFFAALRESGSGYPYDVPDYAFSTPVWISQAQGIRAGPGSSDKQEGEWPTGLRLSRIGGIH  **121**

MLMITSFANPRVAQA**E**VDYMATQGVILTIQQHNQSDVWLADESQAERVRADVARFLENPADPRYLAASWQAGHTGSGLHYRRYPFFSGSGYPYDVPDYAFSTPVWISQAQGIRAGPGSSDKQEGEWPTGLRLSRIGGIH  **116**

MLMITSFANPRVAQA**E**VDYMATQGVILTIQQHNQSDVWLADESQAERVRADVARFLENPADPRYLAASWQAGHTGSGLHYRSGSGYPYDVPDYAFSTPVWISQAQGIRAGPGSSDKQEGEWPTGLRLSRIGGIH  **111**

MLMITSFANPRVAQA**E**VDYMATQGVILTIQQHNQSDVWLADESQAERVRADVARFLENPADPRYLAASWQAGHTGSSGSGYPYDVPDYAFSTPVWISQAQGIRAGPGSSDKQEGEWPTGLRLSRIGGIH  **106**

MLMITSFANPRVAQA**E**VDYMATQGVILTIQQHNQSDVWLADESQAERVRADVARFLENPADPRYLAASWQASGSGYPYDVPDYAFSTPVWISQAQGIRAGPGSSDKQEGEWPTGLRLSRIGGIH  **101**

MLMITSFANPRVAQA**E**VDYMATQGVILTIQQHNQSDVWLADESQAERVRADVARFLENPADPRYLASGSGYPYDVPDYAFSTPVWISQAQGIRAGPGSSDKQEGEWPTGLRLSRIGGIH  **96**

MLMITSFANPRVAQA**E**VDYMATQGVILTIQQHNQSDVWLADESQAERVRADVARFLENPADSGSGYPYDVPDYAFSTPVWISQAQGIRAGPGSSDKQEGEWPTGLRLSRIGGIH  **91**

MLMITSFANPRVAQA**E**VDYMATQGVILTIQQHNQSDVWLADESQAERVRADVARFLSGSGYPYDVPDYAFSTPVWISQAQGIRAGPGSSDKQEGEWPTGLRLSRIGGIH  **86**

MLMITSFANPRVAQA**E**VDYMATQGVILTIQQHNQSDVWLADESQAERVRADSGSGYPYDVPDYAFSTPVWISQAQGIRAGPGSSDKQEGEWPTGLRLSRIGGIH  **81**

MLMITSFANPRVAQA**E**VDYMATQGVILTIQQHNQSDVWLADESQAESGSGYPYDVPDYAFSTPVWISQAQGIRAGPGSSDKQEGEWPTGLRLSRIGGIH  **76**

MLMITSFANPRVAQA**E**VDYMATQGVILTIQQHNQSDVWLADSGSGYPYDVPDYAFSTPVWISQAQGIRAGPGSSDKQEGEWPTGLRLSRIGGIH  **71**

MLMITSFANPRVAQA**E**VDYMATQGVILTIQQHNQSDSGSGYPYDVPDYAFSTPVWISQAQGIRAGPGSSDKQEGEWPTGLRLSRIGGIH  **66**

MLMITSFANPRVAQA**E**VDYMATQGVILTIQQSGSGYPYDVPDYAFSTPVWISQAQGIRAGPGSSDKQEGEWPTGLRLSRIGGIH  **61**

MLMITSFANPRVAQA**E**VDYMATQGVISGSGYPYDVPDYAFSTPVWISQAQGIRAGPGSSDKQEGEWPTGLRLSRIGGIH  **56**

MLMITSFANPRVAQA**E**VDYMASGSGYPYDVPDYAFSTPVWISQAQGIRAGPGSSDKQEGEWPTGLRLSRIGGIH  **51**

*In vitro* expression: GlpG SecM(*Ec*) (*N*=71-126)

MLMITSFANPRVAQAFVDYMATQGVILTIQQHNQSDVWLADESQAERVRADVARFLENPADPRYLAASWQAGHTGSGLHYRRYPFFAALRERAGP**V**SGSGYPYDVPDYAFSTPVWISQAQGIRAGPGSSDKQEGEWPTGLRLSRIGGIH  **126**

MLMITSFANPRVAQAFVDYMATQGVILTIQQHNQSDVWLADESQAERVRADVARFLENPADPRYLAASWQAGHTGSGLHYRRYPFFAALRESGSGYPYDVPDYAFSTPVWISQAQGIRAGPGSSDKQEGEWPTGLRLSRIGGIH **121**

MLMITSFANPRVAQAFVDYMATQGVILTIQQHNQSDVWLADESQAERVRADVARFLENPADPRYLAASWQAGHTGSGLHYRRYPFFSGSGYPYDVPDYAFSTPVWISQAQGIRAGPGSSDKQEGEWPTGLRLSRIGGIH **116**

MLMITSFANPRVAQAFVDYMATQGVILTIQQHNQSDVWLADESQAERVRADVARFLENPADPRYLAASWQAGHTGSGLHYRSGSGYPYDVPDYAFSTPVWISQAQGIRAGPGSSDKQEGEWPTGLRLSRIGGIH  **111**

MLMITSFANPRVAQAFVDYMATQGVILTIQQHNQSDVWLADESQAERVRADVARFLENPADPRYLAASWQAGHTGSSGSGYPYDVPDYAFSTPVWISQAQGIRAGPGSSDKQEGEWPTGLRLSRIGGIH **106**

MLMITSFANPRVAQAFVDYMATQGVILTIQQHNQSDVWLADESQAERVRADVARFLENPADPRYLAASWQASGSGYPYDVPDYAFSTPVWISQAQGIRAGPGSSDKQEGEWPTGLRLSRIGGIH  **101**

MLMITSFANPRVAQAFVDYMATQGVILTIQQHNQSDVWLADESQAERVRADVARFLENPADPRYLASGSGYPYDVPDYAFSTPVWISQAQGIRAGPGSSDKQEGEWPTGLRLSRIGGIH  **96**

MLMITSFANPRVAQAFVDYMATQGVILTIQQHNQSDVWLADESQAERVRADVARFLENPADSGSGYPYDVPDYAFSTPVWISQAQGIRAGPGSSDKQEGEWPTGLRLSRIGGIH  **91**

MLMITSFANPRVAQAFVDYMATQGVILTIQQHNQSDVWLADESQAERVRADVARFLSGSGYPYDVPDYAFSTPVWISQAQGIRAGPGSSDKQEGEWPTGLRLSRIGGIH  **86**

MLMITSFANPRVAQAFVDYMATQGVILTIQQHNQSDVWLADESQAERVRADSGSGYPYDVPDYAFSTPVWISQAQGIRAGPGSSDKQEGEWPTGLRLSRIGGIH  **81**

MLMITSFANPRVAQAFVDYMATQGVILTIQQHNQSDVWLADESQAESGSGYPYDVPDYAFSTPVWISQAQGIRAGPGSSDKQEGEWPTGLRLSRIGGIH  **76**

MLMITSFANPRVAQAFVDYMATQGVILTIQQHNQSDVWLADSGSGYPYDVPDYAFSTPVWISQAQGIRAGPGSSDKQEGEWPTGLRLSRIGGIH  **71**

*In vitro* expression: GlpG SecM(*Ec*) variant F^16^E (*N*=71-126)

MLMITSFANPRVAQA**E**VDYMATQGVILTIQQHNQSDVWLADESQAERVRADVARFLENPADPRYLAASWQAGHTGSGLHYRRYPFFAALRERAGP**V**SGSGYPYDVPDYAFSTPVWISQAQGIRAGPGSSDKQEGEWPTGLRLSRIGGIH  **126**

MLMITSFANPRVAQA**E**VDYMATQGVILTIQQHNQSDVWLADESQAERVRADVARFLENPADPRYLAASWQAGHTGSGLHYRRYPFFAALRESGSGYPYDVPDYAFSTPVWISQAQGIRAGPGSSDKQEGEWPTGLRLSRIGGIH  **121**

MLMITSFANPRVAQA**E**VDYMATQGVILTIQQHNQSDVWLADESQAERVRADVARFLENPADPRYLAASWQAGHTGSGLHYRRYPFFSGSGYPYDVPDYAFSTPVWISQAQGIRAGPGSSDKQEGEWPTGLRLSRIGGIH  **116**

MLMITSFANPRVAQA**E**VDYMATQGVILTIQQHNQSDVWLADESQAERVRADVARFLENPADPRYLAASWQAGHTGSGLHYRSGSGYPYDVPDYAFSTPVWISQAQGIRAGPGSSDKQEGEWPTGLRLSRIGGIH  **111**

MLMITSFANPRVAQA**E**VDYMATQGVILTIQQHNQSDVWLADESQAERVRADVARFLENPADPRYLAASWQAGHTGSSGSGYPYDVPDYAFSTPVWISQAQGIRAGPGSSDKQEGEWPTGLRLSRIGGIH  **106**

MLMITSFANPRVAQA**E**VDYMATQGVILTIQQHNQSDVWLADESQAERVRADVARFLENPADPRYLAASWQASGSGYPYDVPDYAFSTPVWISQAQGIRAGPGSSDKQEGEWPTGLRLSRIGGIH  **101**

MLMITSFANPRVAQA**E**VDYMATQGVILTIQQHNQSDVWLADESQAERVRADVARFLENPADPRYLASGSGYPYDVPDYAFSTPVWISQAQGIRAGPGSSDKQEGEWPTGLRLSRIGGIH  **96**

MLMITSFANPRVAQA**E**VDYMATQGVILTIQQHNQSDVWLADESQAERVRADVARFLENPADSGSGYPYDVPDYAFSTPVWISQAQGIRAGPGSSDKQEGEWPTGLRLSRIGGIH  **91**

MLMITSFANPRVAQA**E**VDYMATQGVILTIQQHNQSDVWLADESQAERVRADVARFLSGSGYPYDVPDYAFSTPVWISQAQGIRAGPGSSDKQEGEWPTGLRLSRIGGIH  **86**

MLMITSFANPRVAQA**E**VDYMATQGVILTIQQHNQSDVWLADESQAERVRADSGSGYPYDVPDYAFSTPVWISQAQGIRAGPGSSDKQEGEWPTGLRLSRIGGIH  **81**

MLMITSFANPRVAQA**E**VDYMATQGVILTIQQHNQSDVWLADESQAESGSGYPYDVPDYAFSTPVWISQAQGIRAGPGSSDKQEGEWPTGLRLSRIGGIH  **76**

MLMITSFANPRVAQA**E**VDYMATQGVILTIQQHNQSDVWLADSGSGYPYDVPDYAFSTPVWISQAQGIRAGPGSSDKQEGEWPTGLRLSRIGGIH  **71**

*LepB TMH1* BtuC SecM(*Ec*)(N=43-386)

*N*

MLTLARQQQRQNIRWLLGPGGVWMSSFSTPVWISQAQGIRAGPGSSDKQEGEWPTGLRLSRIGGIH 43

MLTLARQQQRQNIRWLLCLSVGPGGVWMSSFSTPVWISQAQGIRAGPGSSDKQEGEWPTGLRLSRIGGIH 47

MLTLARQQQRQNIRWLLCLSVLMLLGPGGVWMSSFSTPVWISQAQGIRAGPGSSDKQEGEWPTGLRLSRIGGIH 51

MLTLARQQQRQNIRWLLCLSVLMLLALLLSLCAGPGGVWMSSFSTPVWISQAQGIRAGPGSSDKQEGEWPTGLRLSRIGGIH 59

MLTLARQQQRQNIRWLLCLSVLMLLALLLSLCAGEQWGPGGVWMSSFSTPVWISQAQGIRAGPGSSDKQEGEWPTGLRLSRIGGIH 63

MLTLARQQQRQNIRWLLCLSVLMLLALLLSLCAGEQWISPGGPGGVWMSSFSTPVWISQAQGIRAGPGSSDKQEGEWPTGLRLSRIGGIH 67

MLTLARQQQRQNIRWLLCLSVLMLLALLLSLCAGEQWISPGDWFTGPGGVWMSSFSTPVWISQAQGIRAGPGSSDKQEGEWPTGLRLSRIGGIH 71

MLTLARQQQRQNIRWLLCLSVLMLLALLLSLCAGEQWISPGDWFTPRGEGPGGVWMSSFSTPVWISQAQGIRAGPGSSDKQEGEWPTGLRLSRIGGIH 75

MLTLARQQQRQNIRWLLCLSVLMLLALLLSLCAGEQWISPGDWFTPRGELFVWGPGGVWMSSFSTPVWISQAQGIRAGPGSSDKQEGEWPTGLRLSRIGGIH 79

MLTLARQQQRQNIRWLLCLSVLMLLALLLSLCAGEQWISPGDWFTPRGELFVWQIRLGPGGVWMSSFSTPVWISQAQGIRAGPGSSDKQEGEWPTGLRLSRIGGIH 83

MLTLARQQQRQNIRWLLCLSVLMLLALLLSLCAGEQWISPGDWFTPRGELFVWQIRLPRTLGPGGVWMSSFSTPVWISQAQGIRAGPGSSDKQEGEWPTGLRLSRIGGIH 87

MLTLARQQQRQNIRWLLCLSVLMLLALLLSLCAGEQWISPGDWFTPRGELFVWQIRLPRTLAVLLGPGGVWMSSFSTPVWISQAQGIRAGPGSSDKQEGEWPTGLRLSRIGGIH 91

MLTLARQQQRQNIRWLLCLSVLMLLALLLSLCAGEQWISPGDWFTPRGELFVWQIRLPRTLAVLLVGAAGPGGVWMSSFSTPVWISQAQGIRAGPGSSDKQEGEWPTGLRLSRIGGIH 95

MLTLARQQQRQNIRWLLCLSVLMLLALLLSLCAGEQWISPGDWFTPRGELFVWQIRLPRTLAVLLVGAALAISGPGGVWMSSFSTPVWISQAQGIRAGPGSSDKQEGEWPTGLRLSRIGGIH 99

MLTLARQQQRQNIRWLLCLSVLMLLALLLSLCAGEQWISPGDWFTPRGELFVWQIRLPRTLAVLLVGAALAISGAVMGPGGVWMSSFSTPVWISQAQGIRAGPGSSDKQEGEWPTGLRLSRIGGIH 103

MLTLARQQQRQNIRWLLCLSVLMLLALLLSLCAGEQWISPGDWFTPRGELFVWQIRLPRTLAVLLVGAALAISGAVMQALFGPGGVWMSSFSTPVWISQAQGIRAGPGSSDKQEGEWPTGLRLSRIGGIH 107

MLTLARQQQRQNIRWLLCLSVLMLLALLLSLCAGEQWISPGDWFTPRGELFVWQIRLPRTLAVLLVGAALAISGAVMQALFENPLGPGGVWMSSFSTPVWISQAQGIRAGPGSSDKQEGEWPTGLRLSRIGGIH 111

MLTLARQQQRQNIRWLLCLSVLMLLALLLSLCAGEQWISPGDWFTPRGELFVWQIRLPRTLAVLLVGAALAISGAVMQALFENPLAEPGGPGGVWMSSFSTPVWISQAQGIRAGPGSSDKQEGEWPTGLRLSRIGGIH 115

MLTLARQQQRQNIRWLLCLSVLMLLALLLSLCAGEQWISPGDWFTPRGELFVWQIRLPRTLAVLLVGAALAISGAVMQALFENPLAEPGLLGVGPGGVWMSSFSTPVWISQAQGIRAGPGSSDKQEGEWPTGLRLSRIGGIH 119

MLTLARQQQRQNIRWLLCLSVLMLLALLLSLCAGEQWISPGDWFTPRGELFVWQIRLPRTLAVLLVGAALAISGAVMQALFENPLAEPGLLGVSNGAGPGGVWMSSFSTPVWISQAQGIRAGPGSSDKQEGEWPTGLRLSRIGGIH 123

MLTLARQQQRQNIRWLLCLSVLMLLALLLSLCAGEQWISPGDWFTPRGELFVWQIRLPRTLAVLLVGAALAISGAVMQALFENPLAEPGLLGVSNGAGVGLGPGGVWMSSFSTPVWISQAQGIRAGPGSSDKQEGEWPTGLRLSRIGGIH 127

MLTLARQQQRQNIRWLLCLSVLMLLALLLSLCAGEQWISPGDWFTPRGELFVWQIRLPRTLAVLLVGAALAISGAVMQALFENPLAEPGLLGVSNGAGVGLIAAVGPGGVWMSSFSTPVWISQAQGIRAGPGSSDKQEGEWPTGLRLSRIGGIH 131

MLTLARQQQRQNIRWLLCLSVLMLLALLLSLCAGEQWISPGDWFTPRGELFVWQIRLPRTLAVLLVGAALAISGAVMQALFENPLAEPGLLGVSNGAGVGLIAAVLLGQGPGGVWMSSFSTPVWISQAQGIRAGPGSSDKQEGEWPTGLRLSRIGGIH 135

MLTLARQQQRQNIRWLLCLSVLMLLALLLSLCAGEQWISPGDWFTPRGELFVWQIRLPRTLAVLLVGAALAISGAVMQALFENPLAEPGLLGVSNGAGVGLIAAVLLGQGQLPGPGGVWMSSFSTPVWISQAQGIRAGPGSSDKQEGEWPTGLRLSRIGGIH 139

MLTLARQQQRQNIRWLLCLSVLMLLALLLSLCAGEQWISPGDWFTPRGELFVWQIRLPRTLAVLLVGAALAISGAVMQALFENPLAEPGLLGVSNGAGVGLIAAVLLGQGQLPNWALGPGGVWMSSFSTPVWISQAQGIRAGPGSSDKQEGEWPTGLRLSRIGGIH 143

MLTLARQQQRQNIRWLLCLSVLMLLALLLSLCAGEQWISPGDWFTPRGELFVWQIRLPRTLAVLLVGAALAISGAVMQALFENPLAEPGLLGVSNGAGVGLIAAVLLGQGQLPNWALGLCAGPGGVWMSSFSTPVWISQAQGIRAGPGSSDKQEGEWPTGLRLSRIGGIH 147

MLTLARQQQRQNIRWLLCLSVLMLLALLLSLCAGEQWISPGDWFTPRGELFVWQIRLPRTLAVLLVGAALAISGAVMQALFENPLAEPGLLGVSNGAGVGLIAAVLLGQGQLPNWALGLCAIAGAGPGGVWMSSFSTPVWISQAQGIRAGPGSSDKQEGEWPTGLRLSRIGGIH 151

MLTLARQQQRQNIRWLLCLSVLMLLALLLSLCAGEQWISPGDWFTPRGELFVWQIRLPRTLAVLLVGAALAISGAVMQALFENPLAEPGLLGVSNGAGVGLIAAVLLGQGQLPNWALGLCAIAGALIITGPGGVWMSSFSTPVWISQAQGIRAGPGSSDKQEGEWPTGLRLSRIGGIH 155

MLTLARQQQRQNIRWLLCLSVLMLLALLLSLCAGEQWISPGDWFTPRGELFVWQIRLPRTLAVLLVGAALAISGAVMQALFENPLAEPGLLGVSNGAGVGLIAAVLLGQGQLPNWALGLCAIAGALIITLILLGPGGVWMSSFSTPVWISQAQGIRAGPGSSDKQEGEWPTGLRLSRIGGIH 159

MLTLARQQQRQNIRWLLCLSVLMLLALLLSLCAGEQWISPGDWFTPRGELFVWQIRLPRTLAVLLVGAALAISGAVMQALFENPLAEPGLLGVSNGAGVGLIAAVLLGQGQLPNWALGLCAIAGALIITLILLRFARGPGGVWMSSFSTPVWISQAQGIRAGPGSSDKQEGEWPTGLRLSRIGGIH 163

MLTLARQQQRQNIRWLLCLSVLMLLALLLSLCAGEQWISPGDWFTPRGELFVWQIRLPRTLAVLLVGAALAISGAVMQALFENPLAEPGLLGVSNGAGVGLIAAVLLGQGQLPNWALGLCAIAGALIITLILLRFARRHLSGPGGVWMSSFSTPVWISQAQGIRAGPGSSDKQEGEWPTGLRLSRIGGIH 167

MLTLARQQQRQNIRWLLCLSVLMLLALLLSLCAGEQWISPGDWFTPRGELFVWQIRLPRTLAVLLVGAALAISGAVMQALFENPLAEPGLLGVSNGAGVGLIAAVLLGQGQLPNWALGLCAIAGALIITLILLRFARRHLSTSRLGPGGVWMSSFSTPVWISQAQGIRAGPGSSDKQEGEWPTGLRLSRIGGIH 171

MLTLARQQQRQNIRWLLCLSVLMLLALLLSLCAGEQWISPGDWFTPRGELFVWQIRLPRTLAVLLVGAALAISGAVMQALFENPLAEPGLLGVSNGAGVGLIAAVLLGQGQLPNWALGLCAIAGALIITLILLRFARRHLSTSRLLLAGGPGGVWMSSFSTPVWISQAQGIRAGPGSSDKQEGEWPTGLRLSRIGGIH 175

MLTLARQQQRQNIRWLLCLSVLMLLALLLSLCAGEQWISPGDWFTPRGELFVWQIRLPRTLAVLLVGAALAISGAVMQALFENPLAEPGLLGVSNGAGVGLIAAVLLGQGQLPNWALGLCAIAGALIITLILLRFARRHLSTSRLLLAGVALGGPGGVWMSSFSTPVWISQAQGIRAGPGSSDKQEGEWPTGLRLSRIGGIH 179

MLTLARQQQRQNIRWLLCLSVLMLLALLLSLCAGEQWISPGDWFTPRGELFVWQIRLPRTLAVLLVGAALAISGAVMQALFENPLAEPGLLGVSNGAGVGLIAAVLLGQGQLPNWALGLCAIAGALIITLILLRFARRHLSTSRLLLAGVALGIICSGPGGVWMSSFSTPVWISQAQGIRAGPGSSDKQEGEWPTGLRLSRIGGIH 183

MLTLARQQQRQNIRWLLCLSVLMLLALLLSLCAGEQWISPGDWFTPRGELFVWQIRLPRTLAVLLVGAALAISGAVMQALFENPLAEPGLLGVSNGAGVGLIAAVLLGQGQLPNWALGLCAIAGALIITLILLRFARRHLSTSRLLLAGVALGIICSALMTGPGGVWMSSFSTPVWISQAQGIRAGPGSSDKQEGEWPTGLRLSRIGGIH 187

MLTLARQQQRQNIRWLLCLSVLMLLALLLSLCAGEQWISPGDWFTPRGELFVWQIRLPRTLAVLLVGAALAISGAVMQALFENPLAEPGLLGVSNGAGVGLIAAVLLGQGQLPNWALGLCAIAGALIITLILLRFARRHLSTSRLLLAGVALGIICSALMTWAIYGPGGVWMSSFSTPVWISQAQGIRAGPGSSDKQEGEWPTGLRLSRIGGIH 191

MLTLARQQQRQNIRWLLCLSVLMLLALLLSLCAGEQWISPGDWFTPRGELFVWQIRLPRTLAVLLVGAALAISGAVMQALFENPLAEPGLLGVSNGAGVGLIAAVLLGQGQLPNWALGLCAIAGALIITLILLRFARRHLSTSRLLLAGVALGIICSALMTWAIYFSTSGPGGVWMSSFSTPVWISQAQGIRAGPGSSDKQEGEWPTGLRLSRIGGIH 195

MLTLARQQQRQNIRWLLCLSVLMLLALLLSLCAGEQWISPGDWFTPRGELFVWQIRLPRTLAVLLVGAALAISGAVMQALFENPLAEPGLLGVSNGAGVGLIAAVLLGQGQLPNWALGLCAIAGALIITLILLRFARRHLSTSRLLLAGVALGIICSALMTWAIYFSTSVDLRGPGGVWMSSFSTPVWISQAQGIRAGPGSSDKQEGEWPTGLRLSRIGGIH 199

MLTLARQQQRQNIRWLLCLSVLMLLALLLSLCAGEQWISPGDWFTPRGELFVWQIRLPRTLAVLLVGAALAISGAVMQALFENPLAEPGLLGVSNGAGVGLIAAVLLGQGQLPNWALGLCAIAGALIITLILLRFARRHLSTSRLLLAGVALGIICSALMTWAIYFSTSVDLRQLMYGPGGVWMSSFSTPVWISQAQGIRAGPGSSDKQEGEWPTGLRLSRIGGIH 203

MLTLARQQQRQNIRWLLCLSVLMLLALLLSLCAGEQWISPGDWFTPRGELFVWQIRLPRTLAVLLVGAALAISGAVMQALFENPLAEPGLLGVSNGAGVGLIAAVLLGQGQLPNWALGLCAIAGALIITLILLRFARRHLSTSRLLLAGVALGIICSALMTWAIYFSTSVDLRQLMYWMMGGPGGVWMSSFSTPVWISQAQGIRAGPGSSDKQEGEWPTGLRLSRIGGIH 207

MLTLARQQQRQNIRWLLCLSVLMLLALLLSLCAGEQWISPGDWFTPRGELFVWQIRLPRTLAVLLVGAALAISGAVMQALFENPLAEPGLLGVSNGAGVGLIAAVLLGQGQLPNWALGLCAIAGALIITLILLRFARRHLSTSRLLLAGVALGIICSALMTWAIYFSTSVDLRQLMYWMMGGFGGGPGGVWMSSFSTPVWISQAQGIRAGPGSSDKQEGEWPTGLRLSRIGGIH 211

MLTLARQQQRQNIRWLLCLSVLMLLALLLSLCAGEQWISPGDWFTPRGELFVWQIRLPRTLAVLLVGAALAISGAVMQALFENPLAEPGLLGVSNGAGVGLIAAVLLGQGQLPNWALGLCAIAGALIITLILLRFARRHLSTSRLLLAGVALGIICSALMTWAIYFSTSVDLRQLMYWMMGGFGGVDWRGPGGVWMSSFSTPVWISQAQGIRAGPGSSDKQEGEWPTGLRLSRIGGIH 215

MLTLARQQQRQNIRWLLCLSVLMLLALLLSLCAGEQWISPGDWFTPRGELFVWQIRLPRTLAVLLVGAALAISGAVMQALFENPLAEPGLLGVSNGAGVGLIAAVLLGQGQLPNWALGLCAIAGALIITLILLRFARRHLSTSRLLLAGVALGIICSALMTWAIYFSTSVDLRQLMYWMMGGFGGVDWRQSWLGPGGVWMSSFSTPVWISQAQGIRAGPGSSDKQEGEWPTGLRLSRIGGIH 219

MLTLARQQQRQNIRWLLCLSVLMLLALLLSLCAGEQWISPGDWFTPRGELFVWQIRLPRTLAVLLVGAALAISGAVMQALFENPLAEPGLLGVSNGAGVGLIAAVLLGQGQLPNWALGLCAIAGALIITLILLRFARRHLSTSRLLLAGVALGIICSALMTWAIYFSTSVDLRQLMYWMMGGFGGVDWRQSWLMLALGPGGVWMSSFSTPVWISQAQGIRAGPGSSDKQEGEWPTGLRLSRIGGIH 223

MLTLARQQQRQNIRWLLCLSVLMLLALLLSLCAGEQWISPGDWFTPRGELFVWQIRLPRTLAVLLVGAALAISGAVMQALFENPLAEPGLLGVSNGAGVGLIAAVLLGQGQLPNWALGLCAIAGALIITLILLRFARRHLSTSRLLLAGVALGIICSALMTWAIYFSTSVDLRQLMYWMMGGFGGVDWRQSWLMLALIPVLGPGGVWMSSFSTPVWISQAQGIRAGPGSSDKQEGEWPTGLRLSRIGGIH 227

MLTLARQQQRQNIRWLLCLSVLMLLALLLSLCAGEQWISPGDWFTPRGELFVWQIRLPRTLAVLLVGAALAISGAVMQALFENPLAEPGLLGVSNGAGVGLIAAVLLGQGQLPNWALGLCAIAGALIITLILLRFARRHLSTSRLLLAGVALGIICSALMTWAIYFSTSVDLRQLMYWMMGGFGGVDWRQSWLMLALIPVLLWICGPGGVWMSSFSTPVWISQAQGIRAGPGSSDKQEGEWPTGLRLSRIGGIH 231

MLTLARQQQRQNIRWLLCLSVLMLLALLLSLCAGEQWISPGDWFTPRGELFVWQIRLPRTLAVLLVGAALAISGAVMQALFENPLAEPGLLGVSNGAGVGLIAAVLLGQGQLPNWALGLCAIAGALIITLILLRFARRHLSTSRLLLAGVALGIICSALMTWAIYFSTSVDLRQLMYWMMGGFGGVDWRQSWLMLALIPVLLWICCQSRGPGGVWMSSFSTPVWISQAQGIRAGPGSSDKQEGEWPTGLRLSRIGGIH 235

MLTLARQQQRQNIRWLLCLSVLMLLALLLSLCAGEQWISPGDWFTPRGELFVWQIRLPRTLAVLLVGAALAISGAVMQALFENPLAEPGLLGVSNGAGVGLIAAVLLGQGQLPNWALGLCAIAGALIITLILLRFARRHLSTSRLLLAGVALGIICSALMTWAIYFSTSVDLRQLMYWMMGGFGGVDWRQSWLMLALIPVLLWICCQSRPMNMGPGGVWMSSFSTPVWISQAQGIRAGPGSSDKQEGEWPTGLRLSRIGGIH 239

MLTLARQQQRQNIRWLLCLSVLMLLALLLSLCAGEQWISPGDWFTPRGELFVWQIRLPRTLAVLLVGAALAISGAVMQALFENPLAEPGLLGVSNGAGVGLIAAVLLGQGQLPNWALGLCAIAGALIITLILLRFARRHLSTSRLLLAGVALGIICSALMTWAIYFSTSVDLRQLMYWMMGGFGGVDWRQSWLMLALIPVLLWICCQSRPMNMLALGGPGGVWMSSFSTPVWISQAQGIRAGPGSSDKQEGEWPTGLRLSRIGGIH 243

MLTLARQQQRQNIRWLLCLSVLMLLALLLSLCAGEQWISPGDWFTPRGELFVWQIRLPRTLAVLLVGAALAISGAVMQALFENPLAEPGLLGVSNGAGVGLIAAVLLGQGQLPNWALGLCAIAGALIITLILLRFARRHLSTSRLLLAGVALGIICSALMTWAIYFSTSVDLRQLMYWMMGGFGGVDWRQSWLMLALIPVLLWICCQSRPMNMLALGEISAGPGGVWMSSFSTPVWISQAQGIRAGPGSSDKQEGEWPTGLRLSRIGGIH 247

MLTLARQQQRQNIRWLLCLSVLMLLALLLSLCAGEQWISPGDWFTPRGELFVWQIRLPRTLAVLLVGAALAISGAVMQALFENPLAEPGLLGVSNGAGVGLIAAVLLGQGQLPNWALGLCAIAGALIITLILLRFARRHLSTSRLLLAGVALGIICSALMTWAIYFSTSVDLRQLMYWMMGGFGGVDWRQSWLMLALIPVLLWICCQSRPMNMLALGEISARQLGGPGGVWMSSFSTPVWISQAQGIRAGPGSSDKQEGEWPTGLRLSRIGGIH 251

MLTLARQQQRQNIRWLLCLSVLMLLALLLSLCAGEQWISPGDWFTPRGELFVWQIRLPRTLAVLLVGAALAISGAVMQALFENPLAEPGLLGVSNGAGVGLIAAVLLGQGQLPNWALGLCAIAGALIITLILLRFARRHLSTSRLLLAGVALGIICSALMTWAIYFSTSVDLRQLMYWMMGGFGGVDWRQSWLMLALIPVLLWICCQSRPMNMLALGEISARQLGLPLWGPGGVWMSSFSTPVWISQAQGIRAGPGSSDKQEGEWPTGLRLSRIGGIH 255

MLTLARQQQRQNIRWLLCLSVLMLLALLLSLCAGEQWISPGDWFTPRGELFVWQIRLPRTLAVLLVGAALAISGAVMQALFENPLAEPGLLGVSNGAGVGLIAAVLLGQGQLPNWALGLCAIAGALIITLILLRFARRHLSTSRLLLAGVALGIICSALMTWAIYFSTSVDLRQLMYWMMGGFGGVDWRQSWLMLALIPVLLWICCQSRPMNMLALGEISARQLGLPLWFWRNGPGGVWMSSFSTPVWISQAQGIRAGPGSSDKQEGEWPTGLRLSRIGGIH 259

MLTLARQQQRQNIRWLLCLSVLMLLALLLSLCAGEQWISPGDWFTPRGELFVWQIRLPRTLAVLLVGAALAISGAVMQALFENPLAEPGLLGVSNGAGVGLIAAVLLGQGQLPNWALGLCAIAGALIITLILLRFARRHLSTSRLLLAGVALGIICSALMTWAIYFSTSVDLRQLMYWMMGGFGGVDWRQSWLMLALIPVLLWICCQSRPMNMLALGEISARQLGLPLWFWRNVLVAGPGGVWMSSFSTPVWISQAQGIRAGPGSSDKQEGEWPTGLRLSRIGGIH 263

MLTLARQQQRQNIRWLLCLSVLMLLALLLSLCAGEQWISPGDWFTPRGELFVWQIRLPRTLAVLLVGAALAISGAVMQALFENPLAEPGLLGVSNGAGVGLIAAVLLGQGQLPNWALGLCAIAGALIITLILLRFARRHLSTSRLLLAGVALGIICSALMTWAIYFSTSVDLRQLMYWMMGGFGGVDWRQSWLMLALIPVLLWICCQSRPMNMLALGEISARQLGLPLWFWRNVLVAATGWGPGGVWMSSFSTPVWISQAQGIRAGPGSSDKQEGEWPTGLRLSRIGGIH 267

MLTLARQQQRQNIRWLLCLSVLMLLALLLSLCAGEQWISPGDWFTPRGELFVWQIRLPRTLAVLLVGAALAISGAVMQALFENPLAEPGLLGVSNGAGVGLIAAVLLGQGQLPNWALGLCAIAGALIITLILLRFARRHLSTSRLLLAGVALGIICSALMTWAIYFSTSVDLRQLMYWMMGGFGGVDWRQSWLMLALIPVLLWICCQSRPMNMLALGEISARQLGLPLWFWRNVLVAATGWMVGVSVGPGGVWMSSFSTPVWISQAQGIRAGPGSSDKQEGEWPTGLRLSRIGGIH 273

MLTLARQQQRQNIRWLLCLSVLMLLALLLSLCAGEQWISPGDWFTPRGELFVWQIRLPRTLAVLLVGAALAISGAVMQALFENPLAEPGLLGVSNGAGVGLIAAVLLGQGQLPNWALGLCAIAGALIITLILLRFARRHLSTSRLLLAGVALGIICSALMTWAIYFSTSVDLRQLMYWMMGGFGGVDWRQSWLMLALIPVLLWICCQSRPMNMLALGEISARQLGLPLWFWRNVLVAATGWMVGVSVALAGGPGGVWMSSFSTPVWISQAQGIRAGPGSSDKQEGEWPTGLRLSRIGGIH 277

MLTLARQQQRQNIRWLLCLSVLMLLALLLSLCAGEQWISPGDWFTPRGELFVWQIRLPRTLAVLLVGAALAISGAVMQALFENPLAEPGLLGVSNGAGVGLIAAVLLGQGQLPNWALGLCAIAGALIITLILLRFARRHLSTSRLLLAGVALGIICSALMTWAIYFSTSVDLRQLMYWMMGGFGGVDWRQSWLMLALIPVLLWICCQSRPMNMLALGEISARQLGLPLWFWRNVLVAATGWMVGVSVALAGAIGVGPGGVWMSSFSTPVWISQAQGIRAGPGSSDKQEGEWPTGLRLSRIGGIH 281

MLTLARQQQRQNIRWLLCLSVLMLLALLLSLCAGEQWISPGDWFTPRGELFVWQIRLPRTLAVLLVGAALAISGAVMQALFENPLAEPGLLGVSNGAGVGLIAAVLLGQGQLPNWALGLCAIAGALIITLILLRFARRHLSTSRLLLAGVALGIICSALMTWAIYFSTSVDLRQLMYWMMGGFGGVDWRQSWLMLALIPVLLWICCQSRPMNMLALGEISARQLGLPLWFWRNVLVAATGWMVGVSVALAGAIGFIGLVGPGGVWMSSFSTPVWISQAQGIRAGPGSSDKQEGEWPTGLRLSRIGGIH 285

MLTLARQQQRQNIRWLLCLSVLMLLALLLSLCAGEQWISPGDWFTPRGELFVWQIRLPRTLAVLLVGAALAISGAVMQALFENPLAEPGLLGVSNGAGVGLIAAVLLGQGQLPNWALGLCAIAGALIITLILLRFARRHLSTSRLLLAGVALGIICSALMTWAIYFSTSVDLRQLMYWMMGGFGGVDWRQSWLMLALIPVLLWICCQSRPMNMLALGEISARQLGLPLWFWRNVLVAATGWMVGVSVALAGAIGFIGLVIPHIGPGGVWMSSFSTPVWISQAQGIRAGPGSSDKQEGEWPTGLRLSRIGGIH 289

MLTLARQQQRQNIRWLLCLSVLMLLALLLSLCAGEQWISPGDWFTPRGELFVWQIRLPRTLAVLLVGAALAISGAVMQALFENPLAEPGLLGVSNGAGVGLIAAVLLGQGQLPNWALGLCAIAGALIITLILLRFARRHLSTSRLLLAGVALGIICSALMTWAIYFSTSVDLRQLMYWMMGGFGGVDWRQSWLMLALIPVLLWICCQSRPMNMLALGEISARQLGLPLWFWRNVLVAATGWMVGVSVALAGAIGFIGLVIPHILRLCGPGGVWMSSFSTPVWISQAQGIRAGPGSSDKQEGEWPTGLRLSRIGGIH 293

MLTLARQQQRQNIRWLLCLSVLMLLALLLSLCAGEQWISPGDWFTPRGELFVWQIRLPRTLAVLLVGAALAISGAVMQALFENPLAEPGLLGVSNGAGVGLIAAVLLGQGQLPNWALGLCAIAGALIITLILLRFARRHLSTSRLLLAGVALGIICSALMTWAIYFSTSVDLRQLMYWMMGGFGGVDWRQSWLMLALIPVLLWICCQSRPMNMLALGEISARQLGLPLWFWRNVLVAATGWMVGVSVALAGAIGFIGLVIPHILRLCGLTDHGPGGVWMSSFSTPVWISQAQGIRAGPGSDYIKRAVGLPGDKVTYDPVSKELTIQPGCSSGQACENALPVTYSNVEPSDGSSDKQEGEWPTGLRLSRIGGIH 298

MLTLARQQQRQNIRWLLCLSVLMLLALLLSLCAGEQWISPGDWFTPRGELFVWQIRLPRTLAVLLVGAALAISGAVMQALFENPLAEPGLLGVSNGAGVGLIAAVLLGQGQLPNWALGLCAIAGALIITLILLRFARRHLSTSRLLLAGVALGIICSALMTWAIYFSTSVDLRQLMYWMMGGFGGVDWRQSWLMLALIPVLLWICCQSRPMNMLALGEISARQLGLPLWFWRNVLVAATGWMVGVSVALAGAIGFIGLVIPHILRLCGLTDHRVLLGPGGVWMSSFSTPVWISQAQGIRAGPGSDYIKRAVGLPGDKVTYDPVSKELTIQPGCSSGQACENALPVTYSNVEPSDGSSDKQEGEWPTGLRLSRIGGIH 302

MLTLARQQQRQNIRWLLCLSVLMLLALLLSLCAGEQWISPGDWFTPRGELFVWQIRLPRTLAVLLVGAALAISGAVMQALFENPLAEPGLLGVSNGAGVGLIAAVLLGQGQLPNWALGLCAIAGALIITLILLRFARRHLSTSRLLLAGVALGIICSALMTWAIYFSTSVDLRQLMYWMMGGFGGVDWRQSWLMLALIPVLLWICCQSRPMNMLALGEISARQLGLPLWFWRNVLVAATGWMVGVSVALAGAIGFIGLVIPHILRLCGLTDHRVLLPGCAGPGGVWMSSFSTPVWISQAQGIRAGPGSDYIKRAVGLPGDKVTYDPVSKELTIQPGCSSGQACENALPVTYSNVEPSDGSSDKQEGEWPTGLRLSRIGGIH 306

MLTLARQQQRQNIRWLLCLSVLMLLALLLSLCAGEQWISPGDWFTPRGELFVWQIRLPRTLAVLLVGAALAISGAVMQALFENPLAEPGLLGVSNGAGVGLIAAVLLGQGQLPNWALGLCAIAGALIITLILLRFARRHLSTSRLLLAGVALGIICSALMTWAIYFSTSVDLRQLMYWMMGGFGGVDWRQSWLMLALIPVLLWICCQSRPMNMLALGEISARQLGLPLWFWRNVLVAATGWMVGVSVALAGAIGFIGLVIPHILRLCGLTDHRVLLPGCALAGAGPGGVWMSSFSTPVWISQAQGIRAGPGSDYIKRAVGLPGDKVTYDPVSKELTIQPGCSSGQACENALPVTYSNVEPSDGSSDKQEGEWPTGLRLSRIGGIH 310

MLTLARQQQRQNIRWLLCLSVLMLLALLLSLCAGEQWISPGDWFTPRGELFVWQIRLPRTLAVLLVGAALAISGAVMQALFENPLAEPGLLGVSNGAGVGLIAAVLLGQGQLPNWALGLCAIAGALIITLILLRFARRHLSTSRLLLAGVALGIICSALMTWAIYFSTSVDLRQLMYWMMGGFGGVDWRQSWLMLALIPVLLWICCQSRPMNMLALGEISARQLGLPLWFWRNVLVAATGWMVGVSVALAGAIGFIGLVIPHILRLCGLTDHRVLLPGCALAGASALLGPGGVWMSSFSTPVWISQAQGIRAGPGSDYIKRAVGLPGDKVTYDPVSKELTIQPGCSSGQACENALPVTYSNVEPSDGSSDKQEGEWPTGLRLSRIGGIH 314

MLTLARQQQRQNIRWLLCLSVLMLLALLLSLCAGEQWISPGDWFTPRGELFVWQIRLPRTLAVLLVGAALAISGAVMQALFENPLAEPGLLGVSNGAGVGLIAAVLLGQGQLPNWALGLCAIAGALIITLILLRFARRHLSTSRLLLAGVALGIICSALMTWAIYFSTSVDLRQLMYWMMGGFGGVDWRQSWLMLALIPVLLWICCQSRPMNMLALGEISARQLGLPLWFWRNVLVAATGWMVGVSVALAGAIGFIGLVIPHILRLCGLTDHRVLLPGCALAGASALLLADIGPGGVWMSSFSTPVWISQAQGIRAGPGSDYIKRAVGLPGDKVTYDPVSKELTIQPGCSSGQACENALPVTYSNVEPSDGSSDKQEGEWPTGLRLSRIGGIH 318

MLTLARQQQRQNIRWLLCLSVLMLLALLLSLCAGEQWISPGDWFTPRGELFVWQIRLPRTLAVLLVGAALAISGAVMQALFENPLAEPGLLGVSNGAGVGLIAAVLLGQGQLPNWALGLCAIAGALIITLILLRFARRHLSTSRLLLAGVALGIICSALMTWAIYFSTSVDLRQLMYWMMGGFGGVDWRQSWLMLALIPVLLWICCQSRPMNMLALGEISARQLGLPLWFWRNVLVAATGWMVGVSVALAGAIGFIGLVIPHILRLCGLTDHRVLLPGCALAGASALLLADIVARLGPGGVWMSSFSTPVWISQAQGIRAGPGSDYIKRAVGLPGDKVTYDPVSKELTIQPGCSSGQACENALPVTYSNVEPSDGSSDKQEGEWPTGLRLSRIGGIH 322

MLTLARQQQRQNIRWLLCLSVLMLLALLLSLCAGEQWISPGDWFTPRGELFVWQIRLPRTLAVLLVGAALAISGAVMQALFENPLAEPGLLGVSNGAGVGLIAAVLLGQGQLPNWALGLCAIAGALIITLILLRFARRHLSTSRLLLAGVALGIICSALMTWAIYFSTSVDLRQLMYWMMGGFGGVDWRQSWLMLALIPVLLWICCQSRPMNMLALGEISARQLGLPLWFWRNVLVAATGWMVGVSVALAGAIGFIGLVIPHILRLCGLTDHRVLLPGCALAGASALLLADIVARLALAAGPGGVWMSSFSTPVWISQAQGIRAGPGSDYIKRAVGLPGDKVTYDPVSKELTIQPGCSSGQACENALPVTYSNVEPSDGSSDKQEGEWPTGLRLSRIGGIH 326

MLTLARQQQRQNIRWLLCLSVLMLLALLLSLCAGEQWISPGDWFTPRGELFVWQIRLPRTLAVLLVGAALAISGAVMQALFENPLAEPGLLGVSNGAGVGLIAAVLLGQGQLPNWALGLCAIAGALIITLILLRFARRHLSTSRLLLAGVALGIICSALMTWAIYFSTSVDLRQLMYWMMGGFGGVDWRQSWLMLALIPVLLWICCQSRPMNMLALGEISARQLGLPLWFWRNVLVAATGWMVGVSVALAGAIGFIGLVIPHILRLCGLTDHRVLLPGCALAGASALLLADIVARLALAAAELPIGGPGGVWMSSFSTPVWISQAQGIRAGPGSDYIKRAVGLPGDKVTYDPVSKELTIQPGCSSGQACENALPVTYSNVEPSDGSSDKQEGEWPTGLRLSRIGGIH 332

MLTLARQQQRQNIRWLLCLSVLMLLALLLSLCAGEQWISPGDWFTPRGELFVWQIRLPRTLAVLLVGAALAISGAVMQALFENPLAEPGLLGVSNGAGVGLIAAVLLGQGQLPNWALGLCAIAGALIITLILLRFARRHLSTSRLLLAGVALGIICSALMTWAIYFSTSVDLRQLMYWMMGGFGGVDWRQSWLMLALIPVLLWICCQSRPMNMLALGEISARQLGLPLWFWRNVLVAATGWMVGVSVALAGAIGFIGLVIPHILRLCGLTDHRVLLPGCALAGASALLLADIVARLALAAAELPIGVVTAGPGGVWMSSFSTPVWISQAQGIRAGPGSDYIKRAVGLPGDKVTYDPVSKELTIQPGCSSGQACENALPVTYSNVEPSDGSSDKQEGEWPTGLRLSRIGGIH 336

MLTLARQQQRQNIRWLLCLSVLMLLALLLSLCAGEQWISPGDWFTPRGELFVWQIRLPRTLAVLLVGAALAISGAVMQALFENPLAEPGLLGVSNGAGVGLIAAVLLGQGQLPNWALGLCAIAGALIITLILLRFARRHLSTSRLLLAGVALGIICSALMTWAIYFSTSVDLRQLMYWMMGGFGGVDWRQSWLMLALIPVLLWICCQSRPMNMLALGEISARQLGLPLWFWRNVLVAATGWMVGVSVALAGAIGFIGLVIPHILRLCGLTDHRVLLPGCALAGASALLLADIVARLALAAAELPIGVVTATLGAGPGGVWMSSFSTPVWISQAQGIRAGPGSDYIKRAVGLPGDKVTYDPVSKELTIQPGCSSGQACENALPVTYSNVEPSDGSSDKQEGEWPTGLRLSRIGGIH 340

MLTLARQQQRQNIRWLLCLSVLMLLALLLSLCAGEQWISPGDWFTPRGELFVWQIRLPRTLAVLLVGAALAISGAVMQALFENPLAEPGLLGVSNGAGVGLIAAVLLGQGQLPNWALGLCAIAGALIITLILLRFARRHLSTSRLLLAGVALGIICSALMTWAIYFSTSVDLRQLMYWMMGGFGGVDWRQSWLMLALIPVLLWICCQSRPMNMLALGEISARQLGLPLWFWRNVLVAATGWMVGVSVALAGAIGFIGLVIPHILRLCGLTDHRVLLPGCALAGASALLLADIVARLALAAAELPIGVVTATLGAPVFIGPGGVWMSSFSTPVWISQAQGIRAGPGSDYIKRAVGLPGDKVTYDPVSKELTIQPGCSSGQACENALPVTYSNVEPSDGSSDKQEGEWPTGLRLSRIGGIH 344

MLTLARQQQRQNIRWLLCLSVLMLLALLLSLCAGEQWISPGDWFTPRGELFVWQIRLPRTLAVLLVGAALAISGAVMQALFENPLAEPGLLGVSNGAGVGLIAAVLLGQGQLPNWALGLCAIAGALIITLILLRFARRHLSTSRLLLAGVALGIICSALMTWAIYFSTSVDLRQLMYWMMGGFGGVDWRQSWLMLALIPVLLWICCQSRPMNMLALGEISARQLGLPLWFWRNVLVAATGWMVGVSVALAGAIGFIGLVIPHILRLCGLTDHRVLLPGCALAGASALLLADIVARLALAAAELPIGVVTATLGAPVFIWLLLGPGGVWMSSFSTPVWISQAQGIRAGPGSDYIKRAVGLPGDKVTYDPVSKELTIQPGCSSGQACENALPVTYSNVEPSDGSSDKQEGEWPTGLRLSRIGGIH 348

MLTLARQQQRQNIRWLLCLSVLMLLALLLSLCAGEQWISPGDWFTPRGELFVWQIRLPRTLAVLLVGAALAISGAVMQALFENPLAEPGLLGVSNGAGVGLIAAVLLGQGQLPNWALGLCAIAGALIITLILLRFARRHLSTSRLLLAGVALGIICSALMTWAIYFSTSVDLRQLMYWMMGGFGGVDWRQSWLMLALIPVLLWICCQSRPMNMLALGEISARQLGLPLWFWRNVLVAATGWMVGVSVALAGAIGFIGLVIPHILRLCGLTDHRVLLPGCALAGASALLLADIVARLALAAAELPIGVVTATLGAPVFIWLLLKAGRGPGGVWMSSFSTPVWISQAQGIRAGPGSDYIKRAVGLPGDKVTYDPVSKELTIQPGCSSGQACENALPVTYSNVEPSDGSSDKQEGEWPTGLRLSRIGGIH 352

MLTLARQQQRQNIRWLLCLSVLMLLALLLSLCAGEQWISPGDWFTPRGELFVWQIRLPRTLAVLLVGAALAISGAVMQALFENPLAEPGLLGVSNGAGVGLIAAVLLGQGQLPNWALGLCAIAGALIITLILLRFARRHLSTSRLLLAGVALGIICSALMTWAIYFSTSVDLRQLMYWMMGGFGGVDWRQSWLMLALIPVLLWICCQSRPMNMLALGEISARQLGLPLWFWRNVLVAATGWMVGVSVALAGAIGFIGLVIPHILRLCGLTDHRVLLPGCALAGASALLLADIVARLALAAAELPIGVVTATLGAPVFIWLLLKAGRGPGGVPGWMSSFSTPVWISQAQGIRAGPGSDYIKRAVGLPGDKVTYDPVSKELTIQPGCSSGQACENALPVTYSNVEPSDGSSDKQEGEWPTGLRLSRIGGIH 354

MLTLARQQQRQNIRWLLCLSVLMLLALLLSLCAGEQWISPGDWFTPRGELFVWQIRLPRTLAVLLVGAALAISGAVMQALFENPLAEPGLLGVSNGAGVGLIAAVLLGQGQLPNWALGLCAIAGALIITLILLRFARRHLSTSRLLLAGVALGIICSALMTWAIYFSTSVDLRQLMYWMMGGFGGVDWRQSWLMLALIPVLLWICCQSRPMNMLALGEISARQLGLPLWFWRNVLVAATGWMVGVSVALAGAIGFIGLVIPHILRLCGLTDHRVLLPGCALAGASALLLADIVARLALAAAELPIGVVTATLGAPVFIWLLLKAGRGPGGVPGQQNAWMSSFSTPVWISQAQGIRAGPGSDYIKRAVGLPGDKVTYDPVSKELTIQPGCSSGQACENALPVTYSNVEPSDGSSDKQEGEWPTGLRLSRIGGIH 358

MLTLARQQQRQNIRWLLCLSVLMLLALLLSLCAGEQWISPGDWFTPRGELFVWQIRLPRTLAVLLVGAALAISGAVMQALFENPLAEPGLLGVSNGAGVGLIAAVLLGQGQLPNWALGLCAIAGALIITLILLRFARRHLSTSRLLLAGVALGIICSALMTWAIYFSTSVDLRQLMYWMMGGFGGVDWRQSWLMLALIPVLLWICCQSRPMNMLALGEISARQLGLPLWFWRNVLVAATGWMVGVSVALAGAIGFIGLVIPHILRLCGLTDHRVLLPGCALAGASALLLADIVARLALAAAELPIGVVTATLGAPVFIWLLLKAGRGPGGVPGQQNATWIVWMSSFSTPVWISQAQGIRAGPGSDYIKRAVGLPGDKVTYDPVSKELTIQPGCSSGQACENALPVTYSNVEPSDGSSDKQEGEWPTGLRLSRIGGIH 362

MLTLARQQQRQNIRWLLCLSVLMLLALLLSLCAGEQWISPGDWFTPRGELFVWQIRLPRTLAVLLVGAALAISGAVMQALFENPLAEPGLLGVSNGAGVGLIAAVLLGQGQLPNWALGLCAIAGALIITLILLRFARRHLSTSRLLLAGVALGIICSALMTWAIYFSTSVDLRQLMYWMMGGFGGVDWRQSWLMLALIPVLLWICCQSRPMNMLALGEISARQLGLPLWFWRNVLVAATGWMVGVSVALAGAIGFIGLVIPHILRLCGLTDHRVLLPGCALAGASALLLADIVARLALAAAELPIGVVTATLGAPVFIWLLLKAGRGPGGVPGQQNATWIVPPGQWMSSFSTPVWISQAQGIRAGPGSDYIKRAVGLPGDKVTYDPVSKELTIQPGCSSGQACENALPVTYSNVEPSDGSSDKQEGEWPTGLRLSRIGGIH 366

MLTLARQQQRQNIRWLLCLSVLMLLALLLSLCAGEQWISPGDWFTPRGELFVWQIRLPRTLAVLLVGAALAISGAVMQALFENPLAEPGLLGVSNGAGVGLIAAVLLGQGQLPNWALGLCAIAGALIITLILLRFARRHLSTSRLLLAGVALGIICSALMTWAIYFSTSVDLRQLMYWMMGGFGGVDWRQSWLMLALIPVLLWICCQSRPMNMLALGEISARQLGLPLWFWRNVLVAATGWMVGVSVALAGAIGFIGLVIPHILRLCGLTDHRVLLPGCALAGASALLLADIVARLALAAAELPIGVVTATLGAPVFIWLLLKAGRGPGGVPGQQNATWIVPPGQYFMMWMSSFSTPVWISQAQGIRAGPGSDYIKRAVGLPGDKVTYDPVSKELTIQPGCSSGQACENALPVTYSNVEPSDGSSDKQEGEWPTGLRLSRIGGIH 370

MLTLARQQQRQNIRWLLCLSVLMLLALLLSLCAGEQWISPGDWFTPRGELFVWQIRLPRTLAVLLVGAALAISGAVMQALFENPLAEPGLLGVSNGAGVGLIAAVLLGQGQLPNWALGLCAIAGALIITLILLRFARRHLSTSRLLLAGVALGIICSALMTWAIYFSTSVDLRQLMYWMMGGFGGVDWRQSWLMLALIPVLLWICCQSRPMNMLALGEISARQLGLPLWFWRNVLVAATGWMVGVSVALAGAIGFIGLVIPHILRLCGLTDHRVLLPGCALAGASALLLADIVARLALAAAELPIGVVTATLGAPVFIWLLLKAGRGPGGVPGQQNATWIVPPGQYFMMGDNRWMSSFSTPVWISQAQGIRAGPGSDYIKRAVGLPGDKVTYDPVSKELTIQPGCSSGQACENALPVTYSNVEPSDGSSDKQEGEWPTGLRLSRIGGIH 374

MLTLARQQQRQNIRWLLCLSVLMLLALLLSLCAGEQWISPGDWFTPRGELFVWQIRLPRTLAVLLVGAALAISGAVMQALFENPLAEPGLLGVSNGAGVGLIAAVLLGQGQLPNWALGLCAIAGALIITLILLRFARRHLSTSRLLLAGVALGIICSALMTWAIYFSTSVDLRQLMYWMMGGFGGVDWRQSWLMLALIPVLLWICCQSRPMNMLALGEISARQLGLPLWFWRNVLVAATGWMVGVSVALAGAIGFIGLVIPHILRLCGLTDHRVLLPGCALAGASALLLADIVARLALAAAELPIGVVTATLGAPVFIWLLLKAGRGPGGVPGQQNATWIVPPGQYFMMGDNRDNSAWMSSFSTPVWISQAQGIRAGPGSDYIKRAVGLPGDKVTYDPVSKELTIQPGCSSGQACENALPVTYSNVEPSDGSSDKQEGEWPTGLRLSRIGGIH 378

MLTLARQQQRQNIRWLLCLSVLMLLALLLSLCAGEQWISPGDWFTPRGELFVWQIRLPRTLAVLLVGAALAISGAVMQALFENPLAEPGLLGVSNGAGVGLIAAVLLGQGQLPNWALGLCAIAGALIITLILLRFARRHLSTSRLLLAGVALGIICSALMTWAIYFSTSVDLRQLMYWMMGGFGGVDWRQSWLMLALIPVLLWICCQSRPMNMLALGEISARQLGLPLWFWRNVLVAATGWMVGVSVALAGAIGFIGLVIPHILRLCGLTDHRVLLPGCALAGASALLLADIVARLALAAAELPIGVVTATLGAPVFIWLLLKAGRGPGGVPGQQNATWIVPPGQYFMMGDNRDNSADSRYWMSSFSTPVWISQAQGIRAGPGSDYIKRAVGLPGDKVTYDPVSKELTIQPGCSSGQACENALPVTYSNVEPSDGSSDKQEGEWPTGLRLSRIGGIH 382

MLTLARQQQRQNIRWLLCLSVLMLLALLLSLCAGEQWISPGDWFTPRGELFVWQIRLPRTLAVLLVGAALAISGAVMQALFENPLAEPGLLGVSNGAGVGLIAAVLLGQGQLPNWALGLCAIAGALIITLILLRFARRHLSTSRLLLAGVALGIICSALMTWAIYFSTSVDLRQLMYWMMGGFGGVDWRQSWLMLALIPVLLWICCQSRPMNMLALGEISARQLGLPLWFWRNVLVAATGWMVGVSVALAGAIGFIGLVIPHILRLCGLTDHRVLLPGCALAGASALLLADIVARLALAAAELPIGVVTATLGAPVFIWLLLKAGRGPGGVPGQQNATWIVPPGQYFMMGDNRDNSADSRYWGFVWMSSFSTPVWISQAQGIRAGPGSDYIKRAVGLPGDKVTYDPVSKELTIQPGCSSGQACENALPVTYSNVEPSDGSSDKQEGEWPTGLRLSRIGGIH 386

Hydrophobic TMH segment

Linker sequence (LepB derived)

SecM(Ec) AP

*LepB TMH1* BtuC SecM(*Ec*) R^47^R^56^R^59^→QQQ

MLTLARQQQRQNIRWLLCLSVLMLLALLLSLCAGEQWISPGDWFTP**Q**GELFVWQI**Q**LP**Q**TLAVLLVGAALAISGPGGVWMSSFSTPVWISQAQGIRAGPGSSDKQEGEWPTGLRLSRIGGIH 99

MLTLARQQQRQNIRWLLCLSVLMLLALLLSLCAGEQWISPGDWFTP**Q**GELFVWQI**Q**LP**Q**TLAVLLVGAALAISGAVMQALFGPGGVWMSSFSTPVWISQAQGIRAGPGSSDKQEGEWPTGLRLSRIGGIH 107

MLTLARQQQRQNIRWLLCLSVLMLLALLLSLCAGEQWISPGDWFTP**Q**GELFVWQI**Q**LP**Q**TLAVLLVGAALAISGAVMQALFENPLGPGGVWMSSFSTPVWISQAQGIRAGPGSSDKQEGEWPTGLRLSRIGGIH 111

MLTLARQQQRQNIRWLLCLSVLMLLALLLSLCAGEQWISPGDWFTP**Q**GELFVWQI**Q**LP**Q**TLAVLLVGAALAISGAVMQALFENPLAEPGLLGVSNGAGPGGVWMSSFSTPVWISQAQGIRAGPGSSDKQEGEWPTGLRLSRIGGIH 123

MLTLARQQQRQNIRWLLCLSVLMLLALLLSLCAGEQWISPGDWFTP**Q**GELFVWQI**Q**LP**Q**TLAVLLVGAALAISGAVMQALFENPLAEPGLLGVSNGAGVGLIAAVLLGQGPGGVWMSSFSTPVWISQAQGIRAGPGSSDKQEGEWPTGLRLSRIGGIH 135

*LepB TMH1* BtuC SecM(*Ec*) Hydrophobic residues→A residues

MLTLARQQQRQNIRWLLCLSV**A**M**AA**A**AAA**SLCAGEQWISPGGPGGVWMSSFSTPVWISQAQGIRAGPGSSDKQEGEWPTGLRLSRIGGIH 67

MLTLARQQQRQNIRWLLCLSVLMLLALLLSLCAGEQWISPGDWFTPRGELFVWQIRLPRTLAV**AAA**GAALAISGAVMQALFENPLGPGGVWMSSFSTPVWISQAQGIRAGPGSSDKQEGEWPTGLRLSRIGGIH 111

MLTLARQQQRQNIRWLLCLSVLMLLALLLSLCAGEQWISPGDWFTPRGELFVWQIRLPRTLAVLLVGAALAISGA**AA**QA**A**FENPLAEPGLLGVSNGAGPGGVWMSSFSTPVWISQAQGIRAGPGSSDKQEGEWPTGLRLSRIGGIH 123

MLTLARQQQRQNIRWLLCLSVLMLLALLLSLCAGEQWISPGDWFTPRGELFVWQIRLPRTLAVLLVGAALAISGAVMQALFENPLAEPGLLGVSNGAG**A**G**AA**AAVLLGQGQLPNWALGPGGVWMSSFSTPVWISQAQGIRAGPGSSDKQEGEWPTGLRLSRIGGIH 143

...LALLLSLCAGEQWISPGDWFTPRGELFVWQIRLPRTLAVLLVGAALAISGAVMQALFENPLAEPGLLGVSNGAGVGLIAAVLLGQGQLPNWALGLCAIAGA**AAA**TLILLRFARRHLSGPGGVWMSSFSTPVWISQAQGIRAGPGSSDKQEGEWPTGLRLSRIGGIH 167

...ELFVWQIRLPRTLAVLLVGAALAISGAVMQALFENPLAEPGLLGVSNGAGVGLIAAVLLGQGQLPNWALGLCAIAGALIITLILLRFARRHLSTSR**AAA**AGVALGIICSALMTWAIYGPGGVWMSSFSTPVWISQAQGIRAGPGSSDKQEGEWPTGLRLSRIGGIH 191

...LPRTLAVLLVGAALAISGAVMQALFENPLAEPGLLGVSNGAGVGLIAAVLLGQGQLPNWALGLCAIAGALIITLILLRFARRHLSTSRLLLAGVA**A**G**AA**CSALMTWAIYFSTSVDLRGPGGVWMSSFSTPVWISQAQGIRAGPGSSDKQEGEWPTGLRLSRIGGIH 199

...LGLCAIAGALIITLILLRFARRHLSTSRLLLAGVALGIICSALMTWAIYFSTSVDLRQLMYWMMGGFGGVDWRQSWLMLALIPVLLWICCQSRPMN**AA**A**A**GEISARQLGLPLWFWRNGPGGVWMSSFSTPVWISQAQGIRAGPGSSDKQEGEWPTGLRLSRIGGIH 259

...LAGVALGIICSALMTWAIYFSTSVDLRQLMYWMMGGFGGVDWRQSWLMLALIPVLLWICCQSRPMNMLALGEISARQLGLPLWFWRNVLVAATGWM**A**G**A**S**A**ALAGAIGFIGLVIPHIGPGGVWMSSFSTPVWISQAQGIRAGPGSSDKQEGEWPTGLRLSRIGGIH 289

...SRPMNMLALGEISARQLGLPLWFWRNVLVAATGWMVGVSVALAGAIGFIG**AAA**PHILRLCGLTDHGPGGVWMSSFSTPVWISQAQGIRAGPGSDYIKRAVGLPGDKVTYDPVSKELTIQPGCSSGQACENALPVTYSNVEPSDGSSDKQEGEWPTGLRLSRIGGIH 298

...LGLPLWFWRNVLVAATGWMVGVSVALAGAIGFIGLVIPHILRLCGLTDHR**AAA**PGCALAGASALLGPGGVWMSSFSTPVWISQAQGIRAGPGSDYIKRAVGLPGDKVTYDPVSKELTIQPGCSSGQACENALPVTYSNVEPSDGSSDKQEGEWPTGLRLSRIGGIH 314

...LVIPHILRLCGLTDHRVLLPGCALAGASALLLADIVARLALAAAELP**A**G**AA**TATLGAPVFIWLLLGPGGVWMSSFSTPVWISQAQGIRAGPGSDYIKRAVGLPGDKVTYDPVSKELTIQPGCSSGQACENALPVTYSNVEPSDGSSDKQEGEWPTGLRLSRIGGIH 348

*N-terminal LepB sequence for BtuC wild type, R^47^R^56^R^59^→QQQ and hydrophobic residue mutants:*

*MANMFALILVIATLVTGILWCVDKFFFAPKRRERQAAAQAAAGDSLDKATLKKVAPKPPIYQKTLIETGHPKRGDIVVFKYPEDPKLDYIKRAVGLPGDKVTYDPVSKELTIQPGCSSGQACENALPVTYSNVEPSDFVQTFSRRNGGEATSGFFEVPKQETKENGIRLSETSGGPG*

LepB TMH1

Sequence between PP was deleted to remove TMH2 of LepB

Separation between LepB and BtuC

*LepB TMH1-TMH2* BtuC SecM(*Ec*) Single TMHs

TMH2

TLAVLLVGAALAISGAVMQALFGPSFSTPVWISQAQGIRAGPGSSDKQEGEWPTGLRLSRIGGIH 101*

TLAVLLVGAALAISGAVMQALFGPGGVPSFSTPVWISQAQGIRAGPGSSDKQEGEWPTGLRLSRIGGIH 105*

TLAVLLVGAALAISGAVMQALFGPGGVPGWMSSFSTPVWISQAQGIRAGPGSSDKQEGEWPTGLRLSRIGGIH 109*

TLAVLLVGAALAISGAVMQALFGPGGVPGQQNATWWMSSFSTPVWISQAQGIRAGPGSSDKQEGEWPTGLRLSRIGGIH 115*

TLAVLLVGAALAISGAVMQALFGPGGVPGQQNATWIVPPGQWMSSFSTPVWISQAQGIRAGPGSSDKQEGEWPTGLRLSRIGGIH 121*

TLAVLLVGAALAISGAVMQALFGPGGVPGQQNATWIVPPGQYFMMGDWMSSFSTPVWISQAQGIRAGPGSSDKQEGEWPTGLRLSRIGGIH 127*

TLAVLLVGAALAISGAVMQALFGPGGVPGQQNATWIVPPGQYFMMGDNRDNSAWMSSFSTPVWISQAQGIRAGPGSSDKQEGEWPTGLRLSRIGGIH 133*

TLAVLLVGAALAISGAVMQALFGPGGVPGQQNATWIVPPGQYFMMGDNRDNSADSRYWGWMSSFSTPVWISQAQGIRAGPGSSDKQEGEWPTGLRLSRIGGIH 139*

TLAVLLVGAALAISGAVMQALFGPGGVPGQQNATWIVPPGQYFMMGDNRDNSADSRYWGFVPEANWMSSFSTPVWISQAQGIRAGPGSSDKQEGEWPTGLRLSRIGGIH 145*

TMH6

DWRQSWLMLALIPVLLWICCGPSFSTPVWISQAQGIRAGPGSSDKQEGEWPTGLRLSRIGGIH 226*

DWRQSWLMLALIPVLLWICCGPGGVPSFSTPVWISQAQGIRAGPGSSDKQEGEWPTGLRLSRIGGIH 230*

DWRQSWLMLALIPVLLWICCGPGGVPGWMSSFSTPVWISQAQGIRAGPGSSDKQEGEWPTGLRLSRIGGIH 234*

DWRQSWLMLALIPVLLWICCGPGGVPGQQNATWWMSSFSTPVWISQAQGIRAGPGSSDKQEGEWPTGLRLSRIGGIH 240*

DWRQSWLMLALIPVLLWICCGPGGVPGQQNATWIVPPGQWMSSFSTPVWISQAQGIRAGPGSSDKQEGEWPTGLRLSRIGGIH 246*

DWRQSWLMLALIPVLLWICCGPGGVPGQQNATWIVPPGQYFMMGDWMSSFSTPVWISQAQGIRAGPGSSDKQEGEWPTGLRLSRIGGIH 252*

DWRQSWLMLALIPVLLWICCGPGGVPGQQNATWIVPPGQYFMMGDNRDNSAWMSSFSTPVWISQAQGIRAGPGSSDKQEGEWPTGLRLSRIGGIH 258*

DWRQSWLMLALIPVLLWICCGPGGVPGQQNATWIVPPGQYFMMGDNRDNSADSRYWGWMSSFSTPVWISQAQGIRAGPGSSDKQEGEWPTGLRLSRIGGIH 264*

DWRQSWLMLALIPVLLWICCGPGGVPGQQNATWIVPPGQYFMMGDNRDNSADSRYWGFVPEANWMSSFSTPVWISQAQGIRAGPGSSDKQEGEWPTGLRLSRIGGIH 270*

TMH8

AGAIGFIGLVIPHILRLCGPSFSTPVWISQAQGIRAGPGSSDKQEGEWPTGLRLSRIGGIH 287*

AGAIGFIGLVIPHILRLCGPGGVPSFSTPVWISQAQGIRAGPGSSDKQEGEWPTGLRLSRIGGIH 291*

AGAIGFIGLVIPHILRLCGPGGVWMSSFSTPVWISQAQGIRAGPGSSDKQEGEWPTGLRLSRIGGIH 293*

AGAIGFIGLVIPHILRLCGPGGVPGWMSSFSTPVWISQAQGIRAGPGSSDKQEGEWPTGLRLSRIGGIH 295*

AGAIGFIGLVIPHILRLCGPGGVPGQQWMSSFSTPVWISQAQGIRAGPGSSDKQEGEWPTGLRLSRIGGIH 297*

AGAIGFIGLVIPHILRLCGPGGVPGQQNATWWMSSFSTPVWISQAQGIRAGPGSSDKQEGEWPTGLRLSRIGGIH 301*

AGAIGFIGLVIPHILRLCGPGGVPGQQNATWIVPPGQWMSSFSTPVWISQAQGIRAGPGSSDKQEGEWPTGLRLSRIGGIH 307*

AGAIGFIGLVIPHILRLCGPGGVPGQQNATWIVPPGQYFWMSSFSTPVWISQAQGIRAGPGSSDKQEGEWPTGLRLSRIGGIH 309*

AGAIGFIGLVIPHILRLCGPGGVPGQQNATWIVPPGQYFMMGDWMSSFSTPVWISQAQGIRAGPGSSDKQEGEWPTGLRLSRIGGIH 313*

AGAIGFIGLVIPHILRLCGPGGVPGQQNATWIVPPGQYFMMGDNRDNSAWMSSFSTPVWISQAQGIRAGPGSSDKQEGEWPTGLRLSRIGGIH 319*

AGAIGFIGLVIPHILRLCGPGGVPGQQNATWIVPPGQYFMMGDNRDNSADSRYWMSSFSTPVWISQAQGIRAGPGSSDKQEGEWPTGLRLSRIGGIH 323*

AGAIGFIGLVIPHILRLCGPGGVPGQQNATWIVPPGQYFMMGDNRDNSADSRYWGWMSSFSTPVWISQAQGIRAGPGSSDKQEGEWPTGLRLSRIGGIH 325*

AGAIGFIGLVIPHILRLCGPGGVPGQQNATWIVPPGQYFMMGDNRDNSADSRYWGFVPEWMSSFSTPVWISQAQGIRAGPGSSDKQEGEWPTGLRLSRIGGIH 329*

TMH10

AAAELPIGVVTATLGAPVFIWLLLKAGRGPSFSTPVWISQAQGIRAGPGSSDKQEGEWPTGLRLSRIGGIH 346*

AAAELPIGVVTATLGAPVFIWLLLKAGRGPGGVPSFSTPVWISQAQGIRAGPGSSDKQEGEWPTGLRLSRIGGIH 350*

AAAELPIGVVTATLGAPVFIWLLLKAGRGPGGVPGWMSSFSTPVWISQAQGIRAGPGSSDKQEGEWPTGLRLSRIGGIH 354*

AAAELPIGVVTATLGAPVFIWLLLKAGRGPGGVPGQQNATWWMSSFSTPVWISQAQGIRAGPGSSDKQEGEWPTGLRLSRIGGIH 360*

AAAELPIGVVTATLGAPVFIWLLLKAGRGPGGVPGQQNATWIVPPGQWMSSFSTPVWISQAQGIRAGPGSSDKQEGEWPTGLRLSRIGGIH 366*

AAAELPIGVVTATLGAPVFIWLLLKAGRGPGGVPGQQNATWIVPPGQYFMMGDWMSSFSTPVWISQAQGIRAGPGSSDKQEGEWPTGLRLSRIGGIH 372*

AAAELPIGVVTATLGAPVFIWLLLKAGRGPGGVPGQQNATWIVPPGQYFMMGDNRDNSAWMSSFSTPVWISQAQGIRAGPGSSDKQEGEWPTGLRLSRIGGIH 378*

AAAELPIGVVTATLGAPVFIWLLLKAGRGPGGVPGQQNATWIVPPGQYFMMGDNRDNSADSRYWGWMSSFSTPVWISQAQGIRAGPGSSDKQEGEWPTGLRLSRIGGIH 384*

AAAELPIGVVTATLGAPVFIWLLLKAGRGPGGVPGQQNATWIVPPGQYFMMGDNRDNSADSRYWGFVPEANWMSSFSTPVWISQAQGIRAGPGSSDKQEGEWPTGLRLSRIGGIH 390*

*N-terminal LepB sequence for BtuC TMH2, TMH6, TMH8 and TMH10:*

*MANMFALILVIATLVTGILWCVDKFFFAPKRRERQAAAQAAAGDSLDKATLKKVAPKPGWLETGASVFPVLAIVLIVRSFIYEPFQIPSGSMMPTLNSTDFILVEKFAYGIKDPIYQKTLIETGHPKRGDIVVFKYPEDPKLDYIKRAVGLPGDKVTYDPVSKELTIQPGCSSGQACENALPVTYSNVEPSDFVQTFSRRNGGEATSGFFEVPKQETKENGIRLSETSGGPG*

LepB TMH1 and TMH2

LepB TMH2 between PP was reintroduced

*LepB TMH1* BtuC SecM(*Ec*) TMH5-6

RHLSTSRLLLAGVALGIICSALMTWAIYFSTSVDLRQLMYWMMGGFGGVDWRQSWLMLALIPVLLWICCGPSFSTPVWISQAQGIRAGPGSSDKQEGEWPTGLRLSRIGGIH 226*

RHLSTSRLLLAGVALGIICSALMTWAIYFSTSVDLRQLMYWMMGGFGGVDWRQSWLMLALIPVLLWICCGPGGVPSFSTPVWISQAQGIRAGPGSSDKQEGEWPTGLRLSRIGGIH 230*

RHLSTSRLLLAGVALGIICSALMTWAIYFSTSVDLRQLMYWMMGGFGGVDWRQSWLMLALIPVLLWICCGPGGVPGWMSSFSTPVWISQAQGIRAGPGSSDKQEGEWPTGLRLSRIGGIH 234*

RHLSTSRLLLAGVALGIICSALMTWAIYFSTSVDLRQLMYWMMGGFGGVDWRQSWLMLALIPVLLWICCGPGGVPGQQNATWWMSSFSTPVWISQAQGIRAGPGSSDKQEGEWPTGLRLSRIGGIH 240*

RHLSTSRLLLAGVALGIICSALMTWAIYFSTSVDLRQLMYWMMGGFGGVDWRQSWLMLALIPVLLWICCGPGGVPGQQNATWIVPPGQWMSSFSTPVWISQAQGIRAGPGSSDKQEGEWPTGLRLSRIGGIH 246*

RHLSTSRLLLAGVALGIICSALMTWAIYFSTSVDLRQLMYWMMGGFGGVDWRQSWLMLALIPVLLWICCGPGGVPGQQNATWIVPPGQYFMMGDWMSSFSTPVWISQAQGIRAGPGSSDKQEGEWPTGLRLSRIGGIH 252*

RHLSTSRLLLAGVALGIICSALMTWAIYFSTSVDLRQLMYWMMGGFGGVDWRQSWLMLALIPVLLWICCGPGGVPGQQNATWIVPPGQYFMMGDNRDNSAWMSSFSTPVWISQAQGIRAGPGSSDKQEGEWPTGLRLSRIGGIH 258*

RHLSTSRLLLAGVALGIICSALMTWAIYFSTSVDLRQLMYWMMGGFGGVDWRQSWLMLALIPVLLWICCGPGGVPGQQNATWIVPPGQYFMMGDNRDNSADSRYWGWMSSFSTPVWISQAQGIRAGPGSSDKQEGEWPTGLRLSRIGGIH 264*

RHLSTSRLLLAGVALGIICSALMTWAIYFSTSVDLRQLMYWMMGGFGGVDWRQSWLMLALIPVLLWICCGPGGVPGQQNATWIVPPGQYFMMGDNRDNSADSRYWGFVPEANWMSSFSTPVWISQAQGIRAGPGSSDKQEGEWPTGLRLSRIGGIH 270*

*LepB TMH1* BtuC SecM(*Ec*) ΔTMH1-4

RHLSTSRLLLAGVALGGPGGVWMSSFSTPVWISQAQGIRAGPGSSDKQEGEWPTGLRLSRIGGIH 179*

RHLSTSRLLLAGVALGIICSALMTWAIYGPGGVWMSSFSTPVWISQAQGIRAGPGSSDKQEGEWPTGLRLSRIGGIH 191*

RHLSTSRLLLAGVALGIICSALMTWAIYFSTSVDLRQLMYGPGGVWMSSFSTPVWISQAQGIRAGPGSSDKQEGEWPTGLRLSRIGGIH 203*

RHLSTSRLLLAGVALGIICSALMTWAIYFSTSVDLRQLMYWMMGGFGGVDWRGPGGVWMSSFSTPVWISQAQGIRAGPGSSDKQEGEWPTGLRLSRIGGIH 215*

RHLSTSRLLLAGVALGIICSALMTWAIYFSTSVDLRQLMYWMMGGFGGVDWRQSWLMLALIPVLGPGGVWMSSFSTPVWISQAQGIRAGPGSSDKQEGEWPTGLRLSRIGGIH 227*

RHLSTSRLLLAGVALGIICSALMTWAIYFSTSVDLRQLMYWMMGGFGGVDWRQSWLMLALIPVLLWICGPGGVWMSSFSTPVWISQAQGIRAGPGSSDKQEGEWPTGLRLSRIGGIH 231*

RHLSTSRLLLAGVALGIICSALMTWAIYFSTSVDLRQLMYWMMGGFGGVDWRQSWLMLALIPVLLWICCQSRPMNMLALGEISAGPGGVWMSSFSTPVWISQAQGIRAGPGSSDKQEGEWPTGLRLSRIGGIH 247*

RHLSTSRLLLAGVALGIICSALMTWAIYFSTSVDLRQLMYWMMGGFGGVDWRQSWLMLALIPVLLWICCQSRPMNMLALGEISARQLGLPLWFWRNGPGGVWMSSFSTPVWISQAQGIRAGPGSSDKQEGEWPTGLRLSRIGGIH 259*

RHLSTSRLLLAGVALGIICSALMTWAIYFSTSVDLRQLMYWMMGGFGGVDWRQSWLMLALIPVLLWICCQSRPMNMLALGEISARQLGLPLWFWRNVLVAATGWMVGVSVALAGGPGGVWMSSFSTPVWISQAQGIRAGPGSSDKQEGEWPTGLRLSRIGGIH 277*

RHLSTSRLLLAGVALGIICSALMTWAIYFSTSVDLRQLMYWMMGGFGGVDWRQSWLMLALIPVLLWICCQSRPMNMLALGEISARQLGLPLWFWRNVLVAATGWMVGVSVALAGAIGFIGLVIPHIGPGGVWMSSFSTPVWISQAQGIRAGPGSSDKQEGEWPTGLRLSRIGGIH 289*

...PVLLWICCQSRPMNMLALGEISARQLGLPLWFWRNVLVAATGWMVGVSVALAGAIGFIGLVIPHILRLCGLTDHGPGGVWMSSFSTPVWISQAQGIRAGPGSDYIKRAVGLPGDKVTYDPVSKELTIQPGCSSGQACENALPVTYSNVEPSDGSSDKQEGEWPTGLRLSRIGGIH 298*

...QSRPMNMLALGEISARQLGLPLWFWRNVLVAATGWMVGVSVALAGAIGFIGLVIPHILRLCGLTDHRVLLPGCAGPGGVWMSSFSTPVWISQAQGIRAGPGSDYIKRAVGLPGDKVTYDPVSKELTIQPGCSSGQACENALPVTYSNVEPSDGSSDKQEGEWPTGLRLSRIGGIH 306*

...ALGEISARQLGLPLWFWRNVLVAATGWMVGVSVALAGAIGFIGLVIPHILRLCGLTDHRVLLPGCALAGASALLGPGGVWMSSFSTPVWISQAQGIRAGPGSDYIKRAVGLPGDKVTYDPVSKELTIQPGCSSGQACENALPVTYSNVEPSDGSSDKQEGEWPTGLRLSRIGGIH 314*

...QLGLPLWFWRNVLVAATGWMVGVSVALAGAIGFIGLVIPHILRLCGLTDHRVLLPGCALAGASALLLADIVARLGPGGVWMSSFSTPVWISQAQGIRAGPGSDYIKRAVGLPGDKVTYDPVSKELTIQPGCSSGQACENALPVTYSNVEPSDGSSDKQEGEWPTGLRLSRIGGIH 322*

...AATGWMVGVSVALAGAIGFIGLVIPHILRLCGLTDHRVLLPGCALAGASALLLADIVARLALAAAELPIGVVTAGPGGVWMSSFSTPVWISQAQGIRAGPGSDYIKRAVGLPGDKVTYDPVSKELTIQPGCSSGQACENALPVTYSNVEPSDGSSDKQEGEWPTGLRLSRIGGIH 336*

...LAGAIGFIGLVIPHILRLCGLTDHRVLLPGCALAGASALLLADIVARLALAAAELPIGVVTATLGAPVFIWLLLGPGGVWMSSFSTPVWISQAQGIRAGPGSDYIKRAVGLPGDKVTYDPVSKELTIQPGCSSGQACENALPVTYSNVEPSDGSSDKQEGEWPTGLRLSRIGGIH 348*

...VIPHILRLCGLTDHRVLLPGCALAGASALLLADIVARLALAAAELPIGVVTATLGAPVFIWLLLKAGRGPGGVPGQQNAWMSSFSTPVWISQAQGIRAGPGSDYIKRAVGLPGDKVTYDPVSKELTIQPGCSSGQACENALPVTYSNVEPSDGSSDKQEGEWPTGLRLSRIGGIH 358*

...CGLTDHRVLLPGCALAGASALLLADIVARLALAAAELPIGVVTATLGAPVFIWLLLKAGRGPGGVPGQQNATWIVPPGQWMSSFSTPVWISQAQGIRAGPGSDYIKRAVGLPGDKVTYDPVSKELTIQPGCSSGQACENALPVTYSNVEPSDGSSDKQEGEWPTGLRLSRIGGIH 366*

...LLPGCALAGASALLLADIVARLALAAAELPIGVVTATLGAPVFIWLLLKAGRGPGGVPGQQNATWIVPPGQYFMMGDNRWMSSFSTPVWISQAQGIRAGPGSDYIKRAVGLPGDKVTYDPVSKELTIQPGCSSGQACENALPVTYSNVEPSDGSSDKQEGEWPTGLRLSRIGGIH 374*

...GASALLLADIVARLALAAAELPIGVVTATLGAPVFIWLLLKAGRGPGGVPGQQNATWIVPPGQYFMMGDNRDNSADSRYWMSSFSTPVWISQAQGIRAGPGSDYIKRAVGLPGDKVTYDPVSKELTIQPGCSSGQACENALPVTYSNVEPSDGSSDKQEGEWPTGLRLSRIGGIH 382*

*N-terminal LepB sequence for BtuC TMH5-6 and for BtuC ΔTMH1-4:*

*MANMFALILVIATLVTGILWCVDKFFFAPKRRERQAAAQAAAGDSLDKATLKKVAPKPPIYQKTLIETGHPKRGDIVVFKYPEDPKLDYIKRAVGLPGDKVTYDPVSKELTIQPGCSSGQACENALPVTYSNVEPSDFVQTFSRRNGGEATSGFFEVPKQETKENGIRLSETSGGPG*

*Deleted N-terminal sequence of BtuC needs to be added on

BtuC HA SecM(*Ec*)

MLTLARQQQRQNIRWLLCLSVLMLLYPYDVPDYAFSTPVWISQAQGIRAGPGSSDKQEGEWPTGLRLSRIGGIH 51

MLTLARQQQRQNIRWLLCLSVLMLLALLLSLCAGEQWISPGYPYDVPDYAFSTPVWISQAQGIRAGPGSSDKQEGEWPTGLRLSRIGGIH 67

MLTLARQQQRQNIRWLLCLSVLMLLALLLSLCAGEQWISPGDWFTPRGELFVWYPYDVPDYAFSTPVWISQAQGIRAGPGSSDKQEGEWPTGLRLSRIGGIH 79

MLTLARQQQRQNIRWLLCLSVLMLLALLLSLCAGEQWISPGDWFTPRGELFVWQIRLPRTLAVLLVGAALAISYPYDVPDYAFSTPVWISQAQGIRAGPGSSDKQEGEWPTGLRLSRIGGIH 99

MLTLARQQQRQNIRWLLCLSVLMLLALLLSLCAGEQWISPGDWFTPRGELFVWQIRLPRTLAVLLVGAALAISGAVMQALFENPLYPYDVPDYAFSTPVWISQAQGIRAGPGSSDKQEGEWPTGLRLSRIGGIH 111

MLTLARQQQRQNIRWLLCLSVLMLLALLLSLCAGEQWISPGDWFTPRGELFVWQIRLPRTLAVLLVGAALAISGAVMQALFENPLAEPGLLGVSNGAYPYDVPDYAFSTPVWISQAQGIRAGPGSSDKQEGEWPTGLRLSRIGGIH 123

MLTLARQQQRQNIRWLLCLSVLMLLALLLSLCAGEQWISPGDWFTPRGELFVWQIRLPRTLAVLLVGAALAISGAVMQALFENPLAEPGLLGVSNGAGVGLIAAVLLGQYPYDVPDYAFSTPVWISQAQGIRAGPGSSDKQEGEWPTGLRLSRIGGIH 135

MLTLARQQQRQNIRWLLCLSVLMLLALLLSLCAGEQWISPGDWFTPRGELFVWQIRLPRTLAVLLVGAALAISGAVMQALFENPLAEPGLLGVSNGAGVGLIAAVLLGQGQLPNWALYPYDVPDYAFSTPVWISQAQGIRAGPGSSDKQEGEWPTGLRLSRIGGIH 143

HA-tag
